# Ancient Loss of Catalytic Selenocysteine Spurred Convergent Adaptation in a Mammalian Oxidoreductase

**DOI:** 10.1093/gbe/evae041

**Published:** 2024-03-06

**Authors:** Jasmin Rees, Gaurab Sarangi, Qing Cheng, Martin Floor, Aida M Andrés, Baldomero Oliva Miguel, Jordi Villà-Freixa, Elias S J Arnér, Sergi Castellano

**Affiliations:** Great Ormond Street Institute of Child Health, University College London, London, UK; Division of Biosciences, University College London, London, UK; Department of Evolutionary Genetics, Max Planck Institute for Evolutionary Anthropology, Leipzig, Germany; Division of Biochemistry, Department of Medical Biochemistry and Biophysics, Karolinska Institutet, Stockholm, Sweden; Department of Biosciences, Faculty of Sciences and Technology, Universitat de Vic—Universitat Central de Catalunya, Vic, Spain; Department of Life Sciences, Barcelona Supercomputing Center (BSC), Barcelona, Spain; Division of Biosciences, University College London, London, UK; Department of Health and Experimental Sciences, Universitat Pompeu Fabra, Barcelona, Spain; Department of Biosciences, Faculty of Sciences and Technology, Universitat de Vic—Universitat Central de Catalunya, Vic, Spain; Institut de Recerca i Innovació en Ciències de la Vida i de la Salut a la Catalunya Central (IRIS-CC), Vic, Spain; Division of Biochemistry, Department of Medical Biochemistry and Biophysics, Karolinska Institutet, Stockholm, Sweden; Department of Selenoprotein Research, National Institute of Oncology, Budapest, Hungary; Great Ormond Street Institute of Child Health, University College London, London, UK; UCL Genomics, University College London, London, UK

**Keywords:** selenocysteine, convergent, selenoprotein, catalysis, adaptation

## Abstract

Selenocysteine, the 21st amino acid specified by the genetic code, is a rare selenium-containing residue found in the catalytic site of selenoprotein oxidoreductases. Selenocysteine is analogous to the common cysteine amino acid, but its selenium atom offers physical–chemical properties not provided by the corresponding sulfur atom in cysteine. Catalytic sites with selenocysteine in selenoproteins of vertebrates are under strong purifying selection, but one enzyme, glutathione peroxidase 6 (GPX6), independently exchanged selenocysteine for cysteine <100 million years ago in several mammalian lineages. We reconstructed and assayed these ancient enzymes before and after selenocysteine was lost and up to today and found them to have lost their classic ability to reduce hydroperoxides using glutathione. This loss of function, however, was accompanied by additional amino acid changes in the catalytic domain, with protein sites concertedly changing under positive selection across distant lineages abandoning selenocysteine in glutathione peroxidase 6. This demonstrates a narrow evolutionary range in maintaining fitness when sulfur in cysteine impairs the catalytic activity of this protein, with pleiotropy and epistasis likely driving the observed convergent evolution. We propose that the mutations shared across distinct lineages may trigger enzymatic properties beyond those in classic glutathione peroxidases, rather than simply recovering catalytic rate. These findings are an unusual example of adaptive convergence across mammalian selenoproteins, with the evolutionary signatures possibly representing the evolution of novel oxidoreductase functions.

**Significance**

Selenocysteine (Sec), the 21st amino acid, offers unique physical–chemical properties but has been independently lost in the GPX6 protein in several mammalian lineages. By reconstructing ancient mammalian proteins of GPX6, we show that exchanging Sec for its analogous amino acid cysteine (Cys) in this protein results in a loss of traditional catalytic function that is not regained over evolutionary time but is also accompanied by amino acid exchanges that are enriched for signatures of positive selection and shared across disparate lineages. Hence, we demonstrate a narrow evolutionary range following the Sec-to-Cys exchange and propose adaptive convergence of this protein across multiple mammalian lineages.

## Introduction

Catalytic residues are largely conserved in enzymes (Sharir-Ivry and Xia 2021) as they lower the activation energy of reactions and thereby can increase enzymatic turnover. Mutations in these evolutionarily constrained active sites typically reduce catalytic activity (Carter and Wells 1988; Loeb et al. 1989; Rennell et al. 1991), a proxy for fitness in enzymes, and are consequently often deleterious. Still, when such mutations do occur and persist, proteins often demonstrate evolutionary trajectories which either recover the rate of catalysis (Gromer et al. 2003) or open new protein functions (Jensen 1976; Jayaraman et al. 2022). Mutations that recover fitness, termed compensatory mutations, may not necessarily occur after a deleterious mutation to directly increase fitness but may also precede a deleterious mutation to prevent the loss of fitness (Jayaraman et al. 2022).

The evolutionary trajectories that follow a deleterious mutation, however, are limited by the enzyme's sequence, as natural selection favors mutations whose interactions with other residues compensate for catalytic loss or advance alternative properties, thereby increasing the fitness of the protein. Alongside epistatic interactions dictating the fitness of individual mutations, pleiotropy also plays a key role: trajectories that improve one enzymatic function but compromise another can decrease the fitness of a mutation and may be under purifying selection (Weinreich et al. 2006; Storz 2016). Such epistatic and pleiotropic constraints are often similar in orthologous proteins, whose sequence conservation among species provides similar genetic backgrounds for mutations and may result in natural selection favoring the evolution of the same sites across species.

**Fig. 1.**
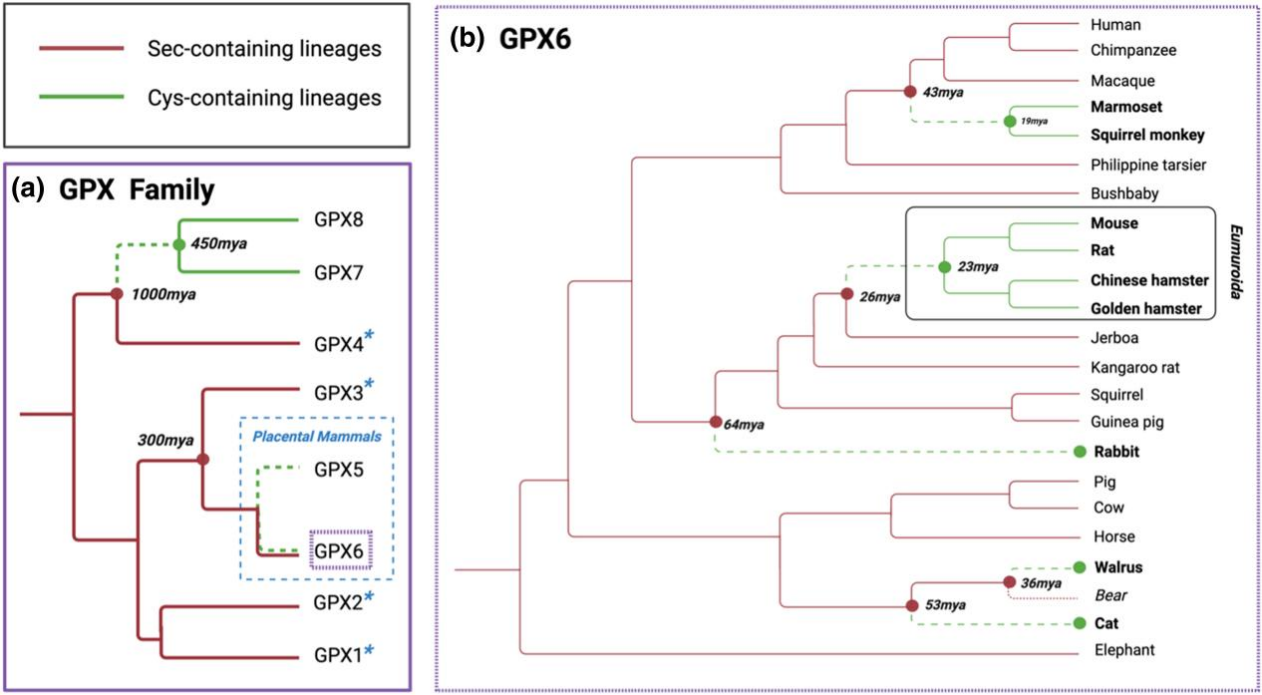
Phylogenies of the GPX family. a) The phylogeny of the GPX family in Eukaryotes, with selenoproteins present in the vertebrate ancestor indicated with a blue asterisk (based on Mariotti et al. (2012)). Includes the dates of the duplications leading to GPX7_Cys_, GPX8_Cys_, and GPX5_Cys_ and their older, single substitutions of Sec to Cys that resulted in enzymes with new properties. GPX5 and GPX6 are only present in placental mammals, indicated by the dashed blue box. b) The topology of the phylogeny of the 22 mammals in our analysis. In red, GPX6_Sec_ branches, in green, GPX6_Cys_ ones. Dashed green branches represent GPX6_Cys_ lineages where Sec was lost. Dotted red branch indicates the bear GPX6_Sec_ lineage, which was not used in the analysis due to sequence quality issues. The GPX6_Cys_*Eumuroida* clade, a specific group of muroid rodents, is boxed. Approximate ages given by Huchon et al. (2002), Steppan et al. (2004), Higdon et al. (2007), Hallström and Janke (2008), Chatterjee et al. (2009), and Nyakatura and Bininda-Emonds (2012).

Here, we study the selenoprotein glutathione peroxidase 6 (GPX6_Sec_), present only in placental mammals. We focus on the sporadic replacement, in several lineages, of the rare amino acid selenocysteine (Sec) for cysteine (Cys) (Kryukov et al. 2003) and its contrast to proteins of the GPX family using exclusively either Sec (GPX1, 2, 3, and 4) or Cys (GPX5, 7, and 8). Sec-to-Cys substitutions across orthologous selenoproteins, as seen in mammalian GPX6_Sec_, are unusual (Castellano et al. 2005) with other GPX_Cys_ proteins emerging only after duplication of a GPX_Sec_ gene (Fig. 1a; Mariotti et al. 2012). This is due to the low exchangeability of Sec and Cys in catalysis, likely due to the deleterious nature of the Sec-to-Cys mutations. Strong purifying selection (Castellano et al. 2009) is believed to act against the lower catalytic activity, lower nucleophilicity, and lower efficiency as a leaving group of Cys compared with Sec (Axley et al. 1991; Berry et al. 1992; Lee et al. 2000; Johansson et al. 2005; Arnér 2010; Kim et al. 2015; Reich and Hondal 2016), rendering these exchanges rare and likely opposed by natural selection.

Classic GPX_Sec_ activity reduces hydroperoxides, particularly hydrogen and lipid peroxides, with glutathione (GSH) as a cofactor. GPX_Cys_ proteins, from early duplications in early history of mammals (GPX5_Cys_ from GPX3_Sec_ duplication; around 300 million years ago [Mya]), vertebrates (GPX8_Cys_ from GPX7_Cys_ or GPX4_Sec_ duplication; probably 450 Mya), and metazoan (GPX7_Cys_ from GPX4_Sec_ duplication; more than 1,000 Mya) (Hedges 2002; Castellano et al. 2009; Trenz et al. 2021), have evolved a preference for other cofactors, for example, thioredoxin in GPX5_Cys_ or protein disulfide isomerase (PDI) in GPX7_Cys_ and GPX8_Cys_ (Nguyen et al. 2011). These Cys-containing proteins act on alternative substrates for peroxidation and may have additional functions, including signaling and oxidative protein folding (Nguyen et al. 2011; Taylor et al. 2013; Buday and Conrad 2021). Thus, while all GPX proteins may protect cells from oxidative damage (Tosatto et al. 2008), those proteins containing Cys may employ novel pathways to do so without being bona fide peroxidases, perhaps on account of their lower catalytic turnover. We ask if this is also the case for GPX6_Cys_.

## Results

### Increased Rate of Evolution Surrounding the Loss of Sec

We inferred 5 independent losses of Sec in GPX6_Sec_ (Fig. 1b, dashed green branches) across 22 mammals by reconstructing the ancestral sequence at each node of their phylogeny with PAML (supplementary fig. S1, Supplementary Material online; Yang 2007). These inferred losses occurred in the last 64 million years (Fig. 1b, dashed green branches; Huchon et al. 2002; Steppan et al. 2004; Higdon et al. 2007; Hallström and Janke 2008; Chatterjee et al. 2009; Nyakatura and Bininda-Emonds 2012) and resulted in multiple GPX6_Cys_ lineages. We evaluated the impact of natural selection by calculating independent dN/dS ratios (Yang 2007) for each branch of the mammalian tree (Fig. 1b), including ancestral branches. Here, we excluded the Sec-to-Cys site from calculations of the dN/dS ratio to only capture amino acid evolution accompanying the loss of Sec. Indeed, the dN/dS ratios appear larger in GPX6_Cys_ lineages compared with neighboring GPX6_Sec_ lineages, suggesting faster evolution along the branches with Cys (supplementary fig. S2, Supplementary Material online). We tested this hypothesis with a branch model likelihood ratio (LR) test PAML (Yang 2007) and found that contrasting the dN/dS ratios of GPX6_Cys_ lineages in the branches where Sec was lost (Fig. 1b, dashed green branches) to GPX6_Cys_ lineages in the branches inheriting this loss (Fig. 1b, solid green branches) and GPX6_Sec_ lineages (Fig. 1b, solid red branches) does indeed support a higher dN/dS ratio in the branches where Sec was substituted for Cys (LR test; *P* = 0.002; dN/dS = 0.370 dashed green vs. 0.279 solid green vs. 0.217 solid red branches in Fig. 1b). Therefore, significant additional amino acid evolution must have accompanied the loss of Sec.

However, dN/dS inflation across GPX6_Cys_ lineages is still under 1, which does not clearly indicate that positive selection has acted along the branches where Sec was lost, rather than relaxed constraint in GPX6_Cys_. Since positive selection acting on GPX6_Cys_ enzymatic properties would mainly impact the catalytic domain, which is otherwise under strong constraint, it is unsurprising that the dN/dS ratio across the whole gene does not reach 1. We thus separately performed the LR test in its three domains: the N-terminus, the GPX domain, and the C-terminus, as defined in the Pfam database (Mistry et al. 2021). The GPX domain and, to a lesser extent, the C-terminus domain are essential for the activity of the enzyme, with two (U/C, Q) and two (W, N) key catalytic residues in the GPX and C-terminus domains, respectively, making a catalytic tetrad (Toppo et al. 2008; Tosatto et al. 2008; Cheng and Arnér 2017), which we found conserved across GPX6_Sec_ and GPX6_Cys_ lineages. In contrast, the N-terminus is not considered essential for catalysis.

As expected, constraint grows from the N-terminus, to the C-terminus, and to its highest degree in the GPX domain, as indicated by their dN/dS ratios (Table 1). However, the dN/dS ratio of the GPX domain is significantly larger in GPX6_Cys_ lineages at the time Sec was lost when compared with the other branches (LR test; *P* = 2 × 10^−5^; dN/dS = 0.384 dashed green vs. 0.186 solid green vs. 0.130 solid red branches in Fig. 1b), a pattern not observed in the N- and C-terminus. This is again suggestive of an increased level of evolutionary change on the active GPX domain surrounding the time when Sec is abandoned in catalysis.

**Table 1:** dN/dS ratios across lineages, proteins, and protein domains

			dN/dS in branches where GPX6 has				Convergent sites
Protein	Region	Sec	Exchanged Sec for Cys	Inherited Cys	All	*P*-value	Number
GPX6_Cys_	**Full length**	0.217	**0**.**370**	0.279	0.256	**0**.**002**^a^	**22**
	N-terminus	0.436	0.411	0.671	0.460	0.268	3
	**GPX domain**	0.130	**0**.**384**	0.186	0.184	**2 × 10^−5^** ^b^	**12**
	C-terminus	0.174	0.258	0.250	0.203	0.157	7
GPX1_Sec_	GPX domain	0.064	0.040	0.069	0.060	0.534	0
GPX2_Sec_		0.075	0.042	0.038	0.060	0.191	0
GPX3_Sec_		0.094	0.108	0.056	0.091	0.439	1
GPX4_Sec_		0.062	0.007	0.203	0.061	1 × 10^−4b^	0
GPX5_Sec_		0.233	0.145	0.219	0.212	0.227	4
GPX7_Cys_	GPX domain	0.083	0.080	0.117	0.088	0.712	0
GPX8_Cys_		0.223	0.155	0.198	0.207	0.616	0

dN/dS ratios in lineages where GPX6 has Sec (Fig. 1b, solid red branches), has gained Cys (Fig. 1b, dashed green branches), or inherited it (Fig. 1b, solid green branches) and the number of identified convergent sites between lineages where GPX6 has gained Cys (Fig. 1b, dashed green branches). dN/dS ratios and number of identified convergent sites for the GPX domain in other GPX proteins. The LR test contrasts one ratio for all branches (null hypothesis) to different ratios among groups of branches. *P*-values are obtained from a $\chi^{2}$ distribution with df = 2. In bold when significant and accompanied by sites under convergent evolution across GPX6_Cys_ lineages.

^a^ *P* < 0.005. ^b^*P* < 0.0005.

### Localized Evolutionary Signatures to the Sec-to-Cys Exchange

We investigated whether this observation was exclusive to GPX6_Cys_ and therefore indicative of rapid evolution associated with the Sec-to-Cys exchange rather than on the overall antioxidant function of the GPX domain, as others have suggested (Tian et al. 2021). Indeed, when isolating this domain within other enzymes in the GPX family, we found no evidence of dN/dS inflation (Table 1; supplementary table S1, Supplementary Material online) in the lineages where Sec was lost in GPX6 (analogous dashed green branches in Fig. 1b in other GPXs). Within the other enzymes, there is only a significant inflation of this domain in GPX4_Sec_ (Table 1) in the lineages that contain the loss of Sec in GPX6 (analogous solid green branches in Fig. 1b in GPX4_Sec_), unrelated to the Sec-to-Cys exchange. We reason that the dN/dS ratio of the GPX domain in GPX6_Cys_ at the time of Sec loss is unusually large for proteins of the GPX family.

We then asked whether the higher dN/dS is due to positive selection on individual sites that accompany the substitution of Sec for Cys in GPX6_Cys_. We contrasted GPX6_Cys_ lineages for this domain at the time of Sec loss (Fig. 1b, dashed green branches) with all other lineages and found a branch-site model LR test (PAML; Yang 2007) significant (LR test; *P* = 0.046; supplementary table S2, Supplementary Material online). This is suggestive of sites under positive selection in the GPX domain when Sec was lost.

Given the sequence conservation observed in orthologous proteins such as GPX6, we then consider if epistatic and pleiotropic constraints are shared over GPX6_Cys_ lineages. By extension, we consider if the fitness of mutations, which depend on their interaction with the genetic background and their effects on multiple functions, are similar across the orthologous proteins and if the same mutations may be repeatedly under selection. To address this, we ask if many of the same amino acid exchanges are shared amongst the mammalian lineages where Cys is lost. From here on, these are referred to as convergent sites.

To identify these convergent sites, we use a method that uses the alignments of the GPX6_Cys_ proteins with the known mammalian phylogeny to infer ancestral proteins and identify sites which have changed to the same amino acid or repeatedly changed over pairs of GPX6_Cys_ lineages (CONVERG2; Zhang and Kumar 1997). These identified convergent sites are most common between lineages where Sec was lost (Fig. 1b, dashed green branches) (supplementary table S3, Supplementary Material online). We therefore suggest that, following the loss of Sec, positive selection acts mostly on a particular subset of mutations.

### Convergence Concentrates in the Catalytic Domain

Most convergent sites are found in the GPX domain and the least in the N-terminus (54.6% in the GPX domain, followed by 31.8% and 13.6% in the C-terminus and N-terminus, respectively). This approximately matches the lengths of each domain (113 sites of the GPX domain compared with the 65 and 39 sites of the C-terminus and N-terminus, respectively), despite the relatively higher constraint expected on the GPX domain. Moreover, convergence is largely subdued in the GPX6_Cys_ lineages inheriting the loss of Sec (Fig. 1b, solid green branches) and minimal in the GPX6_Sec_ lineages and other GPX_Sec_ and GPX_Cys_ enzymes (supplementary tables S3 to S10 and fig. S3, Supplementary Material online). Further, simulations of protein evolution incorporating the accelerated rate of amino acid change in GPX6_Cys_ sequences, including the GPX domain, cannot reproduce the pattern of convergence observed between these lineages at the time of Sec loss (Seq-gen (Rambaut and Grassly 1997); supplementary figs. S7 to S8, Supplementary Material online). However, these simulations show that the few weak convergence signatures in other GPXs are as expected, based on their respective rate of amino acid change (supplementary figs. S9 to S15, Supplementary Material online), and we presume they result from chance. We therefore infer that signatures of convergence are exclusive to GPX6_Cys_ and strongest in its GPX domain at the time that Sec was lost. This suggests that GPX6_Cys_ lineages have similar evolutionary trajectories following the Sec-to-Cys exchange and a narrow path in which to restore fitness, which may be in part due to few epistatically relevant differences in the genetic backgrounds of GPX6 proteins amongst placental mammals.

### Strong Convergence between Most Related GPX6 Lineages with Cys

**Fig. 2.**
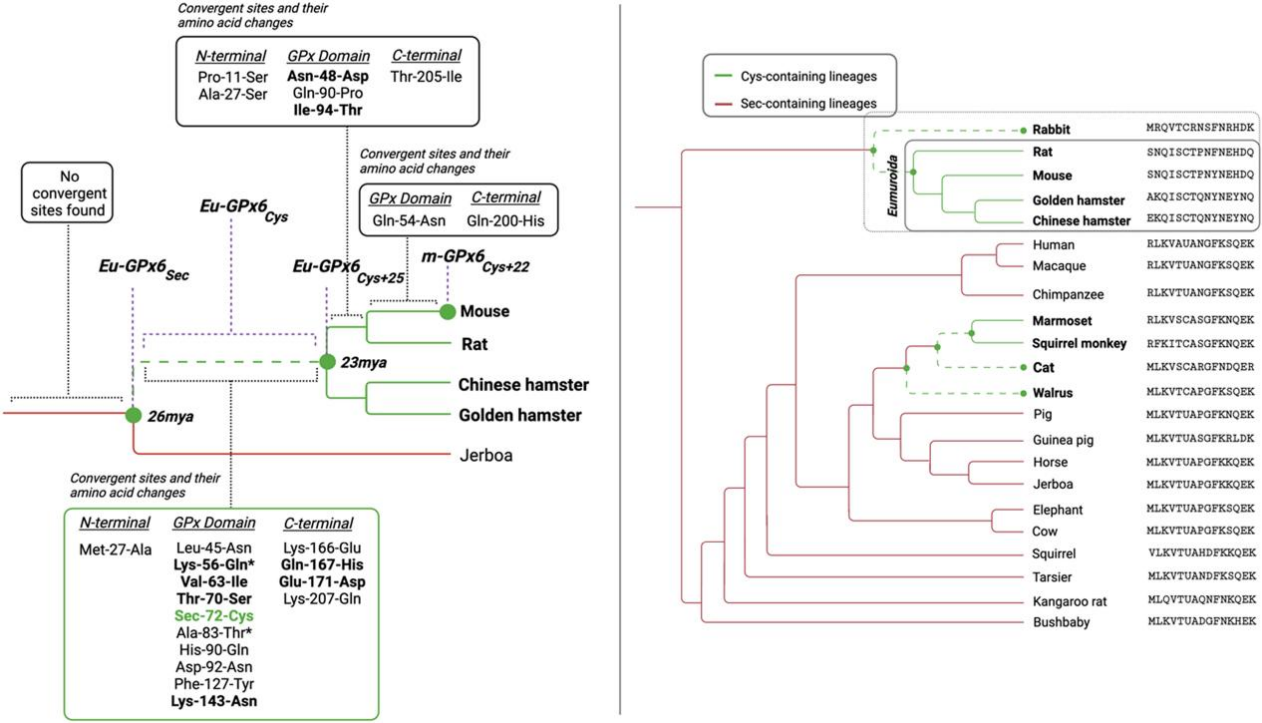
Convergence in the GPX6 phylogeny. a) Topology of the phylogeny of the *Eumuroida* GPX6_Cys_ clade, green branches, with the Jerboa GPX6_Sec_ lineage, red branch, as an outgroup. The basal *Eumuroida* lineage, dashed green branch, abandoned Sec in catalysis for Cys with a burst of 25 additional amino acid changes 23 to 26 Mya. Fourteen of these 25 changing amino acid sites, plus the Sec-to-Cys site, have signatures of convergence (CONVERG2; Zhang and Kumar 1997) across GPX6_Cys_ lineages (Fig. 1b, green branches). Sites that have repeatedly changed in the GPX6_Cys_ lineages toward similar or the same amino acid are shown in bold (green box). No convergent sites are found immediately before the loss of Sec and only few immediately after. Further, the * denotes sites with a posterior probability of positive selection in the upper 90th percentile across the GPX domain in GPX6_Cys_ lineages, which are significantly enriched at the time Sec was abandoned. b) Topology of the phylogenetic tree, with midpoint rooting, from the 14 convergent sites accompanying the Sec-to-Cys substitution (sites corresponding to the positions given in the large green box in Fig. 2a, shown to the left of the phylogeny) in the basal *Eumuroida* GPX6_Cys_ lineage (Fig. 2a, dashed green branch). In sharp contrast to the species phylogeny (Fig. 1b), the GPX6_Sec_ lineages now form two clades.

While convergent sites are identified amongst all GPX6_Cys_ lineages (supplementary table S3, Supplementary Material online), we inferred the highest level of convergence between the basal *Eumuroida* (Fig. 1b, dashed green line in box) and its genetically closer GPX6_Cys_ lineages, particularly the rabbit lineage (supplementary fig. S3 and table S3, Supplementary Material online). To maximize our power to identify signatures of convergence amongst GPX6_Cys_ lineages, we therefore focus on the sites that change along the root of *Eumuroida*. We find 25 sites at the root of the *Eumuroida* (Fig. 2a, dashed green branch) that changed alongside Sec and found that 14 of them bear signatures of convergence across the GPX6_Cys_ lineages (Fig. 2a, green box). These convergent sites are, again, mainly in the GPX catalytic domain, 64.3% of them (supplementary table S2, Supplementary Material online), and are enriched for the signatures of positive selection that we observe along branches leading to *Eumuroida*, rabbit, and marmoset–squirrel monkey (Mann–Whitney *U* test, *P* = 1.573e^−7^; Yang et al. 2005). We also find signatures of positive selection, albeit weaker, in convergent sites in the GPX6_Cys_ lineages with the loss of Sec (Mann–Whitney *U* test, *P* = 0.007) (Fig. 2a, solid green branches) but not preceding it, in agreement with adaptive convergence acting on the Sec-to-Cys exchange.

Because adaptive convergence can mimic shared ancestry (Edwards 2009), it may distort the topology of the species phylogeny (Fig. 1b), and indeed, we found that a tree reconstructed (PhyML; Guindon et al. 2010) from the GPX domain pulls the rabbit lineage closer to the *Eumuroida* clade (supplementary fig. S4d, Supplementary Material online). This, to a lesser extent, is also observed with the C-terminus (supplementary fig. S4e, Supplementary Material online), which contributes to catalysis, but is not observed with the N-terminus domain (supplementary fig. S4c, Supplementary Material online) nor with other GPX proteins (supplementary fig. S5, Supplementary Material online). The strongest departure from the species tree is reconstructed from the 14 convergent sites changing at the root of the *Eumuroida* 23 to 26 Mya (Huchon et al. 2002), plus the Sec-to-Cys substitution (Fig. 2b). In this tree, despite their large divergence, the GPX6_Cys_ species form two clades. One clade shows the rabbit lineage sharing a most recent common ancestor, to the exclusion of all other species, with the 64 million years apart *Eumuroida*, and the other clade groups the remaining 100 million years apart from GPX6_Cys_ lineages (Hallström and Janke 2008). This supports a role for convergence (Edwards 2009) in driving adaptive changes, perhaps compensating for loss of enzymatic activity, or opening new properties, as it seems to have happened with GPX5_Cys_, GPX7_Cys_, and GPX8_Cys_ enzymes that lost Sec much earlier (supplementary fig. S1a, Supplementary Material online) (Herbette et al. 2007; Chen et al. 2016).

### Loss of Activity in Reconstructed Ancient GPX6 Proteins with Cys

The catalytic activity of enzymes that have exchanged Sec to Cys has been shown to decrease (Gromer et al. 2003). We thus expect to recapitulate this here: functional catalytic activity of Eu-GPX6_Sec_ and a nonfunctional or reduced catalytic activity of Eu-GPX6_Cys_. Given the signatures of adaptive convergence in *Eumuroida* following the loss of Sec, we also hypothesize the same compensatory mutations have been under natural selection across multiple mammalian lineages losing Sec to recover catalytic function in GPX6_Cys+25_.

To evaluate this, we reconstructed three ancient proteins inferred to exist 23 to 26 Mya at the root of this clade (Fig. 2a, dashed green branch) and assessed their catalytic activity experimentally and computationally. These proteins are (i) the ancestral protein before the loss of Sec, Eu-GPX6_Sec_, taken from the common ancestor of the *Eumuroida* and Jerboa species 26 Mya (Huchon et al. 2002); (ii) the same ancestral protein with Cys instead of Sec, Eu-GPX6_Cys_; and (iii) the ancestral but later-day protein with Cys and 25 other amino acids changes, Eu-GPX6_Cys+25_, taken from the common ancestor of the *Eumuroida* species 23 Mya (15 of 26 these amino acid changing sites, including the Cys site, have signatures of adaptive convergence; Fig. 2a). A further modern protein and 2 synthetic variants were also assessed: the modern mouse protein, m-GPX6_Cys+22_, with 22 additional amino acid changes (19 substitutions and a 3 C-terminal extension) from Eu-GPX6_Cys+25_ and no clear signatures of adaptive convergence (Fig. 2a), the modern mouse protein with Cys mutated to Sec (m-GPX6_Sec+22_), and the modern mouse protein with Cys mutated to redox inactive serine (Ser; m-GPX6_Ser+22_) for comparisons of activity with the Sec and Cys variants.

**Fig. 3.**
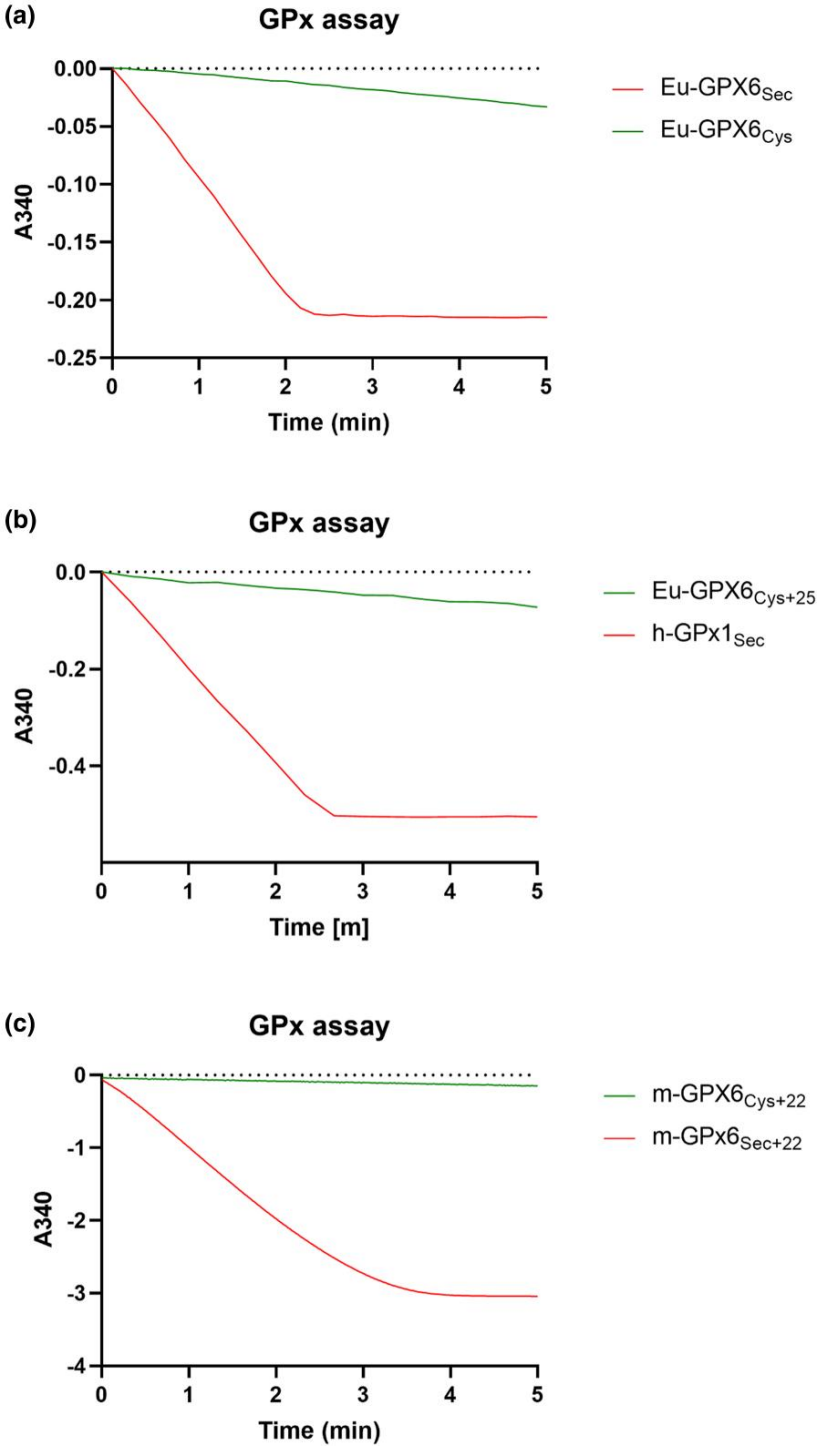
Experimental assessment of catalytic ability in ancestral and modern GPX6 proteins. a) Experimental assessment of peroxidase reaction with H_2_O_2_ as a substrate for ancient Eu-GPX6_Sec_ (red) and Eu-GPX6_Cys_ (green). NADPH consumption by GR is indicated by the decrease in absorbance at 340 nm over time in the coupled assay (see Materials and Methods for further details). b) Equivalent assay for ancient Eu-GPX6_Cys+25_ (green), which has very limited activity compared with human GPx1 (red) used here as a positive control. c) Equivalent assay for modern m-GPX6_Cys+22_ (green), again with scant activity, which is recovered once this protein is mutated to contain Sec, m-GPX6_Sec+22_ (red).

We reconstructed these ancient and modern proteins and produced them as recombinant proteins heterologously expressed in *Escherichia coli*. The Sec insertion system in bacteria is noncompatible with mammalian selenoprotein-encoding genes, hampering the production of proteins with Sec; we thus employed a method we recently developed utilizing UAG redefined as a Sec codon in a release factor 1–deficient *E. coli* host strain lacking other UAG codons (Cheng and Arnér 2017). We first compared the catalytic activity of Eu-GPX6_Sec_ and Eu-GPX6_Cys_ with H_2_O_2_ as the peroxide substrate and GSH as the reducing agent, with the expectation that substitution of Sec for Cys would lower its turnover. Indeed, the ancient Eu-GPX6_Sec_ protein displays the classic peroxidase activity of Sec-containing GPX enzymes, whereas Eu-GPX6_Cys_ had almost no activity for this reaction (Fig. 3a).

We therefore observe the hypothesized a large drop in catalysis from Eu-GPX6_Sec_ to Eu-GPX6_Cys_. Along the same basal *Eumuroida* lineage, we observe signatures of adaptive evolution (Fig. 2a), which may be assumed to then recover such a lost activity. However, when measuring the catalytic activity in Eu-GPX6_Cys+25_ on H_2_O_2_ with GSH, we do not observe the classic GPX activity to be recovered. Hence, the 25 additional changes along the basal *Eumuroida* lineage, inferred to be under adaptive convergence, do not appear to recover the original enzymatic function.

### Classic GPX Activity Unobserved in the Modern Mouse Protein

We then evaluated the activity of the extant m-GPX6_Cys+22_ protein (Fig. 2a), which is 90% identical to Eu-GPX6_Cys+25_. This protein is expressed in the mouse embryo, testis, olfactory epithelium, and brain (Kryukov et al. 2003; Shema et al. 2015; Goltyaev et al. 2020) with clear function (knocking down in modern mice results in neurological consequences (Shema et al. 2015)). However, this Cys-containing variant also lacks classic GPX activity with H_2_O_2_ and GSH (Fig. 3c), with GPX activity also unobserved when testing the additional substrate cumene hydroperoxide (supplementary fig. S15, Supplementary Material online). This suggests that its modern function lies outside of that of a classic GPX protein.

It can be suggested that increased expression of the Cys-containing proteins could recover catalytic activity to a functional level. However, the activity of the extant m-GPX6_Cys+22_ protein displays no detectable peroxidase activity (activity below −0.02A340; supplementary fig. S16, Supplementary Material online), almost identical to that of the redox inactive synthetic m-GPX6_Ser+22_ protein. Increasing the expression of the Cys protein, even by a factor of ten or higher, is therefore unlikely to result in a meaningful enzymatic activity and would not be expected to recover classic GPX function. Hence, we suggest the observed adaptive changes in the evolution of this protein do not simply act to recapitulate Sec activity. Instead, we propose that the signatures of adaptive convergence represent the convergent evolution of a new, as of yet, underdetermined function, as observed in other GPX proteins that have lost Sec (Herbette et al. 2007; Chen et al. 2016).

### Binding of GSH Unaffected by Acquisition of Cys

Interestingly, classic GPX activity was reacquired when Cys was mutated back into Sec, producing the synthetic m-GPX6_Sec+22_ variant (Fig. 3c). We therefore asked if classic GPX activity was likely to be recovered with the replacement of Cys with Sec in all ancestral Cys proteins. To assess this, we computationally assessed the binding of GSH to the Eu-GPX6_Cys_, Eu-GPX6_Cys+25_, and m-GPX6_Cys+22_ proteins by running protein–ligand binding energy landscape explorations via the PELE software (Borrelli et al 2005), using GSH and GSH disulfide as ligands.

**Fig. 4.**
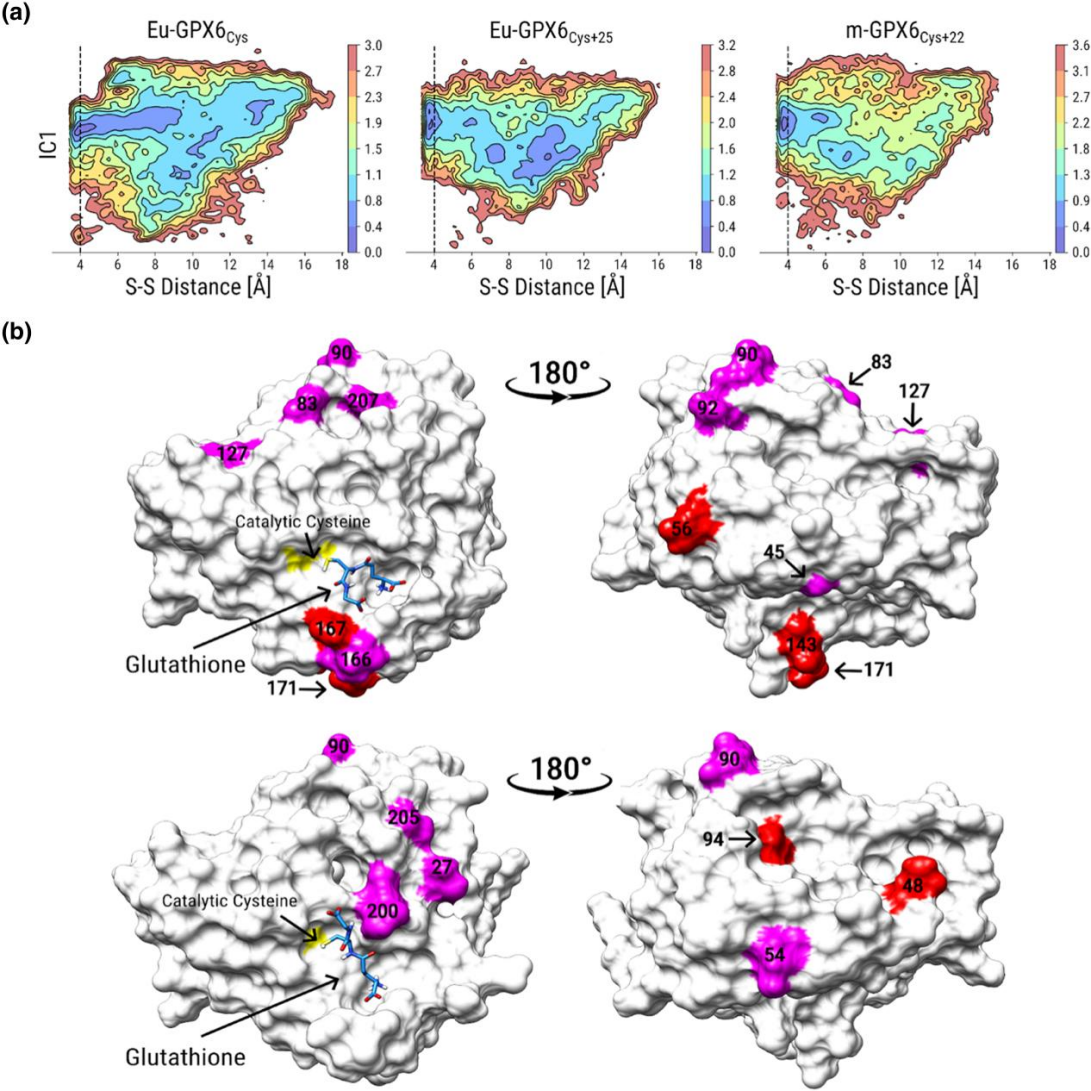
Computational analysis of enzyme structure and catalytic reaction. a) Free energy profiles for the docking of GSH to Eu-GPX6_Cys_ (left), Eu-GPX6_Cys+25_ (center), and m-GPX6_Cys+22_ (right). The *x* axis represents the distance between the catalytic Cys sulfur atom and the ligand's sulfur atom, while the *y* axis shows the slowest TICA coordinate (IC1). The vertical dashed line represents a 4 Å distance, with the free energy minimum in the three enzymes within this reactive catalytic distance. b) Convergence patterns (Fig. 2a) from Eu-GPX6_Cys_ to Eu-GPX6_Cys+25_ (top) and from Eu-GPX6_Cys+25_ to m-GPX6_Cys+22_ (mouse-GPX6) (bottom). Sites converging toward similar (magenta) or the same (red) amino acids are shown with their sequence position. The catalytic Cys (yellow) is shown with the GSH best binding energy conformation (green) sampled during docking simulations.

Models for all proteins were obtained using the AlphaFold2 algorithm (Jumper et al. 2021) and showed no significant structural changes in their overall fold shape when compared among them (largest alpha-carbon RMSD: 2.1 Å over 218 residues). The reactive free energy minimum was inferred by plotting the free energy of the sampled trajectories along the slowest kinetic coordinate computed for all our Monte Carlo simulations (IC1) and the reactive distance between the sulfur atoms of GSH and the catalytic Cys (Fig. 4a). These simulations used a protocol that first diversifies the possible GSH binding modes around the catalytic residues to then further explore the lowest reactive energy configurations uncovered by this first broad sampling. This adaptive sampling technique ensures more convergent results despite the initial ligand starting position (Gilabert et al. 2019).

The energy landscapes inferred for Eu-GPX6_Cys_, Eu-GPX6_Cys+25_, and m-GPX6_Cys+22_ all show similar minima at reactive distances (a vertical dashed line is used as threshold at 4 Å). Since the IC1 composite coordinate is constructed from a common reference for the ligand Cartesian coordinates for all simulations (see Materials and Methods), we infer that all three variants have similar binding modes for GSH. All variants also show similar values at their binding energy minima in the reactive region (supplementary fig. S17, Supplementary Material online), thereby indicating they interact with similar strengths with GSH.

Our computational analysis thus suggests that the binding of GSH and overall structures of the enzymes have not been adversely affected by the acquisition of Cys or other mutations. Further, the convergent amino acid substitutions, possibly facilitating a new function of the protein, are mainly located on the enzyme's surface (Fig. 4b). Given that this is the case for GPX6 in other lineages losing Sec (supplementary fig. S17, Supplementary Material online), we again suggest that a new function is shared across all mammalian GPX6 proteins having lost Sec.

## Discussion

Substituting Sec for Cys in GPX6, and thereby abandoning selenium for sulfur, leads to a burst of evolutionary activity in lineages sharing this exchange. This may be an example of an “evolutionary Stokes shift,” where the evolutionary activity of a protein increases following a significant amino acid exchange, and new mutations may gradually increase the fitness of the original substitution over time (Pollock et al. 2012). The amino acid changes are not only concentrated in the functional domain but are often shared across GPX6_Cys_ lineages, suggesting a narrow evolutionary range for GPX6 to maintain or increase fitness when losing Sec. As supported by increased convergence between the closest related lineages, this is likely a path under both epistatic and pleiotropic constraints; the fitness of mutations is dependent on interactions with other conserved catalytic sites and preservation of other key enzymatic properties (Sharir-Ivry and Xia 2021).

We initially hypothesized that the observed signatures of adaptive convergence represented a recovery of catalytic activity across GPX6_Cys_ lineages. Indeed, thioredoxin reductase in *Drosophila melanogaster* has been shown to recover up to 50% of its catalytic activity with compensatory mutations following its loss of Sec (Gromer et al. 2003), and we viewed it possible that compensatory mutations also acted to restore catalytic activity here. However, classic GPX activity is not recovered by the amino acid changes along the *Eumuroida* lineage, nor is it likely to be compensated by the higher expression of Cys when the activity is undetectable. Still, contemporary GPX6_Cys_ proteins are functional, expressed in the mouse embryo, testis, olfactory epithelium, and brain (Kryukov et al. 2003; Shema et al. 2015; Goltyaev et al. 2020), and when knocked down result in a deleterious neurological phenotype (Shema et al 2015). The observed signatures of adaptive convergence across GPX6_Cys_ proteins therefore do not represent a recovery of classic GPX function but a shared evolutionary trajectory requiring a more considered explanation.

Losing Sec, and with it classic GPX function, may have widened the number of tolerable mutations in the highly similar functional domain of GPX6_Cys_ proteins, resulting in the observed increase of evolutionary rate and similarly mutated sites across mammalian lineages. The contemporary function of GPX6_Cys_ proteins, in this scenario, would therefore not be novel and would instead represent a maintained ancestral function of which drives the still-narrow range of tolerable mutations. The apparent tolerable loss of Sec itself remains a question, but it is of course possible that the activity of other Sec-containing GPX proteins compensate for the loss of classic GPX activity to the extent that catalytic function of the protein may be lost.

Alternatively, the observed signatures of adaptive convergence may represent shared evolutionary trajectories toward novel properties or novel substrates. Indeed, other Cys-containing GPX proteins act on alternative substrates for peroxidation (Nguyen et al. 2011; Taylor et al. 2013; Buday and Conrad, 2021), and it is possible that GPX6_Cys_ has also evolved a preference for a novel substrate, one differing from hydrogen peroxide and cumene hydroperoxide tested here, for this ultimate function. Thus, perhaps similar to old and single losses of Sec occurring hundreds of millions of years ago in GPX5_Cys_, GPX7_Cys_, and GPX8_Cys_ enzymes (Trenz et al. 2021), we suggest that GPX6_Cys_ proteins may have also gained yet unidentified abilities, though acquired more recently, independently, and convergently across lineages, instead of just recovering the catalytic rate of their previous reaction. Further, GPX6_Cys_ proteins across mammals appear to be able to recover their classic GPX function with Sec, which we view as in agreement with the loss of Sec resulting in subtly different, but related, catalytic properties in an enzyme now apparently devoid of its classic function. Again, we propose it likely that the activity of other Sec-containing GPX proteins compensated for the immediate loss of classic GPX activity when Sec was lost in GPX6, but here, this allowed mutations to accumulate along GPX6_Cys_ lineages and novel properties to develop. Only comprehensive functional characterizations of these individual GPX6_Cys_ enzymes, and their interactions with other GPX proteins, in mammals will provide insights into their current functional roles, be that those related to peroxidation or entirely novel or undocumented.

In conclusion, we present the first evidence for molecular convergence of changes in proteins when abandoning unusual selenium in catalysis for common sulfur, hence ablating activity. These concerted changes follow a certain path, maintaining some enzymatic properties and possibly adding new ones. Because multiple nonvertebrate species have completely abandoned enzymatic selenium for sulfur, we wonder whether other convergent adaptations leading to uncharted functions remain hidden in nature.

## Materials and Methods

The GPX6 coding sequences and proteins (alongside those for other members of the GPX family) for 22 present-day mammal species were taken from SelenoDB 2.0 (Romagné et al. 2014) or Ensembl (Yates et al. 2020). Of these species, nine contain Cys instead of Sec as their catalytic residue of GPX6 (Fig. 1). We aligned the coding sequences using MAFFT (Katoh et al. 2019), and uncertain positions with an average posterior probability below 0.95, as calculated by HMMER (Durbin et al. 1998; Potter et al. 2018), were removed from further analysis.

We used PAML (Yang 2007) to reconstruct ancestral nodes across the mammalian GPX6 tree and to infer independent losses of Sec. Here, the accuracy of ancestral node reconstruction for all but one node is estimated to be above 96% (with the outlier node being the most basal, with an accuracy of 88.45%; see supplementary fig. S1, Supplementary Material online). The ancestral sequences were also then corroborated with Ancestor v1.1 (Diallo et al. 2010) and FastML (Moshe and Pupko 2019) (see Supplementary Material online). Given this multiple-pronged approach in reconstructing ancestral sequences and the well-resolved mammalian phylogeny, the ancestral sequences are reported with confidence. Independent dN/dS ratios for each mammalian was computed using the free-ratio model of the CODEML package from PAML (Yang 2007), whereas the branch model was used to explicitly test if an increased rate of evolution occurred in specific groups of lineages. We used the latter to compare dN/dS between branches with Sec (Fig. 1; solid red branches), branches where Sec is exchanged for Cys (Fig. 1, dashed green branches) and branches where Cys is maintained (Fig. 1, solid green branches). This was repeated for the three separate domains of the protein: N-terminus, GPX domain, and C-terminus. We computed the LR of our branch model to the null model (which assumes a singular dN/dS value across branches) and used this to calculate the significance of difference in fit between the two models (Table 1). This was repeated for other genes in the GPX family, testing for differences in dN/dS across analogous groups of lineages. We tested for selection acting on individual sites across specific branches using the branch-site model in PAML (Yang 2007) both across the entire protein and on the catalytic GPX domain.

Based on the alignments of the GPX6_Cys_ proteins and mammalian phylogeny, we then consider each pair of GPX6_Cys_ lineages and identify sites that have changed from the common ancestor along both lineages, now termed convergent sites (supplementary table S3, Supplementary Material online). This was done using the CONVERG2 program (Zhang and Kumar 1997), which infers ancestral proteins along the mammalian phylogeny to count the convergent changes for each pair of lineages (as well as calculating the expected frequencies of convergent changes between pairs of lineage). We further compared these expected frequencies to the observed frequencies of convergent changes for each pair of GPX6_Cys_ lineages.

Where the sequences for the species containing Cys in GPX6 were available, equivalent analyses were run on other members of the GPX family. We tested for enrichment of signatures of positive selection, as calculated from the branch-site model in PAML along the branch leading to squirrel monkey–marmoset, the Eumuroida branch, and the branch leading to rabbit, across these convergent sites using a Mann–Whitney *U* test. We also tested enrichment of selection signatures in sites inferred to have changed over the *Eumuroida* branch and with signatures of convergence in GPX6_Cys_ lineages (Fig. 2a). PHYML was used to reconstruct the mammalian tree according to the 14 identified sites (Guindon et al. 2010) (Fig. 2b).

The evolution of the GPX6 protein sequence was simulated using Seq-Gen (Rambaut and Grassly 1997), using our inferred mammalian ancestral sequence, the JTT model of amino acid substitution, and tree lengths given by the rate of amino acid changes (taken as the dN value calculated from the CODEML package in PAML (Yang 2007). The distribution of convergent changes, as calculated from CONVERG2 (Zhang and Kumar 1997), from 1,000 simulation runs, was then plotted and compared with the observed number of convergent site changes (supplementary fig. S6, Supplementary Material online). The equivalent methodology was run for the catalytic GPX domain, as well as for other GPX proteins.

We reconstructed the Eu-GPX6_Sec_, Eu-GPX6_Cys_, and Eu-GPX6_Cys+25_ proteins (Fig. 2a), as inferred from PAML (Tian et al. 2021), from heterologous expression in *E. coli*. The catalytic activities of these proteins, and the modern mouse protein, were evaluated by measuring the peroxidation activity on H_2_O_2_. The sequences of the reconstructed ancient proteins and recombinant proteins, along with purification and assay protocols, are given in Supplementary Material online.

Using AlphaFold2 (Jumper et al. 2021), we built structures for the GPX6 orthologs and ancestral sequences to run protein–ligand binding energy landscape explorations using the PELE software (Borrelli et al. 2005), using GSH and GSH disulfide as ligands. Structures were first prepared with the Protein Preparation Wizard of the Schrodinger suite (Sastry et al. 2013) by setting the protonation state at pH 7. Initial conformations were computed with the GLIDE algorithm (Friesner et al. 2006), and the best-scoring conformations were selected among conformations with a reactive distance, between the ligand and the catalytic Cys sulfur atoms (S-S distance), lower than 4 Å. From these starting conformations, a first PELE simulation was run to identify catalytic poses with low global energies and reactive distances below a 4 Å threshold. This first simulation equilibrates the system without ligand constraints to obtain a diversified set of low-energy configurations. We used the lowest binding energy poses within the S-S distance threshold to run a second PELE simulation, focusing on the sampling on this reactive region. All obtained simulation trajectories were aligned to a common coordinate frame of reference by aligning each frame to the same protein structure. A time-structure independent component analysis (TICA) was performed to find the common slowest-relaxing feature combination (Molgedey and Schuster 1994) with the PyEMMA library (Scherer et al. 2015) by using as features only the ligand Cartesian coordinates. The probabilities of visiting the slowest TICA coordinate (IC1) according to the S-S distance were plotted as free energy maps for each simulation (Fig. 4 and supplementary fig. S17, Supplementary Material online).

## Supplementary Material

evae041_Supplementary_Data

## Data Availability

The data underlying this article are available in the article and in its online Supplementary Material online.
