# Supplementary material for: Ancient Loss of Catalytic Selenocysteine Spurred Convergent Adaptation in a Mammalian Oxidoreductase
Source: Genome Biol Evol. 2024 Mar 6;16(3):evae041. doi: 10.1093/gbe/evae041 (PMC10958145; doi:10.1093/gbe/evae041)
Supplement: evae041_Supplementary_Data [file evae041_supplementary_data.zip › GBE_edited_supp_Rees.docx]

**Supporting Information for**

**Ancient loss of catalytic selenocysteine spurred convergent adaptation in a mammalian oxidoreductase**

Jasmin Rees^1,2*#^, Gaurab Sarangi^3*^, Qing Cheng^4*^, Martin Floor^5,6*^, Aida M Andrés^2^, Baldomero Oliva Miguel^7^, Jordi Villà-Freixa^5,8^, Elias SJ Arnér^4,9^, Sergi Castellano^1,10#^

**Affiliations:**

^1^ Great Ormond Street Institute of Child Health, University College London, London, United Kingdom

^2^ Division of Biosciences, University College London, London, United Kingdom

^3^ Department of Evolutionary Genetics, Max Planck Institute for Evolutionary Anthropology, Leipzig, Germany

^4^ Division of Biochemistry, Department of Medical Biochemistry and Biophysics, Karolinska Institutet, Stockholm, Sweden

^5^ Department of Biosciences, Faculty of Sciences and Technology, Universitat de Vic - Universitat Central de Catalunya, Vic, Spain

^6^ Barcelona Supercomputing Center (BSC), Barcelona, Spain

^7^ Department of Health and Experimental Sciences, Universitat Pompeu Fabra, Barcelona, Spain

^8^ Institut de Recerca i Innovació en Ciències de la Vida i de la Salut a la Catalunya Central (IRIS-CC), Vic, Spain

^9^ Department of Selenoprotein Research, National Institute of Oncology, Budapest, Hungary

^10^ UCL Genomics, University College London, London, United Kingdom

^*^ These authors contributed equally.

#Corresponding authors: Jasmin Rees, Sergi Castellano

**Email:**  [jasmin.rees.18@ucl.ac.uk;](mailto:jasmin.rees.18@ucl.ac.uk;) [s.castellano@ucl.ac.uk](mailto:s.castellano@ucl.ac.uk)

**Materials and Methods**

**GPX6 and other GPX sequences**

The GPX6 coding sequences and proteins for 22 present-day mammal species were obtained from SelenoDB 2.0 (1) now available at [selenodb.crg.eu](http://selenodb.crg.eu/), and Ensembl (2), chosen from their availability and breadth across the mammalian tree. These mammals were: Chimpanzee (*Pan troglodytes*), Human (*Homo sapiens)*, Macaque (*Macaca mulatta)*, Marmoset (*Callithrix jacchus*), Bolivian squirrel monkey (*Saimiri boliviensis*), Tarsier (*Carlito syrichta*), Bushbaby (*Otolemur garnettii*), House mouse (*Mus musculus*), Rat (*Rattus norvegicus*), Chinese hamster (*Cricetulus griseus*) , Golden hamster (*Mesocricetus auratus*), Lesser Egyptian jerboa (*Jaculus jaculus*), Kangaroo rat (*Dipodomys ordii*), Squirrel (*Ictidomys tridecemlineatus*), Guinea pig (*Cavia porcellus*), Rabbit (*Oyctolagus cuniculus*), Walrus (*Odobenus rosmarus*), Cat (*Felis catus*), Horse (*Equus caballus*), Cow (*Bos taurus*), Pig (*Sus scrofa*) and Elephant (*Loxodonta africana*).

The Ensembl species tree (available at [www.ensembl.org](http://www.ensembl.org)) was used as the topology of the phylogeny of these mammals, with the exception of the walrus which was added according to various additional sources (3). These species include nine mammals where GPX6 contains Cys instead of Sec (Fig 1).

The orthologous GPX6 coding sequences and proteins were aligned using MAFFT (4). The posterior probability of each individual aligned position was then calculated using a modified version of HMMER (5). In short, each protein multiple alignment was first converted into a Hidden Markov Model before the use of a forward-backward algorithm (6) to perform posterior decoding. The calculated posterior probability integrates the uncertainty of the alignment around an aligned position, representing our degree of confidence in each individual aligned protein residue or gap in a multiple alignment.

Positions with an average posterior probability below 0.95 were removed from input to CODEML from PAML (7) owing to concerns of misalignment. The untrustworthy positions are generally found where gaps create alignment uncertainty or where sequence divergence contributes to alignment uncertainty. However, our probabilistic approach allowed us to keep those regions of the alignment with gaps and amino acid differences that are nevertheless confidently aligned in the multiple alignments.

The coding sequences for other members of the GPX family, where all species have Sec (GPX1, 2, 3 and 4) or Cys (GPX5, 7 and 8), were also obtained from SelenoDB 2.0 (1) or, if not available, from Ensembl (2) and aligned as described above.

**Inferring the Loss of Sec in GPX6**

The ancestral sequences of GPX6 for our set of 22 mammals were reconstructed using their present-day sequences and our phylogenetic tree of GPX6 (Figure 1) using the PAML package (7). The accuracy of ancestral node reconstruction per site is given in Fig S1, estimated to all be above 88.45%. The inferred ancestral nodes were then used to infer the independent losses of Sec throughout the mammalian lineage. The independent losses of Sec within the walrus and cat lineages were inferred according to the most parsimonious scenario when accounting for the presence of Sec in the Ursidae lineage (included as bear in Fig 1). The approximate ages of lineages with Sec loss are given as ranges in Fig 1, as collected from various sources describing split times in the mammalian phylogeny (3, 8–12).

Further to the PAML inference, the ancestral sequences were also inferred using two additional programs: Ancestor v1.1 (13) and FastML (14). FastML had the options of using either amino acid or nucleotide sequences of present-day species as input to infer the sequences, whereas Ancestor v1.1 (and PAML) only uses the nucleotide sequences for inference. Using both FastML input methods, this gave us four inferred sequences for all ancestral nodes. The four inferred sequences were then aligned using MAFFT (4) and the residue with most support was taken as the consensus residue for each site.

**dN/dS ratios in GPX proteins**

The dN/dS ratio was computed using the CODEML package from PAML (7), using the aligned GPX6 coding sequences and established tree topology (Fig 1) as input. This dN/dS is used as a quantification of the strength of selection acting on proteins, where dN is the rate of non-synonymous substitutions per non-synonymous sites and dS is the rate of synonymous substitutions per synonymous sites. Finally, the UGA codon encoding the Sec amino acid was considered an ambiguity character and, hence, not included in the dN/dS calculation. This makes our tests conservative when comparing the patterns of evolution in proteins that have lost Sec and gained Cys to those that have not.

Independent dN/dS ratios for each branch were estimated using the free-ratio model (model = 1) in PAML, which allows the dN/dS ratio to vary amongst the branches of the phylogenetic tree. This was used to compare the rate of evolution in the lineages that retain Sec and those that have exchanged Sec for Cys. Given this preliminary comparison, the CODEML branch model (model = 2) was then used to explicitly test our hypothesis of a faster rate of evolution in lineages where Sec was lost.

The CODEML branch model (model = 2) which allows us to specify the number of independent dN/dS ratios and on which branches they lie. We used this model to compare the dN/dS ratios between three groups of branches: the branches with Sec (Fig 1; solid red branches), the branches where Sec is exchanged for Cys (Fig 1; dashed green branches) and the branches where Cys is maintained (Fig 1; solid green branches). By using this comparison, we compare if the dN/dS ratio was significantly different in lineages at the time surrounding the loss of Sec compared to lineages where Sec was not lost, or where Cys was maintained and presumably any fitness reduced as a result of the loss of Sec had been recovered.

The branch models were compared to the null model (M0 model, model = 0) where singular dN/dS values are estimated for all branches. The likelihood of each of the two models were then compared to give a likelihood ratio, comparing if three dN/dS ratios across the tree is a better fit than the null model of a singular dN/dS ratio across all branches. The likelihood ratio was used to calculate the significance of the difference in fit between the two models in the form of a p-value (shown in Table 1). A significant deviation between the two models indicates a difference between the dN/dS ratios across the three groups of branches in the tree.

This analysis was repeated for other genes in the GPX family, comparing the rates of evolution of the lineages that have lost Sec in GPX6 and those that have not lost Sec or have maintained Cys (again with CODEML, branch model = 2) with the sequences of the Sec-containing GPX proteins (GPX1, 2, 3 and 4) and the Cys-containing proteins (GPX7, and 8). The likelihood ratio of the branch model to the null model was again calculated to give a P-value for significance (Table 1).

**dN/dS Ratios in the Protein Domains of GPX6**

To further explore the rate of evolution over the GPX6 protein, we separated the protein into three domains: N-terminus, GPX domain and C-terminus, as defined in the PFAM (15). Here, the GPX domain is essential for the catalytic activity of the enzyme with the C-terminus believed to also contribute to catalytic function (16). The dN/dS ratios for each domain were then compared between the Sec-containing lineages, the lineages where Sec was exchanged for Cys and the lineages where Cys was maintained of the GPX6 protein using CODEML, branch model = 2. The P-values for significance in the difference between the likelihood of the branch model to the null model for each domain are shown in Table 1. Calculations of dN/dS ratios over protein domains were repeated for the other genes in the GPX family (Table 1).

**dN/dS Ratios in GPX3 (sloth and kangaroo rat)**

We found that the Sec-containing GPX3 protein lacks its defining Sec residue in two species; the Hoffman’s two-toed sloth and the kangaroo rat. Here, the Sec has been exchanged for either Glutamine (in the case of the sloth) or for Serine (in the case of the kangaroo rat). Because of these exchanges, both the sloth-GPX3 protein and kangaroo rat-GPX3 protein were excluded from the branch model analysis of dN/dS in GPX3 (see “dN/dS ratios in GPX proteins”). However, these proteins were then included (and following alignment and HMMER analysis steps repeated) to allow Branch-site analysis on these two branches (see “Inferring Selection on the GPX6 Sites”; Table S1).

**Inferring Selection on the GPX6 Sites**

To test for selection acting on individual sites across the entire tree, we employed the Site model in PAML (Yang, 2007). We compared model 7 (beta; model = 0 , NSsites=7) to model 8 (beta plus selection; model =0, NSsites=8). Here, model 7 is the null model of a beta distributed variable selective pressure across sites, whereas model 8 is the beta distributed model plus selection.

Given the significance of this site model test, we then tested for selection acting on sites in the GPX domain along particular branches across the tree using the Branch-site model in PAML. This model (model = 2, NSsites=2) allows the dN/dS ratio to vary both amongst the sites and amongst the specified foreground and background branches and outputs the probability of each site being under selection in the foreground branches according to PAML’s Bayes Empirical Bayes (BEB) inference method (17).

This model classifies the sites into those that have dN/dS values that remain the same on the foreground and background branches ($\omega<1$ or $\omega=1$ in both branches) and those that differ amongst the branches ($\omega<1$ or $\omega=1$ in background branches and $\omega>1$in foreground branches), outputting the proportion of each site class. This method then calculates the posterior probability of each site being under selection in the foreground branches, whilst accounting for sampling errors by using a Bayesian prior (17). This model is compared to the corresponding null model, which is the same in all ways apart from the fixation of $\omega_{2}$.

We used this model initially on the branches where Sec was inferred to be lost (Fig 1; dashed green branches acting as the foreground) and found significant evidence for selection on particular sites within this region (Table S2). We then extended this model to test along the entire protein region for these same branches. However, given the non-significant result, we repeated the use of this model to the closest related lineages where Sec was inferred to be lost (see Table S2). This is because this model is suited to identifying a set of sites under selection across the entire set of foreground branches; if selection is acting across different sites in the foreground branches, as somewhat expected given the role of epistasis over more diverged lineages, this model will return a non-significant result. That is to say, we expect epistasis to place a limit the number of the same sites being under selection across more diverged lineages.

Given we see significant evidence for selection across the mostly closer related lineages where Sec was inferred to be lost (the branch leading to squirrel monkey-marmoset, the Eumuroida branch and the branch leading to rabbit; see Fig 1), we are able to test if these probabilities are enriched in certain subsets of sites using Mann-Whitney U tests (see “Identifying Convergent Changes across Cys-Branches” and “Convergence in the *Eumuroida* Lineage”).

**Identifying Convergent Changes across Cys-Branches**

Convergent changes in GPX6 across lineages were identified using CONVERG2 (18), which uses the alignments of modern proteins and an input phylogeny to identify sites between a pair of lineages that have changed from the common ancestor. This programme differentiates between parallel and convergent amino acid changes (parallel changes being those changes from the same ancestral amino acid to the same derived amino acid, convergent changes being those changes from a different ancestral amino acid to the same derived amino acid) but for brevity, both are referred to as convergent amino acid changes here.

Convergent changes were identified between the GPX6_Cys_ lineages; either the branches where Sec was exchanged for Cys or the species branches where Cys was maintained (the dashed and solid green lines in Fig 1, respectively). The observed frequency of these convergent changes was then compared with the expected frequency of convergent changes, also calculated using CONVERG2.

Since the pathway to recover catalytic activity may not be limited to the same amino acid changes, but may be restricted to particular sites in the protein, the CONVERG2 programme was also edited to identify convergent site changes, which do not condition on resulting in the same amino acid across branches. Hence, convergent site changes are used to identify sites that show repeated changes across lineages. Both convergent amino acid and site changes are referred to as convergent in the paper, but the exact nature of each convergent change can be seen in Tables S3-9.

Where the sequences for the species containing Cys in GPX6 were available, the equivalent analyses were run on the Sec-containing GPX proteins (GPX1, 2, 3 and 4) and the Cys-containing proteins (GPX7 and 8). We advise focusing on the CONVERG2 results for GPX3 and GPX5 for two reasons: 1) these are the immediate paralogues to GPX6; and 2) the gaps in the other proteins do not allow, we believe, a full representation of the potential instances of convergence. Still, we do stress that the pattern over all of these non-GPX6 proteins is that of much reduced convergence, if any, across the Cys-lineages identified in GPX6.

Using the posterior probabilities of selection obtained from the BEB results of the Branch-site model when the foreground branches are specified as the branch leading to squirrel monkey-marmoset, the Eumuroida branch and the branch leading to rabbit (*i.e.,* using the probabilities of sites being under selection in these three branches), we also compared the posterior probability of selection on convergent sites. Here, we exclude convergent sites only identified using either the cat or walrus terminal branches, since they are excluded from the probability calculation. To test for enrichment, we use a Mann-Whitney U test to account for the non-parametric data.

**Convergence in the *Eumuroida* Lineage**

By inferring the GPX6 protein sequences along the *Eumuroida* branch (see “Inferring the Loss of Sec in GPX6”) we identified 26 sites that changed over the *Eumuroida* branch. Using the results from CONVERG2, we were then able to identify 15 sites that show signatures of convergence across GPX6_Cys_ lineages (to the exclusion of those identified from cat and walrus) and show the distribution within each protein domain. We were also able to infer a further 22 amino acid sites (19 substitutions and a 3 C-terminal extension) that changed between the end of the *Eumuroida* branch (dashed green branch in Figure 2a) and the modern mouse GPX6 protein; m-GPX6_Cys+22_. CONVERG2 was again used to identify which of these 19 substitutions signatures of convergence across GPX6_Cys_ lineages.

These subsets of sites, both the 15 along the *Eumuroida* branch and the 8 leading to modern day mouse, were tested for enrichment of selection signatures by comparing the posterior probabilities of selection using a Mann-Whitney U test. Here, we again use these posterior probabilities are the probabilities of sites being under selection in the branch leading to squirrel monkey-marmoset, the Eumuroida branch and the branch leading to rabbit.

Using PHYML (19), we reconstructed the mammalian tree given: a) the full GPX6 gene, b) the N-terminal of GPX6, c) the GPX domain of GPX6, d) the N-terminal of GPX6, e) the 26 sites that change across the *Eumuroida* branch (Fig S4) as well as the 14 sites that show changes across the *Eumuroida* branch and convergent changes across $GPx6_{Cys}$ branches (Fig 2b). This was repeated using the full GPX3 and GPX5 proteins (Fig S5). We also reconstructed mammalian trees using GARD, which infers different rates of evolution (presumed to be recombination breakpoints) along a sequence to output partition trees, separated by inferred breakpoints. GARD (Pond et al, 2006) infers three different phylogenies, recapitulating what has previously been shown: phylogenies are distorted across different sections of the protein (likely due to molecular convergence as discussed in the manuscript) and Cys-containing lineages show faster rates of evolution (Fig S6).

**Expected Levels of Convergence given rate of amino acid exchange**

The evolution of the GPX6 protein sequence across our mammalian phylogeny was simulated using Seq-Gen (20). This simulated evolution begins with the inferred ancestral sequence at the base of our mammalian clade (see “Inferring the Loss of Sec in GPX6”) and extends to all modern mammalian proteins, using the JTT model of amino acid substitution. Tree lengths were given by the rate of amino acid changes along each branch of the mammalian tree as from the calculated dN value in the CODEML package from PAML ((7); see “dN/dS ratios in GPX proteins“). Hence, this simulation recreates chance amino acid exchanges along each branch at its observed rate.

Having run the simulation 1,000 times, we then use CONVERG2 (18) to identify convergent site changes between the lineages where Sec was lost for Cys (solid green lines in Fig 1) for each simulation run (see “Identifying Convergent Changes across Cys-Branches”). The distribution of convergent changes under this expected rate of amino acid exchange is then plotted, and compared to the observed number of convergent site changes (Fig S7). The equivalent simulations were run for all other GPX proteins, and we further compared the observed and expected number of convergent site changes for these proteins (Figs S7-13)

To confirm that the higher number of observed convergent changes relative to our expectation are focused within the functional GPX domain, and this isn’t simply an artefact of elevated evolutionary rate within this domain, we also repeated these simulations on only the GPX domain. Here, the tree lengths were given by the rate of amino acid changes (dN) from the GPX domain only. Comparisons between observed and expected convergent site changes are given in Fig S8.

**Ancestral Reconstruction of GPX6 along the *Eumuroida* Lineage**

Following our inference of ancestral sequences (see “Inferring the Loss of Sec in GPX6”), we were able to reconstruct 3 ancient proteins along the *Eumuroida* lineage. These proteins are: 1) the protein just prior to the loss of Sec in the ancestor of *Eumuroida* (Eu- GPX6_Sec_); 2) the same ancestral protein but with Sec exchanged for Cys (Eu- GPX6_Cys_); 3) the protein at the derived end of the *Eumuroida* branch, now containing the additional 25 sites that have changed along the *Eumuroida* branch (Eu- GPX6_Cys+25_).

As previously described (see “Inferring the Loss of Sec in GPX6”), the residue with most support from the four inferred sequences was taken as the consensus residue for each site, with the exception of site 54 in Eu- GPX6_Cys+25_. Here, the consensus residue was taken as “Q” despite the methods used suggesting “H” since “H” is not present at that site for any of the present-day species. Of the 217 amino acid sites, 208 (95.85%) were resolved unanimously across the four inference methods. Of the remaining 9 sites (4.15%) that were inferred differently across them methods, 7 (3.23% of total sites) of these sites differed across the inference of the Eu- GPX6_Sec_ protein and 2 (0.92% of total sites) differed across the inference of the Eu- GPX6_Cys+25_.

These consensus sequences provide the final Eu- GPX6_Sec_ and Eu- GPX6_Cys+25_ proteins. As before, sites with an average posterior probability below 0.9, as calculated using HMMER (5) were removed from subsequent PAML analysis.

**Experimental Reconstruction and Enzymatic Assay of Ancient GPX6 Proteins**

The sequences of the proteins used in the experimental assessment, three ancestrally reconstructed from along the Eumuroida lineage (Eu-GPX6_Sec_, Eu-GPX6_Cys_ and Eu-GPX6_Cys+25_ ), one modern mouse protein (m-GPX6_Cys+22_) and two synthetic modern mouse proteins (m-GPX6_Sec+22_ and m-GPX6_Ser+22_ ) are given below (Sec, Cys or Ser highlighted in yellow). Given that the full Eu-GPX6_Cys+25_ (218) was insoluble, we removed the potential signal peptide to result in a now soluble protein (199 residues).

Eu-GPX6_Sec_

MGSSHHHHHHGTMSDSEVNQEAKPEVKPEVKPETHINLKVSDGSSEIFFKIKKTTPLRRLMEAFAKRQGKEMDSLRFLYDGIRIQADQTPEDLDMEDNDIIEAHREQIGGMVQQFWASCLFPLFLAGLAQETLKPQKMKVDCNKGVTGTIYEYGALTLNGEEYIQFKQYAGKHVLFVNVATYUGLTAQYPELNALQEELKHFDVIVLGFPCNQFGKQEPGKNSEILSGLKYVRPGGGFVPNFQLFEKGDVNGEKEQKVFTFLKNSCPPTSELLGSPKQLFWEPMKVHDIRWNFEKFLVGPDGVPVMRWFHQTPVSTVKSDILEYLKQS-

Eu-GPX6_Cys_

MGSSHHHHHHGTMSDSEVNQEAKPEVKPEVKPETHINLKVSDGSSEIFFKIKKTTPLRRLMEAFAKRQGKEMDSLRFLYDGIRIQADQTPEDLDMEDNDIIEAHREQIGGMVQQFWASCLFPLFLAGLAQETLKPQKMKVDCNKGVTGTIYEYGALTLNGEEYIQFKQYAGKHVLFVNVATYCGLTAQYPELNALQEELKHFDVIVLGFPCNQFGKQEPGKNSEILSGLKYVRPGGGFVPNFQLFEKGDVNGEKEQKVFTFLKNSCPPTSELLGSPKQLFWEPMKVHDIRWNFEKFLVGPDGVPVMRWFHQTPVSTVKSDILEYLKQS-

Eu-GPX6_Cys+25_

MGSSHHHHHHGTMSDSEVNQEAKPEVKPEVKPETHINLKVSDGSSEIFFKIKKTTPLRRLMEAFAKRQGKEMDSLRFLYDGIRIQADQTPEDLDMEDNDIIEAHREQIGGQKAKVDCNKGVTGTIYEYGANTLNGGEYVQFQQYAGKHILFVNVASFCGLTATYPELNTLQEELKQFNVIVLGFPCNQFGKQEPGKNSEILLGLKYVRPGGGYVPNFQLFEKGDVNGENEQKVFSFLKNSCPPTSELLGSPEHLFWDPMKVHDIRWNFEKFLVGPDGVPVMRWFHQTPVSTVQSDIMEYLNQS-

m-GPX6_Cys+22_

MGSSHHHHHHGTMSDSEVNQEAKPEVKPEVKPETHINLKVSDGSSEIFFKIKKTTPLRRLMEAFAKRQGKEMDSLRFLYDGIRIQADQTPEDLDMEDNDIIEAHREQIGQETLNPQKSKVDCNKGVTGTVYEYGANTIDGGEFVNFQQYAGKHILFVNVASFCGLTATYPELNTLQEELKPFNVTVLGFPCNQFGKQEPGKNSEILLGLKYVRPGGGYVPNFQLFEKGDVNGDNEQKVFSFLKNSCPPTSELFGSPEHLFWDPMKVHDIRWNFEKFLVGPDGVPVMRWFHHTPVRIVQSDIMEYLNQTSTQ-

m-GPX6_Sec+22_ MGSSHHHHHHGTMSDSEVNQEAKPEVKPEVKPETHINLKVSDGSSEIFFKIKKTTPLRRLMEAFAKRQGKEMDSLRFLYDGIRIQADQTPEDLDMEDNDIIEAHREQIGQETLNPQKSKVDCNKGVTGTVYEYGANTIDGGEFVNFQQYAGKHILFVNVASFUGLTATYPELNTLQEELKPFNVTVLGFPCNQFGKQEPGKNSEILLGLKYVRPGGGYVPNFQLFEKGDVNGDNEQKVFSFLKNSCPPTSELFGSPEHLFWDPMKVHDIRWNFEKFLVGPDGVPVMRWFHHTPVRIVQSDIMEYLNQTSTQ-

m-GPX6_Ser+22_

MGSSHHHHHHGTMSDSEVNQEAKPEVKPEVKPETHINLKVSDGSSEIFFKIKKTTPLRRLMEAFAKRQGKEMDSLRFLYDGIRIQADQTPEDLDMEDNDIIEAHREQIGQETLNPQKSKVDCNKGVTGTVYEYGANTIDGGEFVNFQQYAGKHILFVNVASFSGLTATYPELNTLQEELKPFNVTVLGFPCNQFGKQEPGKNSEILLGLKYVRPGGGYVPNFQLFEKGDVNGDNEQKVFSFLKNSCPPTSELFGSPEHLFWDPMKVHDIRWNFEKFLVGPDGVPVMRWFHHTPVRIVQSDIMEYLNQTSTQ-

These proteins were produced as recombinant proteins heterologously expressed in *Escherichia coli*. Sec can be efficiently incorporated at a predefined UAG codon and by using a mutant *E.coli* strain that does not recognise UAG as a STOP codon (strain C321.ΔA; all 321 endogenous UAG stop codons are replaced by UAA and its UAG-specific release factor 1 (RF1) deleted). We achieve a much higher yield of Sec-containing enzymes that would otherwise be produced by *E.coli* with standard genetic code decoding (21). Typically, approximately 20% Sec contents are reached in these recombinant enzymes (21).

The open reading frames for Eu-GPX6_Sec_ and m-GPX6_Sec+22,_ codon optimized and in fusion with a His-tagged SUMO domain for recombinant expression in *E. coli*, were synthesized and delivered in the form of a plasmid, respectively, by Integrated DNA Technologies, Inc. The corresponding ORFs of Eu-GPX6Sec and m-GPX6_Sec+22_ were subcloned into the in-house developed pABC2a plasmid for selenoprotein production (21), and the resulting two plasmids were subsequently transformed into release factor 1 (RF1) depleted *E.coli* strain C321.∆A for production of the fusion proteins of His6-SUMO-Eu-GPX6_Sec_, His6-SUMO-MmGPX6-C73U, respectively.

The open reading frames for the remaining proteins expressed and purified in this study were subcloned into another in-house developed pD441a plasmid for common protein production, and subsequently transformed into BL21(DE3) *E.coli* strain for production of the fusion proteins of His6-SUMO-Eu-GPX6_Cys_, His6-SUMO- m-GPX6_Cys+22_, and His6-SUMO- m-GPX6_Ser+22_ .

Recombinant selenoproteins were purified as also described in (21). Briefly, 40 mL of overnight cultures of transformed bacteria were inoculated into 2 liter terrific broth (TB) medium containing 50 µg/mL streptomycin and 50 µg/mL carbenicillin in a 5 liter bottle placed on a shaking incubator at 30 °C. At 6 hours after inoculation, temperature was lowered to 25 °C and 0.5 mM IPTG together with 5 µM sodium selenite were added to induce selenoprotein expression overnight. The bacteria were subsequently harvested by centrifugation, suspended in IMAC binding buffer (50 mM Tris-HCl, 100 mM NaCl, 10 mM imidazole, pH 7.5) and lysed by sonication. The soluble fraction was recovered by centrifugation and applied onto a HisPrep FF 16/10 column equipped on an ÄKTA explorer FPLC system (Cytiva Life Sciences). The eluted fusion protein was treated with inhouse produced His-tagged ULP1 (1%) and subsequently re-applied onto the HisPrep FF 16/10 column to separate non-tagged target protein from its N-terminal His-tagged fusion partner as well as from the His-tagged ULP1. The target proteins were then concentrated, buffer exchanged, and stored in -20°C freezer until analyses. The buffer for all final preparation is 50 mM Tris-HCl, pH 7.5, with 100 mM NaCl, and 20% glycerol. The purity of the final selenoprotein was always greater than 90% as assessed by SDS-PAGE. The procedure for expression and purification of all non-seleno-proteins were similar to that of the selenoproteins, except that the culture medium contained 50 µg/mL kanamycin, culture temperature before IPTG induction was 37 °C, and no additional selenite was added.

GPX activity was of Eu-GPX6_Sec_, Eu-GPX6_Cys_ and Eu-GPX6_Cys+25_ proteins was measured using GSH with either H_2_O_2_ or cumene hydroperoxide (CHP) as substrates in assays coupled with glutathione reductase (GR) and NADPH. The reactions were carried out in either 96-well plates or 1-cm cuvettes containing the indicated concentrations of GPX isoenzymes together with 1 mM GSH, 0.5 mM H2O2 or CHP, 15 nM human GR, and 0.2 mM NADPH. The resulting NADPH consumption was monitored by determining the linear decrease over time in the NADPH absorbance at a wavelength of 340 nm, always using two background controls, containing all reaction components except GPX isoenzyme or addition of H_2_O_2_, respectively. We utilized two different peroxide substrates to assess the potential GPX-like activities of the modern mouse and its mutant enzymes, i.e. running coupled assays with either hydrogen peroxide (H_2_O_2_) or cumene hydroperoxide (COOH). Both the wild type m-GPX6_Cys+22_ enzyme and its m-GPX6_Ser+22_ mutant were completely devoid of detectable peroxidase activity in these assays, while the m-GPX6_Sec+22_ mutant displayed a strong and typical GPx activity (Fig S16).

**Molecular docking simulations**

Structures for the GPX6 orthologs and nodes of the ancestral sequence reconstructions were built using AlphaFold2 (22). All protein sequences considered cysteines at their catalytic positions, given the inability to represent non-canonical residues for the *ab initio* model construction. We ran protein-ligand binding energy landscape explorations using the PELE software (23) for each protein structure. Ligands for the simulation were glutathione and glutathione disulfide. Simulations were first run to discover catalytic poses with low global energies; the catalytic distance was considered as the closest sulphur-sulphur distance between the catalytic cysteine and the glutathione sulphur atoms. The lowest binding energy poses, filtered by a catalytic distance threshold below 4Å, were used to run a second PELE simulation, thus focusing on exploring this catalytic minimum binding energy configuration. Each simulation comprised 95 replicas of 100 equilibration steps that constrained the ligand to its starting position, followed by 1000 PELE steps without any constraint over the ligand coordinates.

All simulation trajectories for the same ligand were simultaneously analyzed using as features all ligand positions aligned to a common protein reference structure. A Time-structure Independent Component Analysis (TICA) was built to find the common slowest-relaxing feature combination (24) with the PyEMMA library (25). Finally, and separately for each protein and ligand simulation, the probabilities of visiting the slowest TICA coordinate (IC1) according to the catalytic distance (S-S) were plotted as a free energy map (Fig 4 and Fig S17).


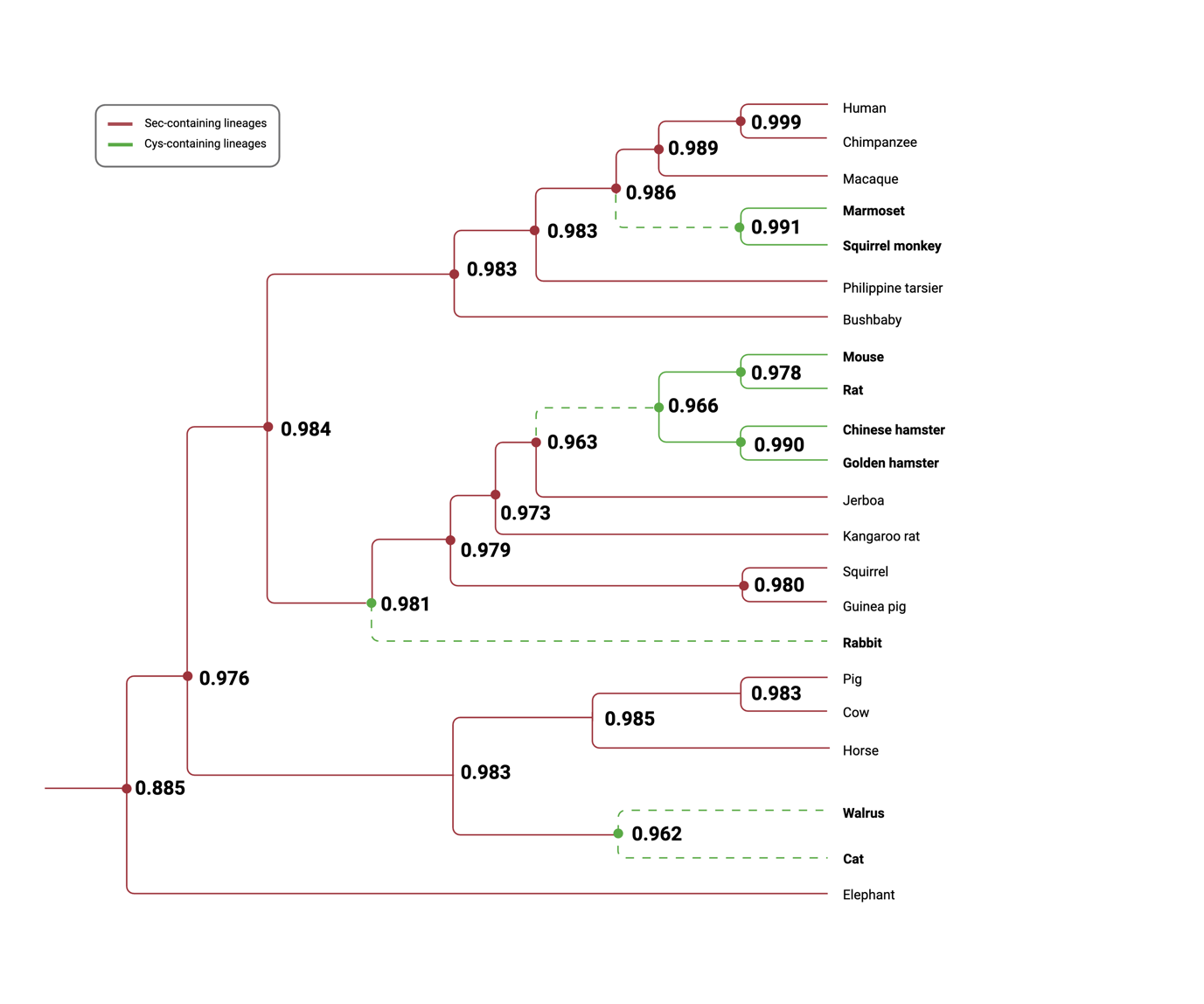
**Fig. S1.** The accuracy of the sequence reconstruction for each ancestral node in our mammalian phylogeny, as reconstructed using the PAML package (7). This is given as accuracy per site.

**
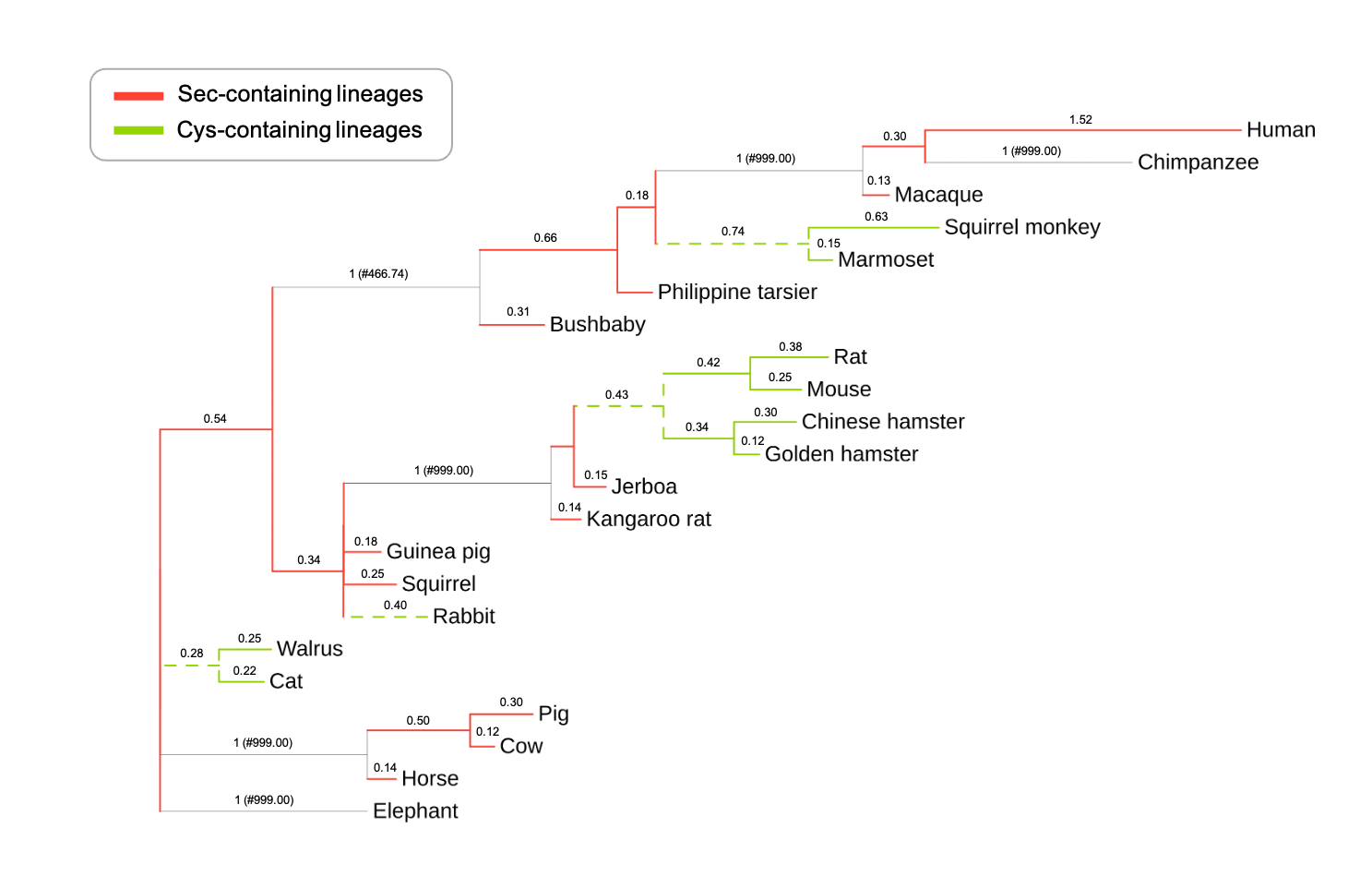
**

**Fig. S2.** Phylogeny of the 22 mammals in our analysis. In red, GPX6_Sec_ branches, in green, GPX6_Cys_ ones. Branch lengths are proportional to their corresponding dN/dS as estimated by the free-ratio model in PAML (*27*). In some lineages, PAML estimates very few synonymous changes compared to non-synonymous changes and this results in an unnaturally large dN/dS value. These branches are assigned a dN/dS ratio of 1 and coloured grey, with the actual ratio estimated by PAML is given in parenthesis (#). Branches with dN/dS values given as less than 0.01 are not labelled.

**
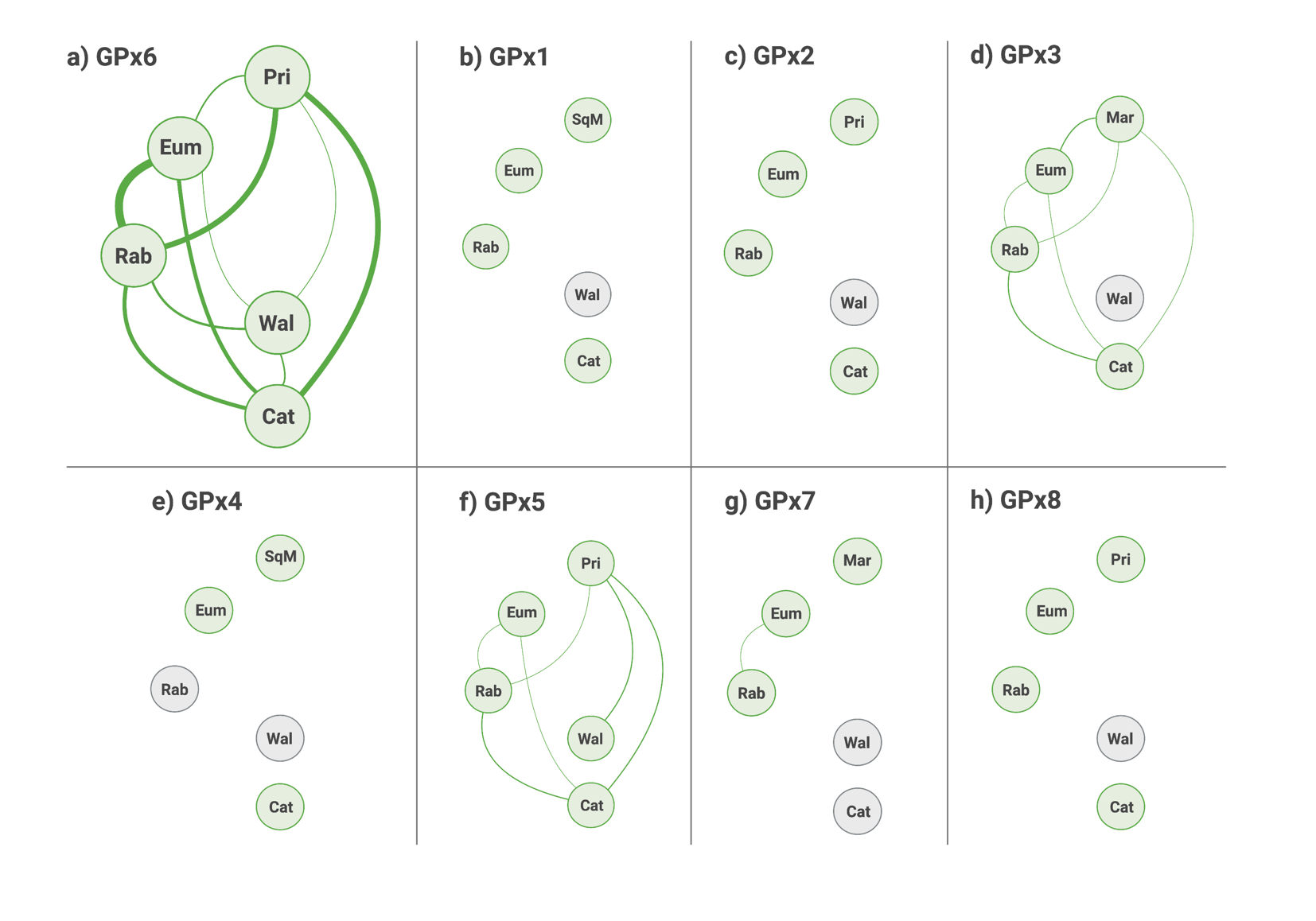
**

**Fig. S3.** Schematic diagram demonstrating the convergence between branches in GPX6 where Sec was inferred to have been lost. Connection thickness is proportional to the number of convergent sites identified. When a species protein is unavailable, the node is in grey. Pri = primate branch (leading to squirrel monkey and marmoset; Fig 1); Eum= Eumuroida; Rab = Rabbit; Wal = Walrus; Cat = Cat; SqM = Squirrel monkey; Mar = Marmoset

**
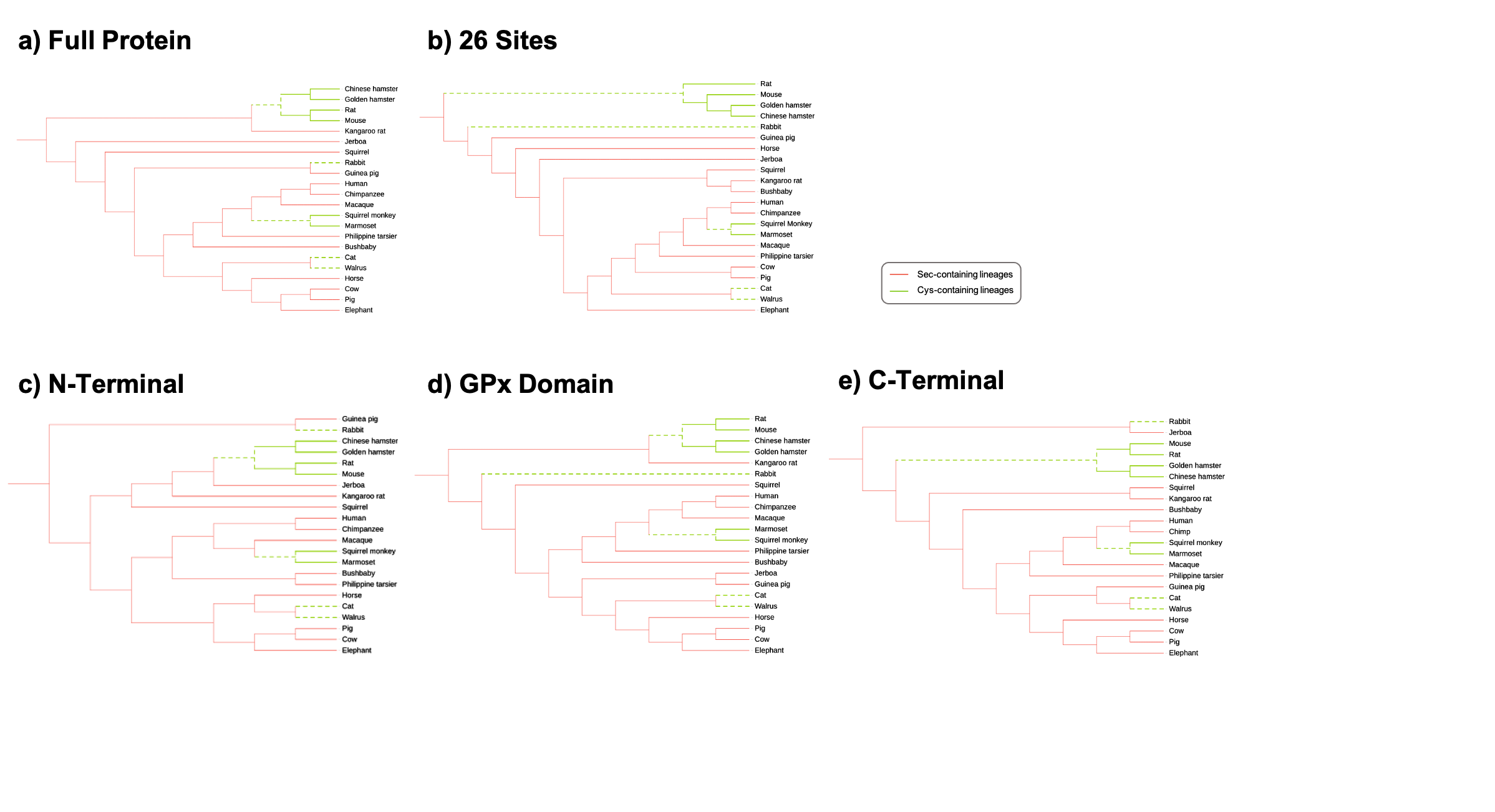
**

**Fig. S4.** Topology of the phylogenetic tree for GPX6, with midpoint rooting, constructed using PHYML (*41*) from the **a**) full GPX6 protein; **b**) the 26 sites that differ between Eu-GPX6_Sec_ and Eu-GPX6_Cys+25_; **c**) the N-terminal of GPX6; **d**) the GPX domain of GPX6 and **e**) the C-terminal of GPX6. In red, GPX6_Sec_ branches, in green, GPX6_Cys_ ones. Dashed green branches represent GPX6_Cys_ lineages at the time Sec was lost.

**
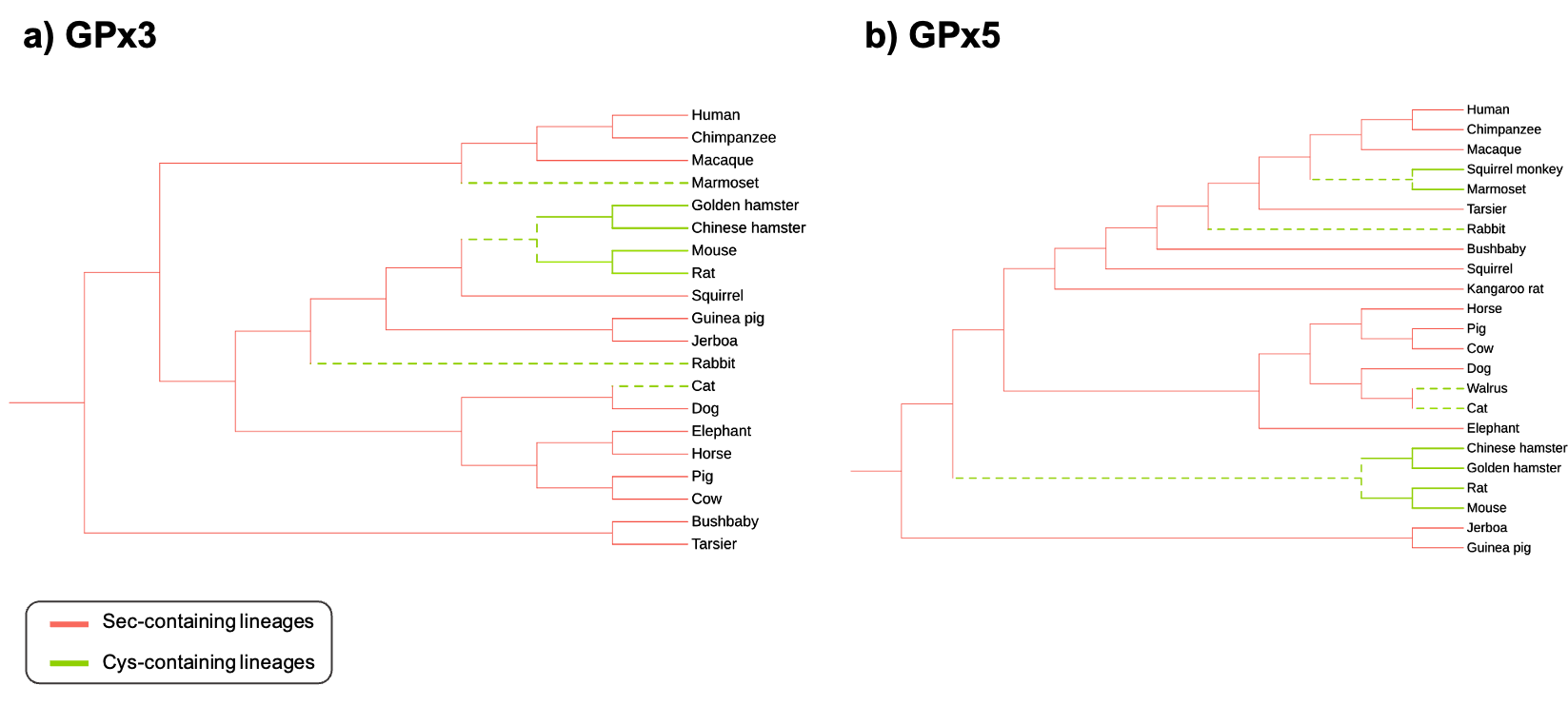
**

**Fig. S5.** Topology of the phylogenetic trees, with midpoint rooting, constructed using PHYML (*41*) from the available mammalian proteins of a) GPX3 and b) GPX5. In green, GPX6_Cys_ ones. Dashed green branches represent GPX6_Cys_ lineages at the time Sec was lost. The species names in italics indicate those species not represented in our wider GPX6 analysis.


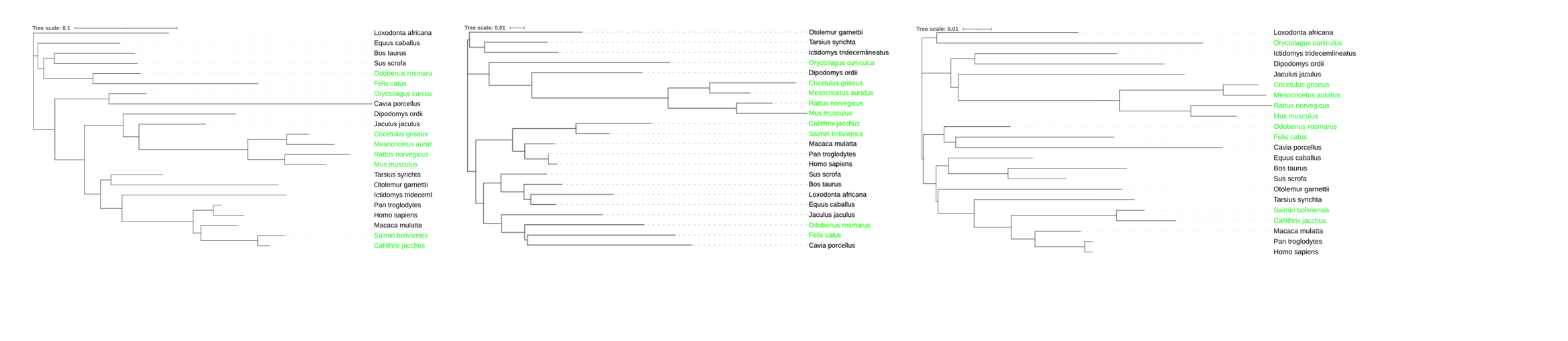


**Fig. S6.** Topology of the paritition trees, as inferred by GARD (Pond et al, 2006), constructed using PHYML (*41*) from the available mammalian proteins of GPX6. From left to right, the trees are constructed according to bases 1-108; 109-279; 280-648.


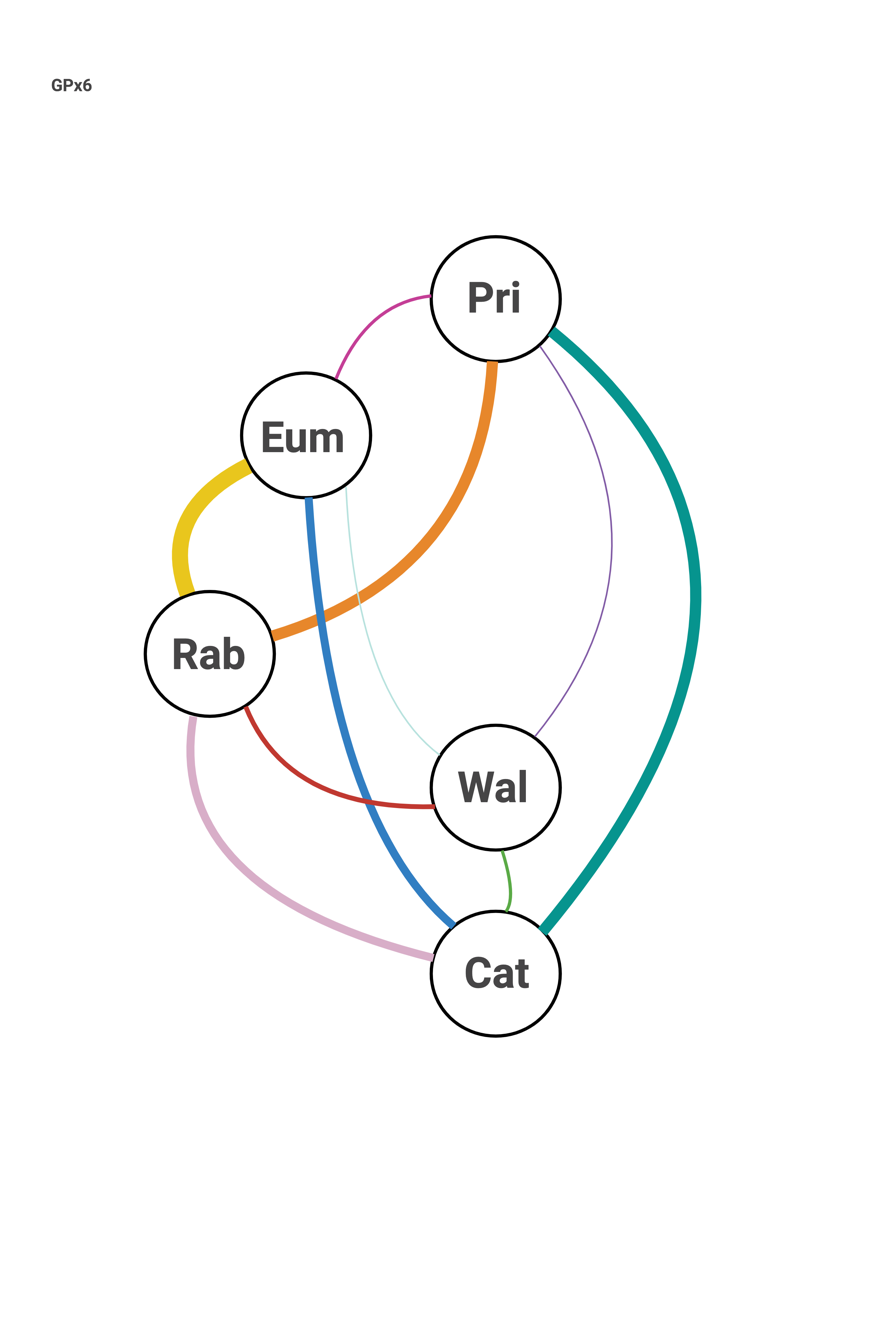

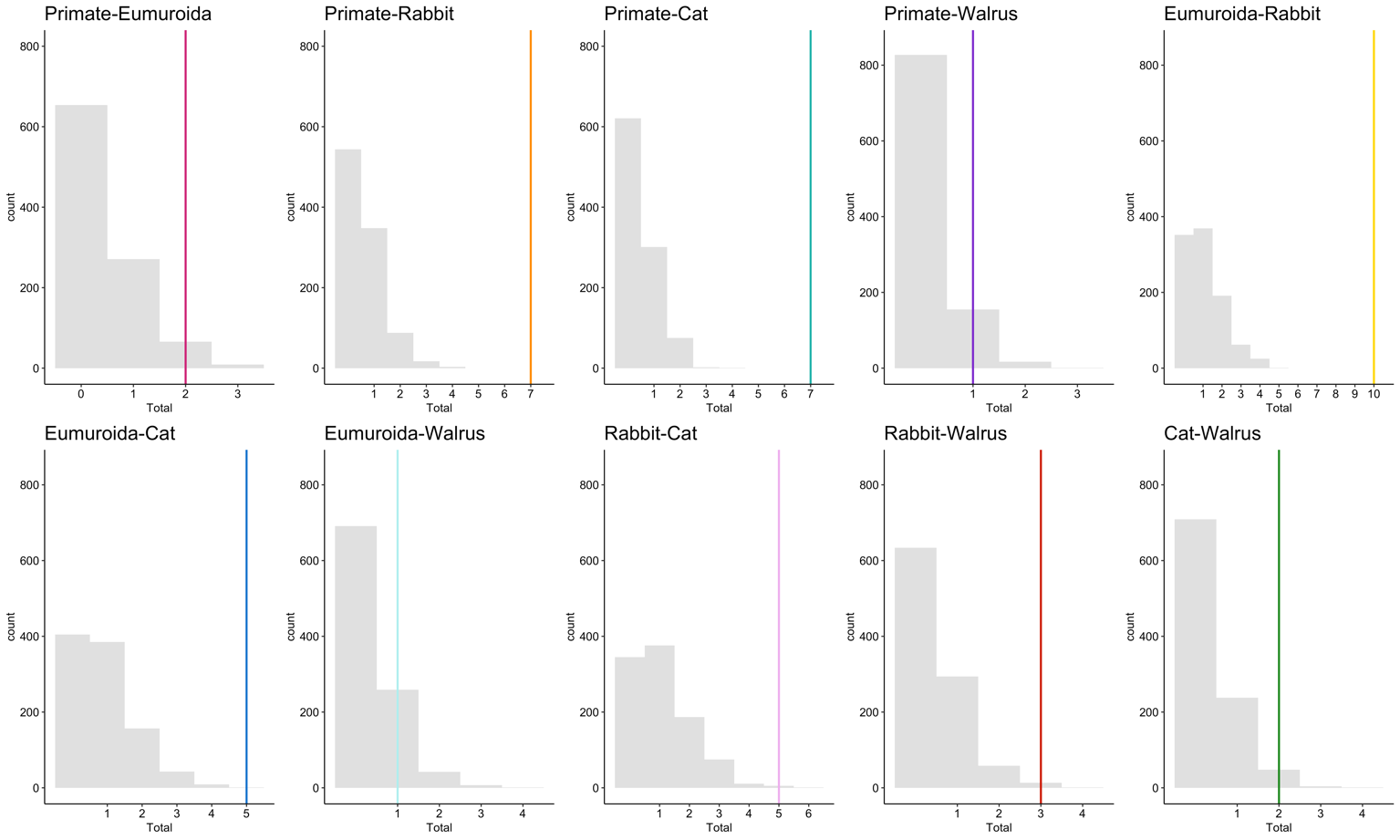


**Fig. S7.** Schematic representation of the number of observed convergence in the GPX6 protein between lineages where Sec is lost for Cys, where thickness of the line represents the number of convergent changes (left). Expected distribution of convergent changes in the full GPX6 protein between lineages where Sec is lost for Cys according to our Seq-Gen simulations, where the observed numbers of convergent changes are given by coloured lines (right).


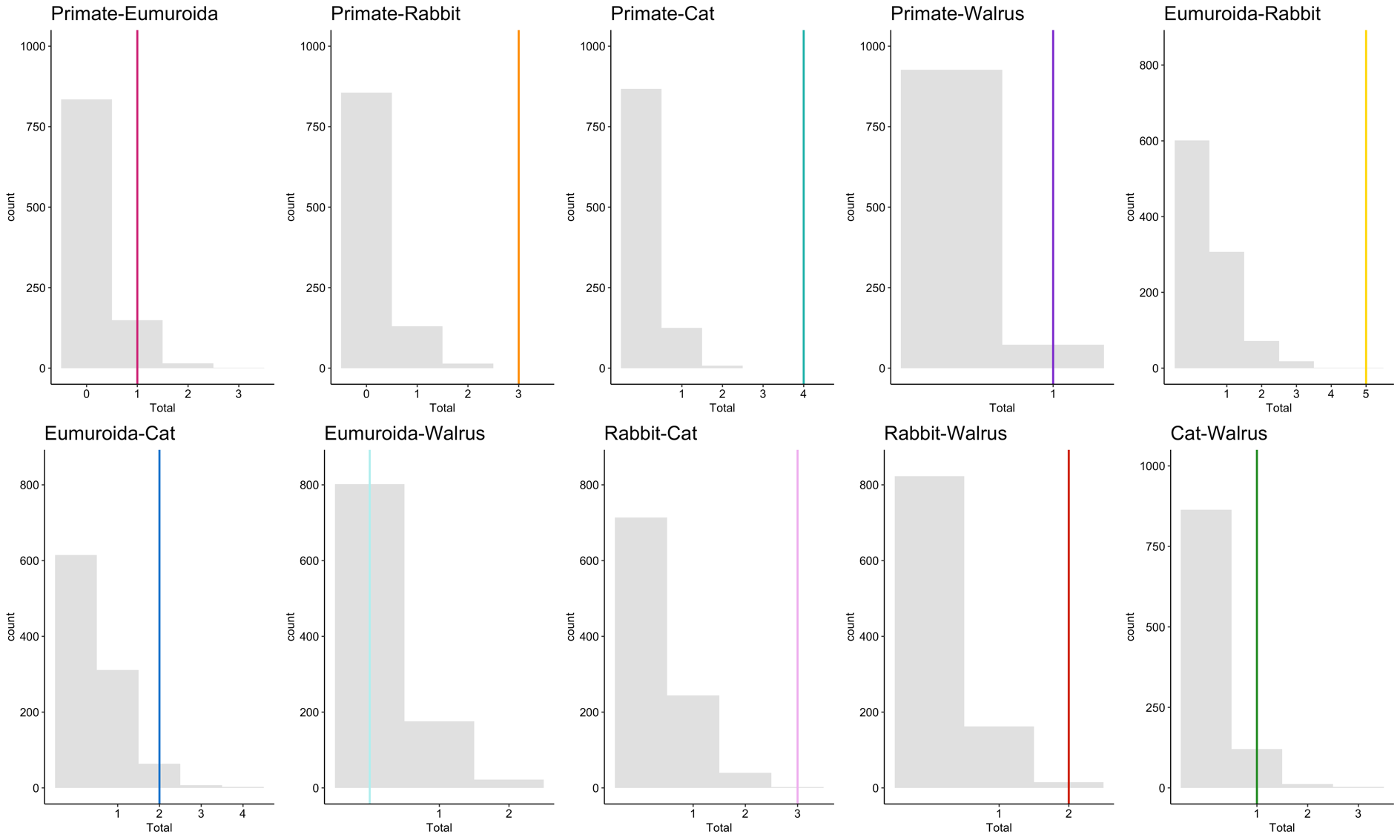


**Fig. S8.** Expected distribution of convergent changes in the GPX domain of the GPX6 between lineages where Sec is lost for Cys according to our Seq-Gen simulations, where the observed numbers of convergent changes are given by coloured lines.


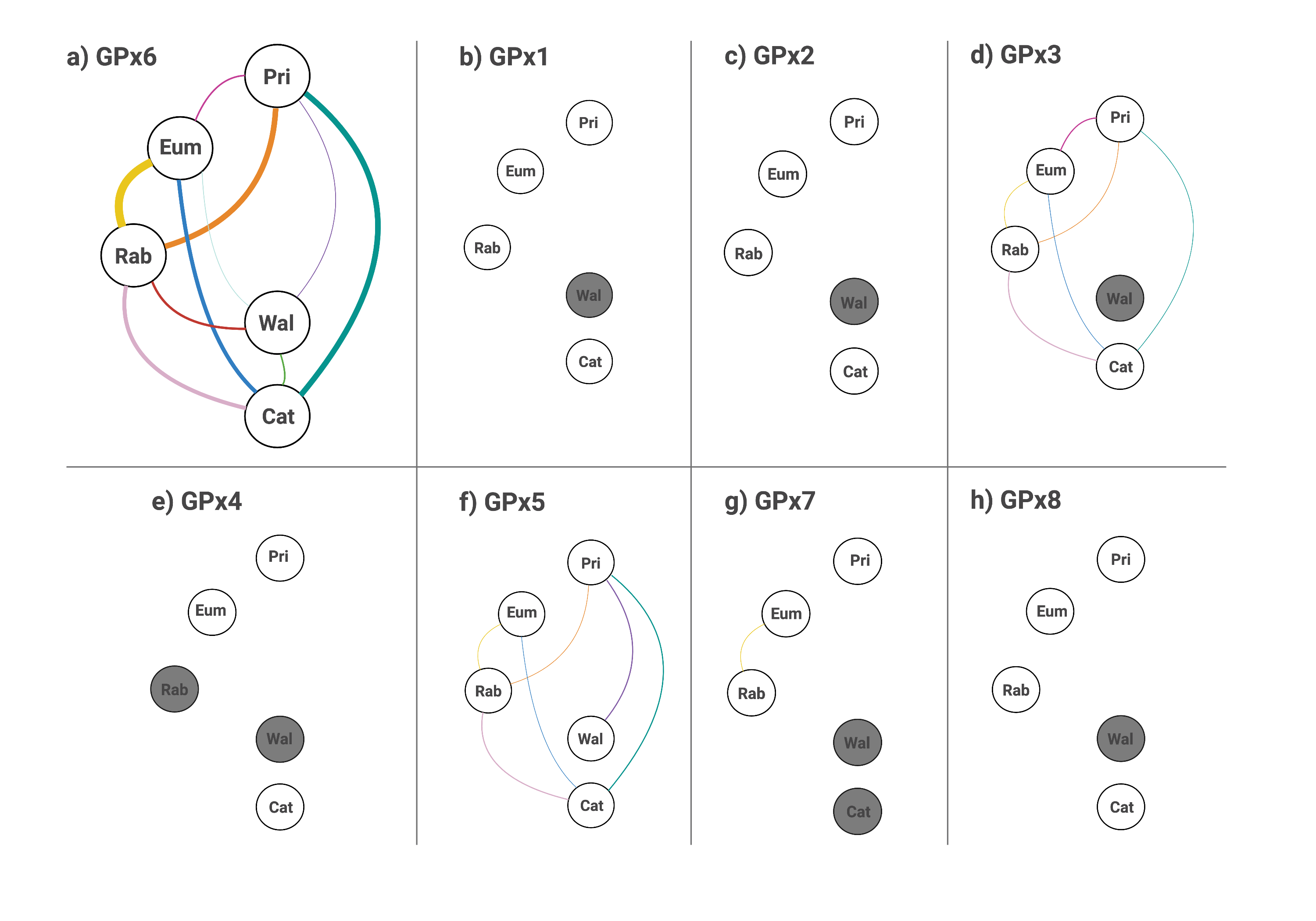

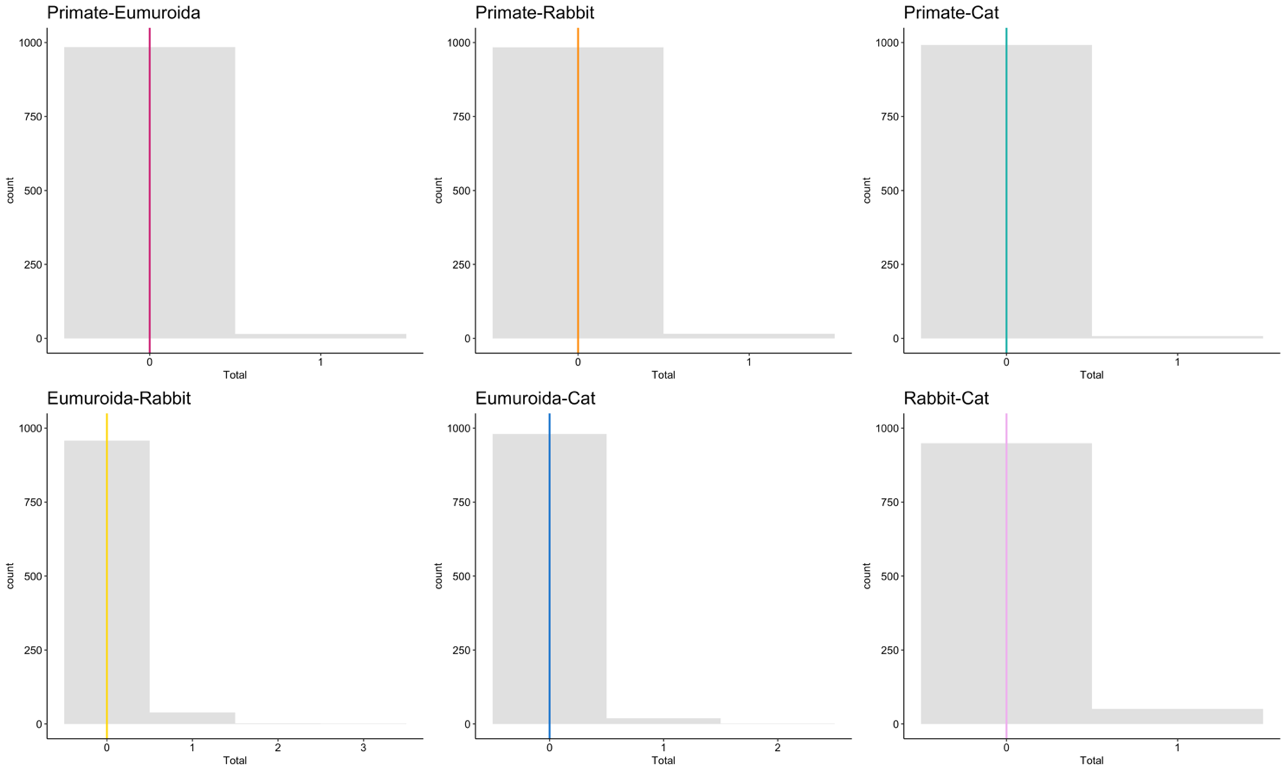


**Fig. S9.** Schematic representation of the number of observed convergence in the GPX1 protein between lineages where Sec is lost for Cys in GPX6, where thickness of the line represents the number of convergent changes (left). Expected distribution of convergent changes in GPX1 between lineages where Sec is lost for Cys in GPX6 according to our Seq-Gen simulations, where the observed numbers of convergent changes are given by coloured lines (right).

**
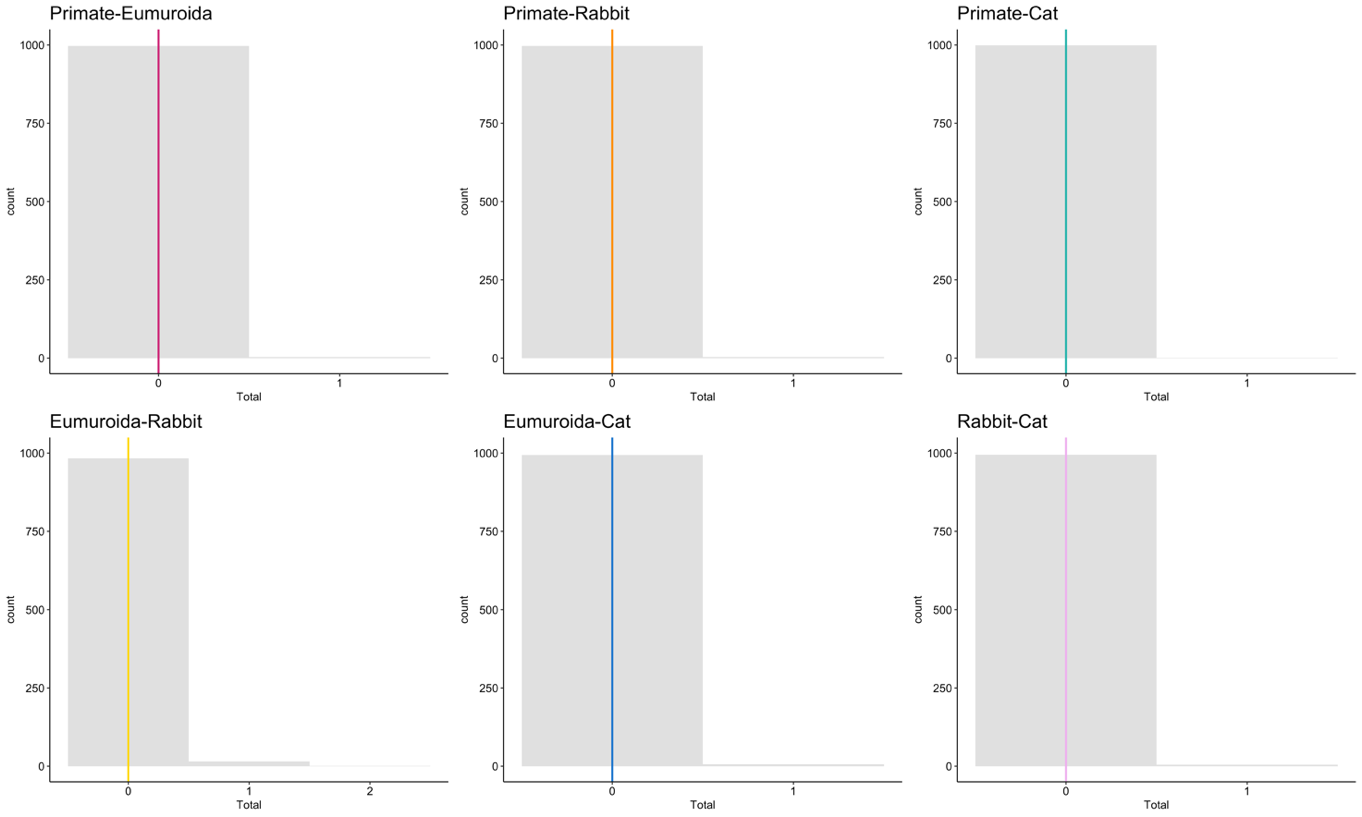
**
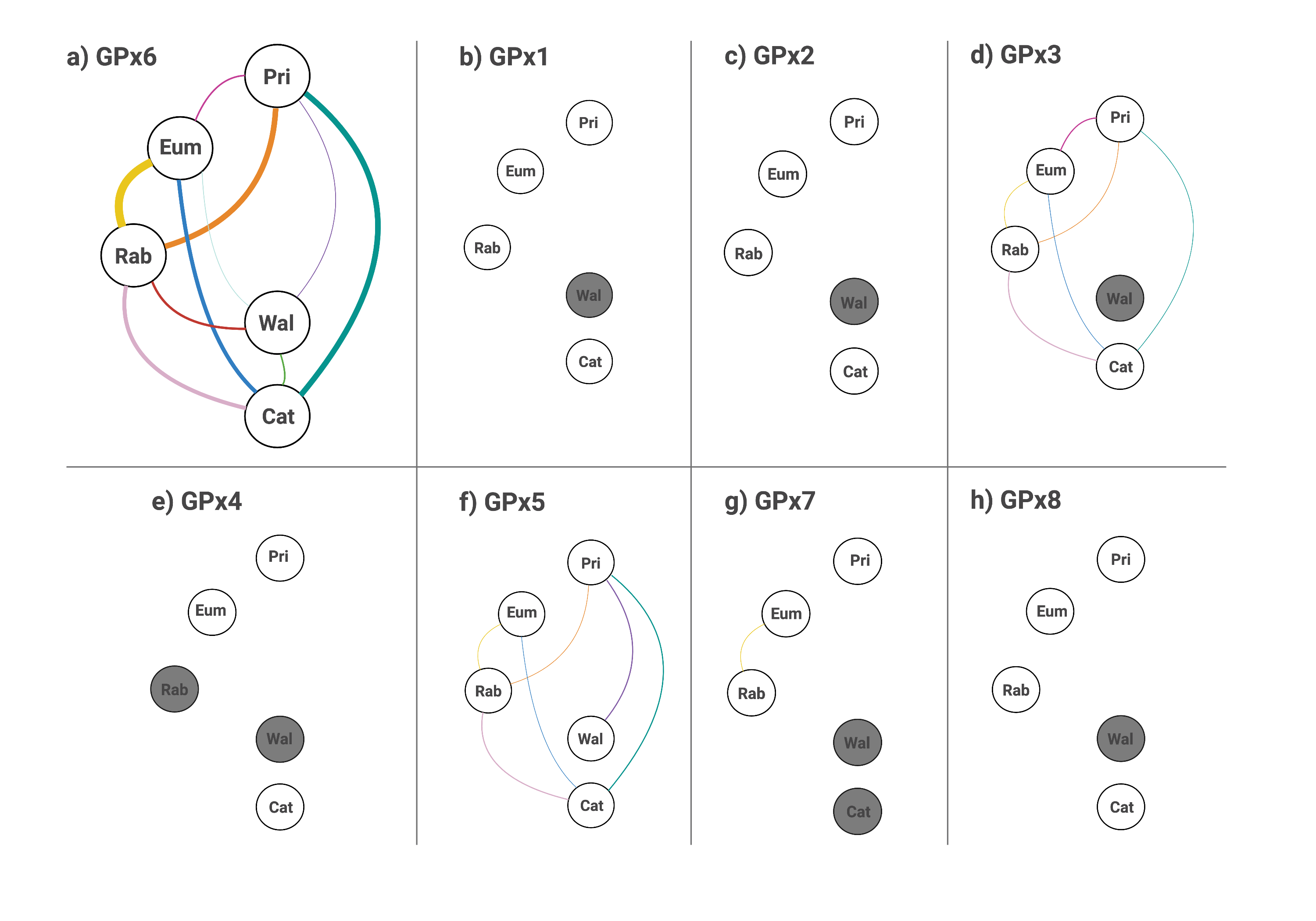


**Fig. S10.** Schematic representation of the number of observed convergence in the GPX2 protein between lineages where Sec is lost for Cys in GPX6, where thickness of the line represents the number of convergent changes (left). Expected distribution of convergent changes in GPX2 between lineages where Sec is lost for Cys in GPX6 according to our Seq-Gen simulations, where the observed numbers of convergent changes are given by coloured lines (right).

**
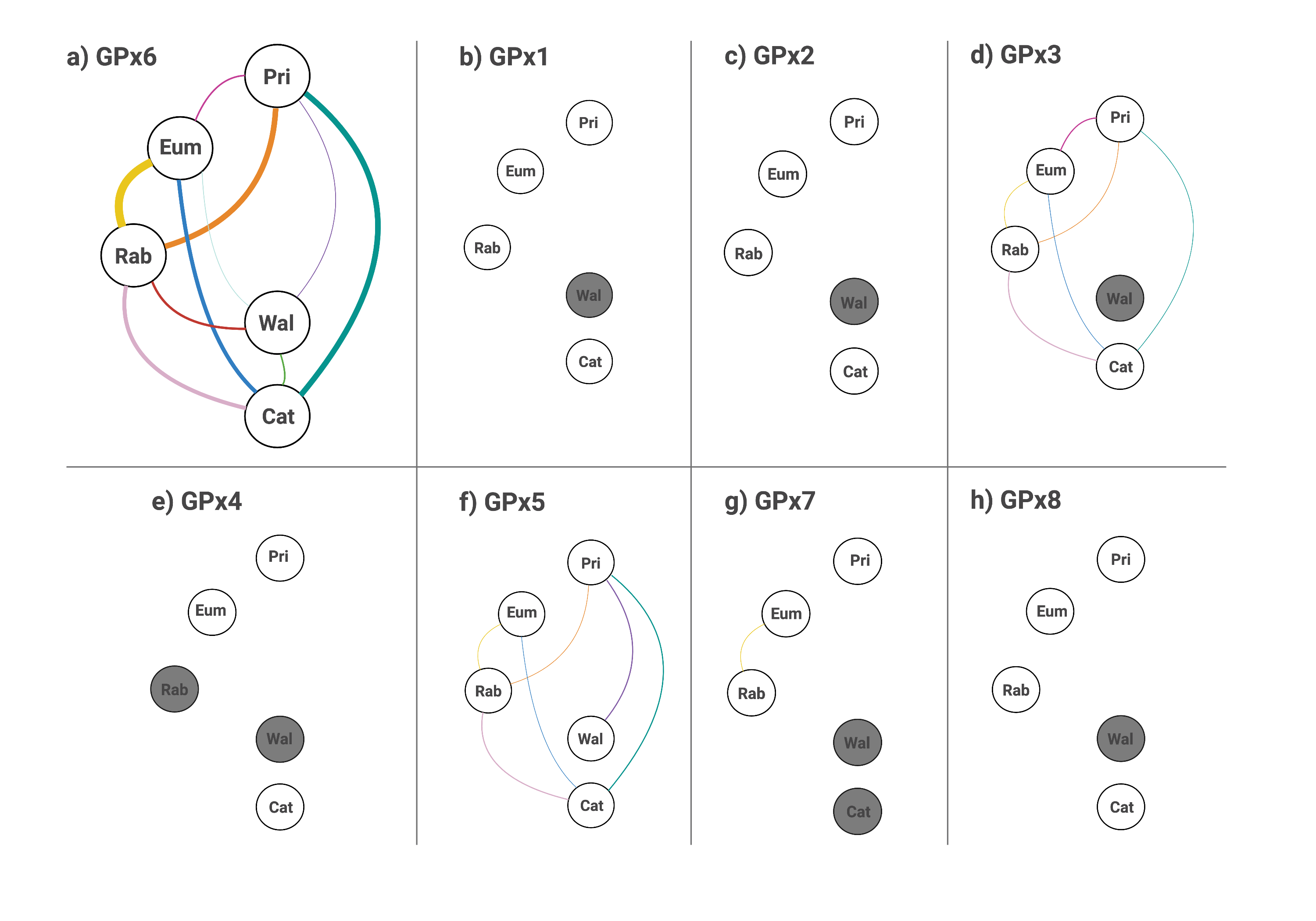

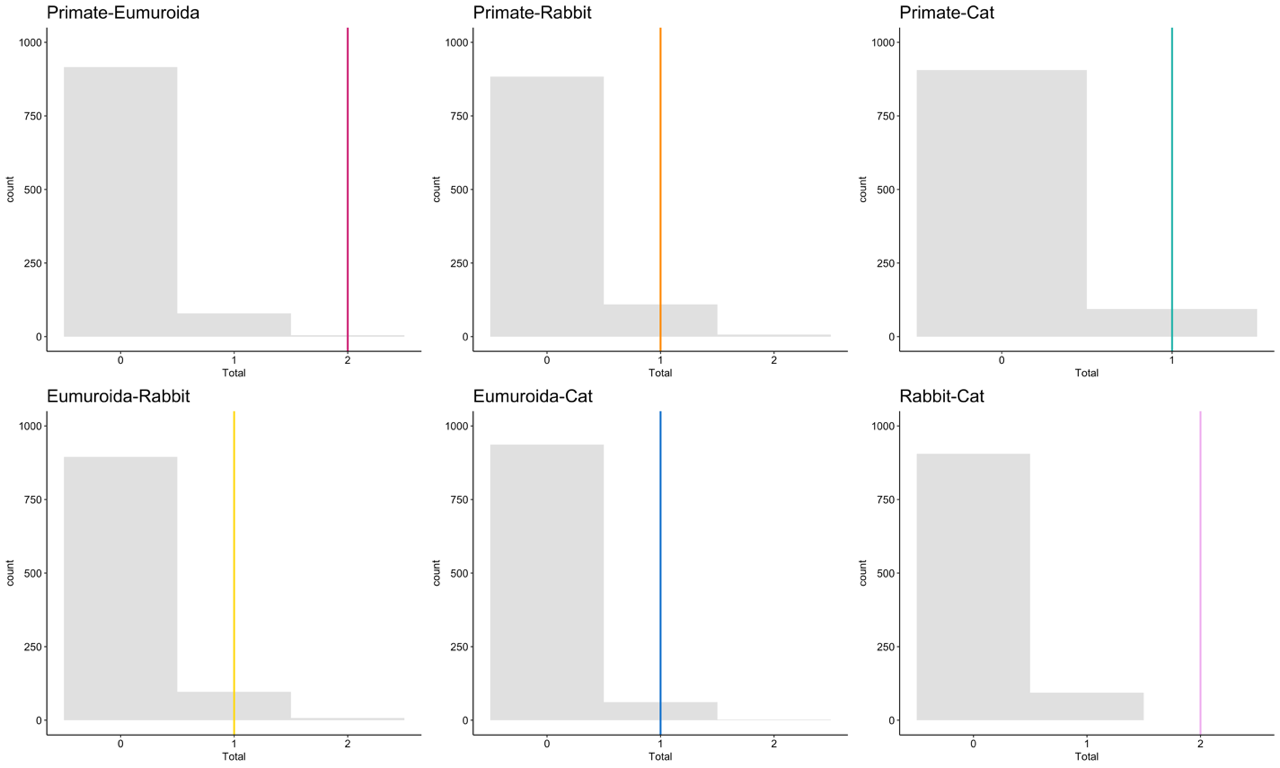
**

**Fig. S11.** Schematic representation of the number of observed convergence in the GPX3 protein between lineages where Sec is lost for Cys in GPX6, where thickness of the line represents the number of convergent changes (left). Expected distribution of convergent changes in GPX3 between lineages where Sec is lost for Cys in GPX6 according to our Seq-Gen simulations, where the observed numbers of convergent changes are given by coloured lines (right).

**
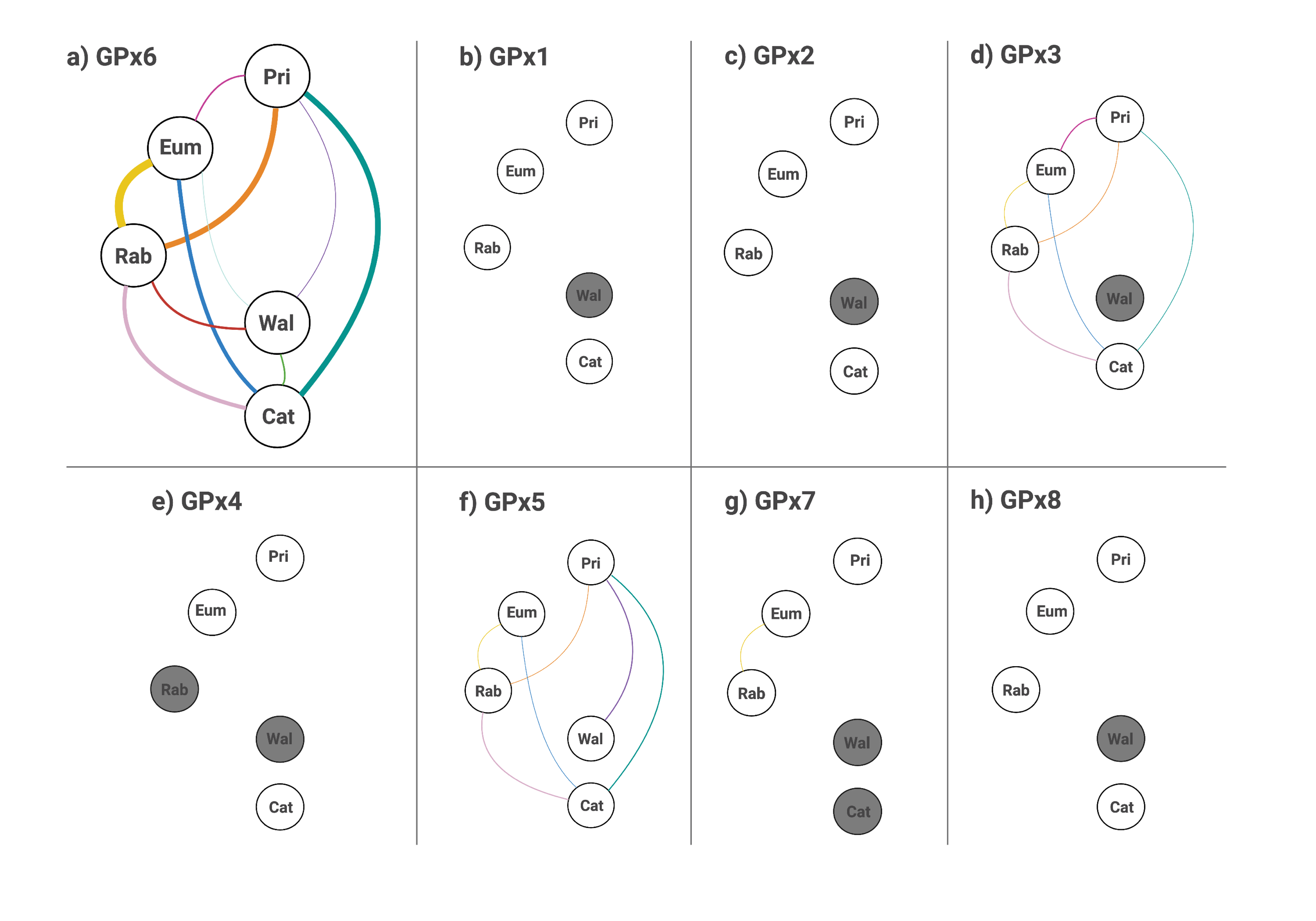
**

**
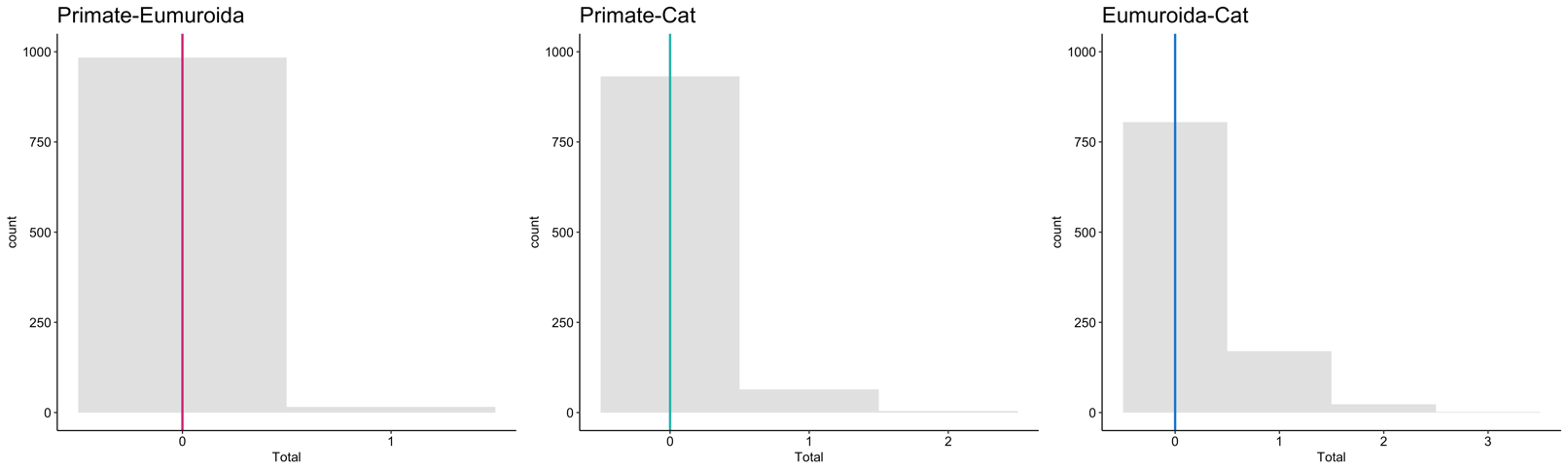
**

**Fig. S12.** Schematic representation of the number of observed convergence in the GPX4 protein between lineages where Sec is lost for Cys in GPX6, where thickness of the line represents the number of convergent changes (left). Expected distribution of convergent changes in GPX4 between lineages where Sec is lost for Cys in GPX6 according to our Seq-Gen simulations, where the observed numbers of convergent changes are given by coloured lines (right).

**
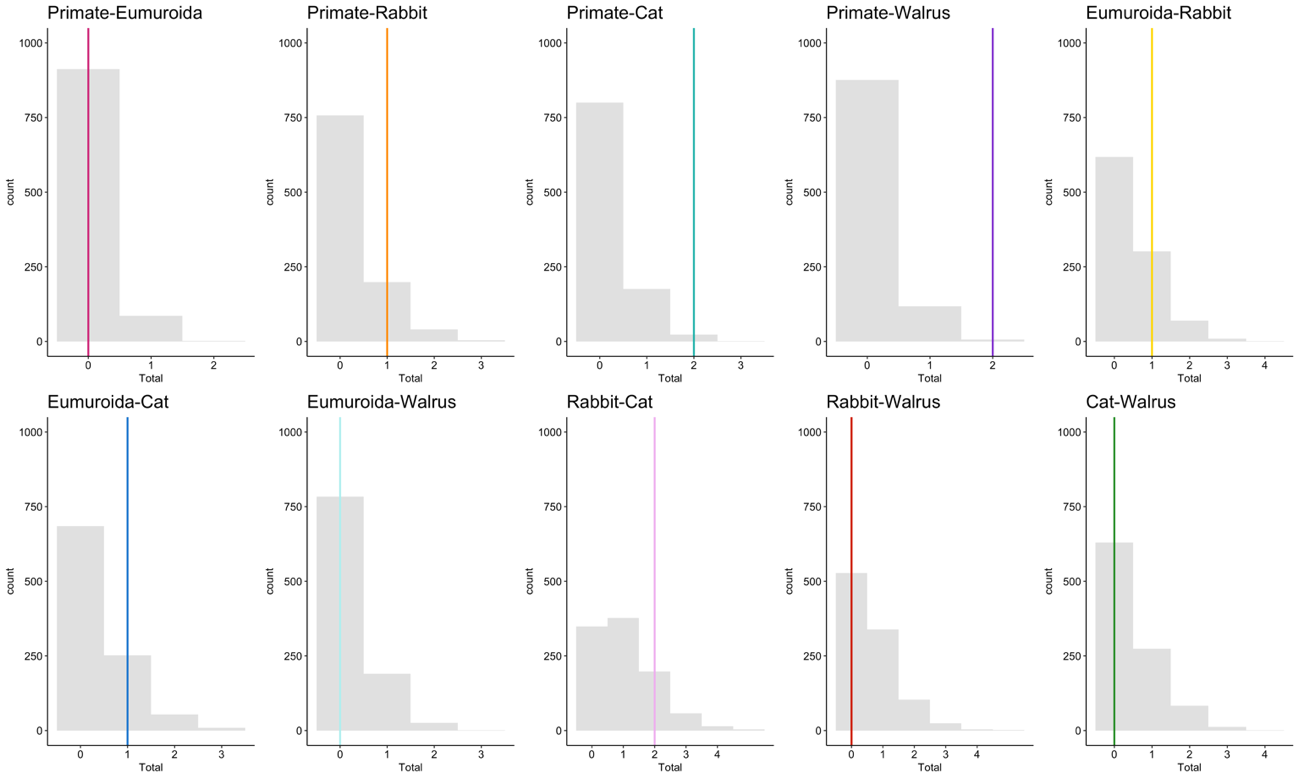

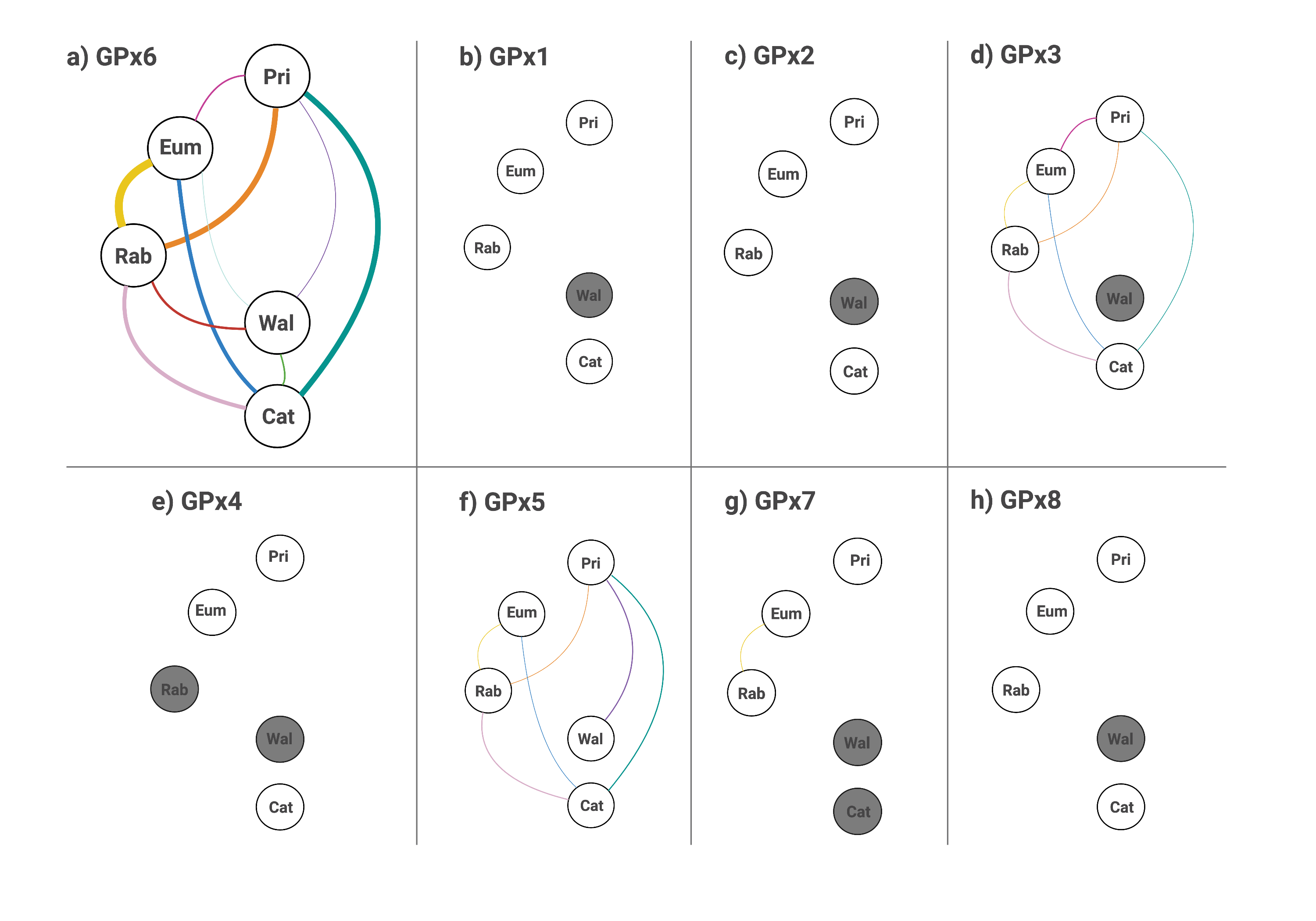
**

**Fig. S13.** Schematic representation of the number of observed convergence in the GPX5 protein between lineages where Sec is lost for Cys in GPX6, where thickness of the line represents the number of convergent changes (left). Expected distribution of convergent changes in GPX5 between lineages where Sec is lost for Cys in GPX6 according to our Seq-Gen simulations, where the observed numbers of convergent changes are given by coloured lines (right).

**
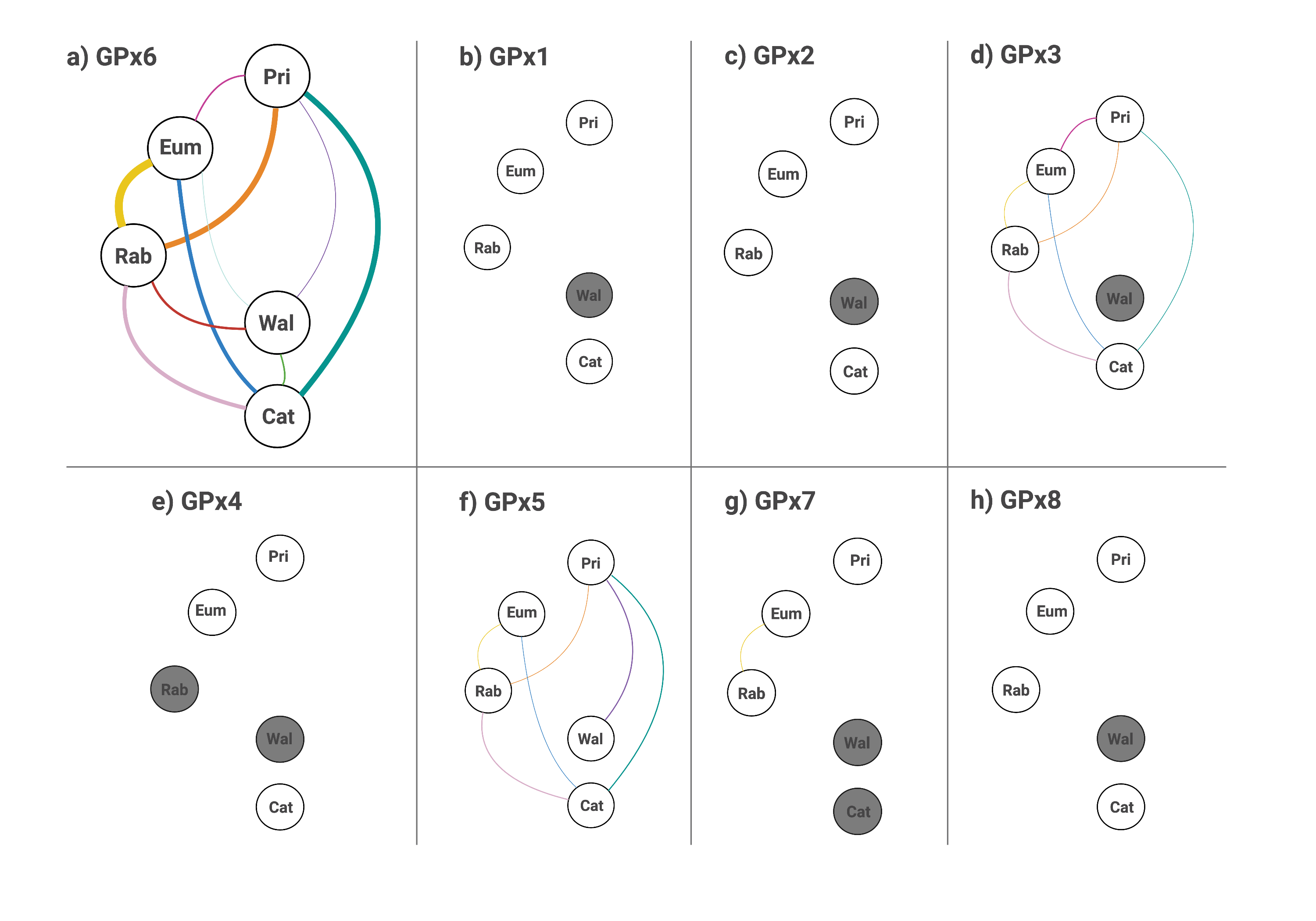
**

**
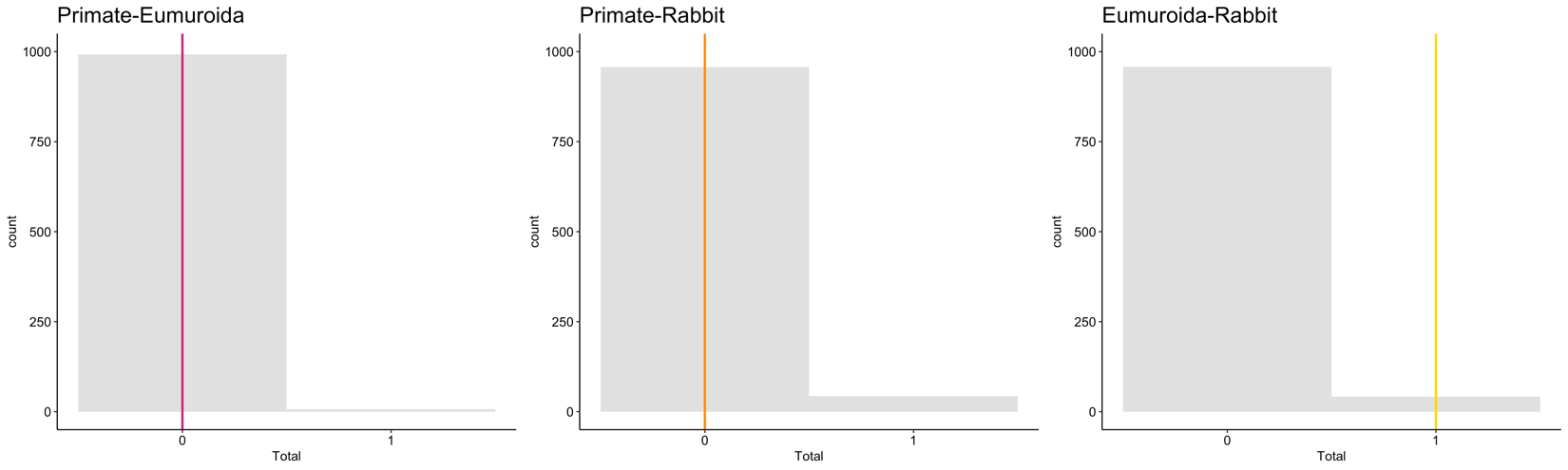
**

**Fig. S14.** Schematic representation of the number of observed convergence in the GPX7 protein between lineages where Sec is lost for Cys in GPX6, where thickness of the line represents the number of convergent changes (left). Expected distribution of convergent changes in GPX7 between lineages where Sec is lost for Cys in GPX6 according to our Seq-Gen simulations, where the observed numbers of convergent changes are given by coloured lines (right).

**
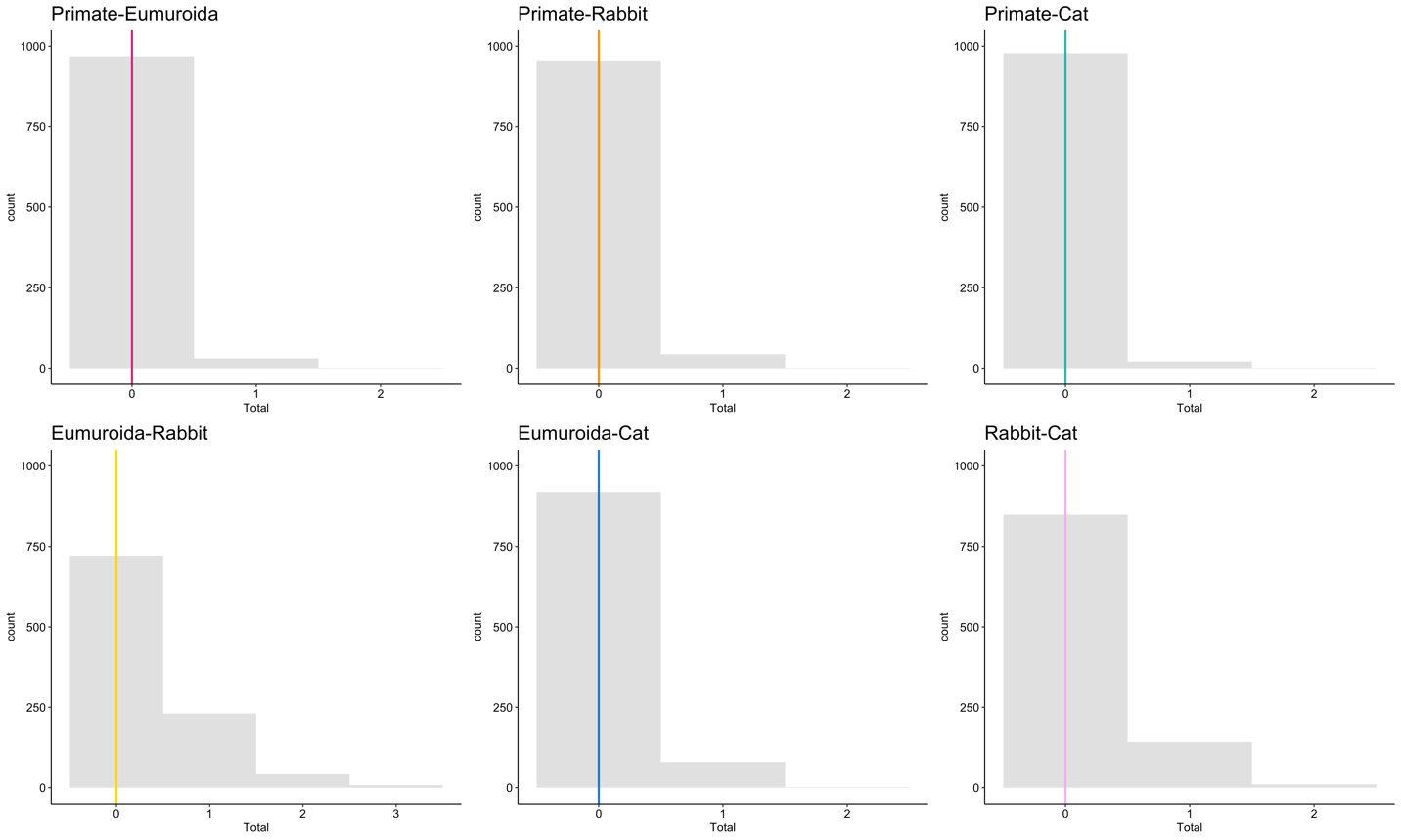
**
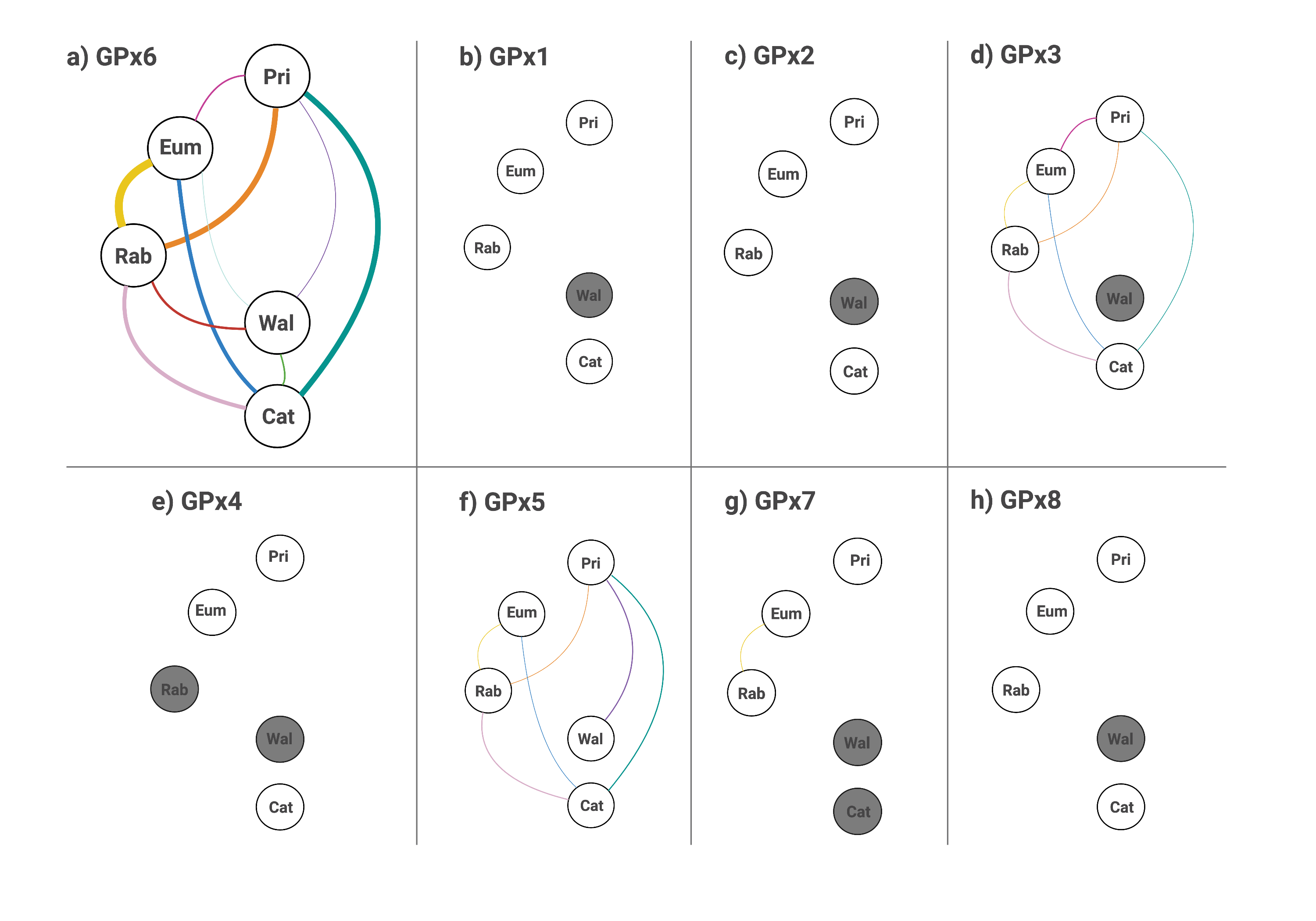


**Fig. S15.** Schematic representation of the number of observed convergence in the GPX8 protein between lineages where Sec is lost for Cys in GPX6, where thickness of the line represents the number of convergent changes (left). Expected distribution of convergent changes in GPX8 between lineages where Sec is lost for Cys in GPX6 according to our Seq-Gen simulations, where the observed numbers of convergent changes are given by coloured lines (right).


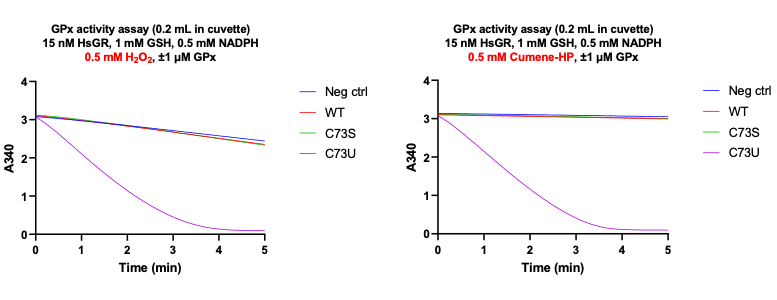


**Fig. S16**. Experimental assement of activity of mice GPX6 proteins. Left: experimental assessment of peroxidase reaction with hydrogen peroxide (H_2_O_2_)as a substrate for m-GPX6_Cys+22_ (red), m-GPX6_Sec+22_ (purple), m-GPX6_Ser+22_ (green) and a negative control (blue). Right: experimental assessment of peroxidase reaction with cumene hydroperoxide (COOH) as a substrate for m-GPX6_Cys+22_ (red), m-GPX6_Sec+22_ (purple), m-GPX6_Ser+22_ (green) and a negative control (blue).


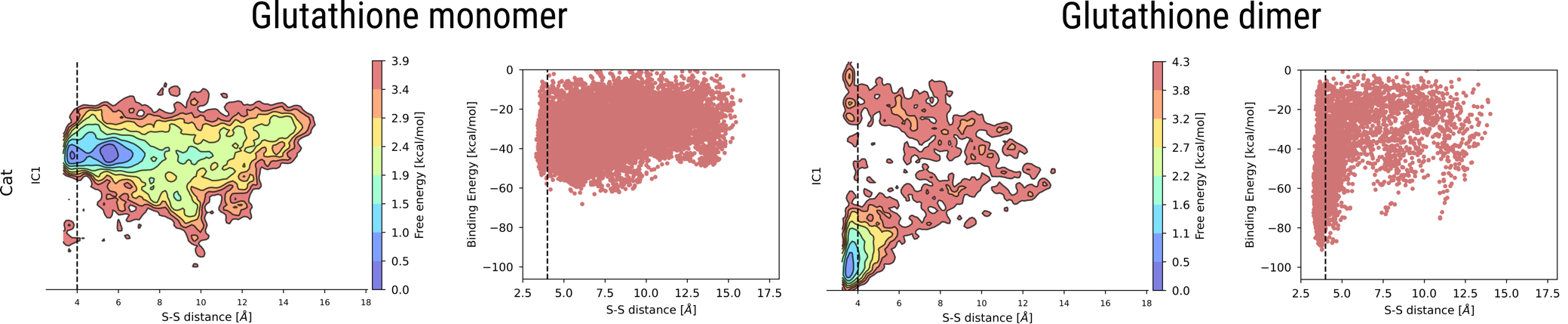


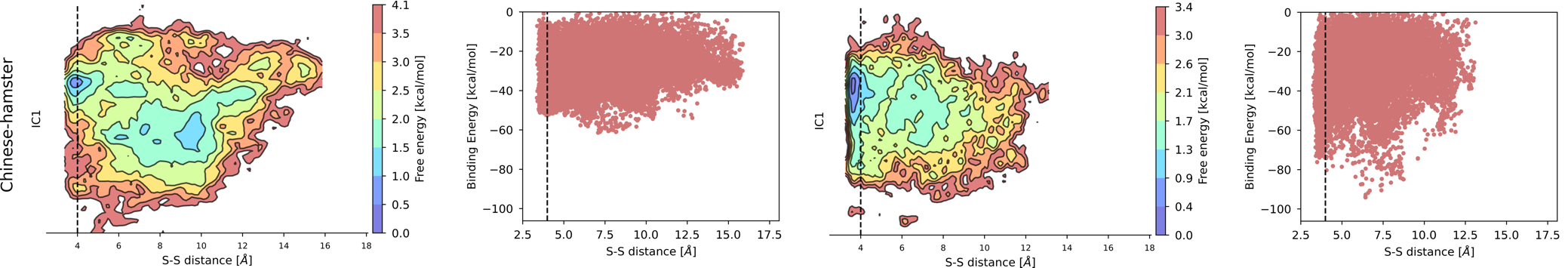


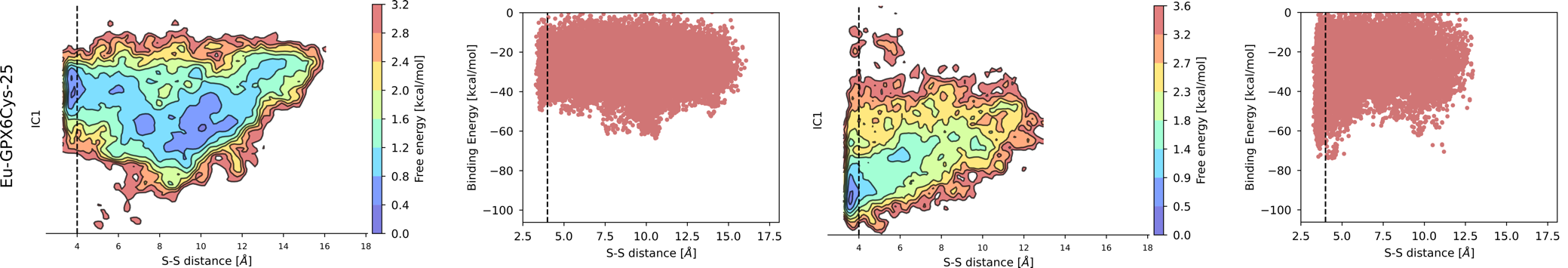


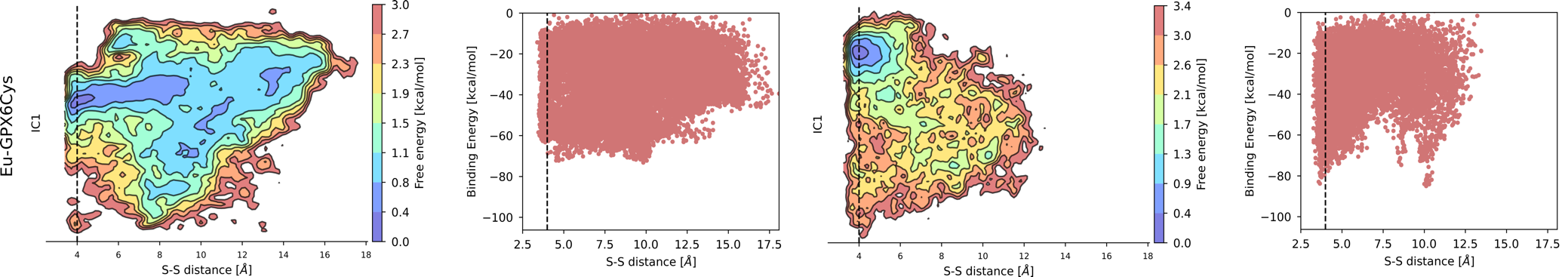


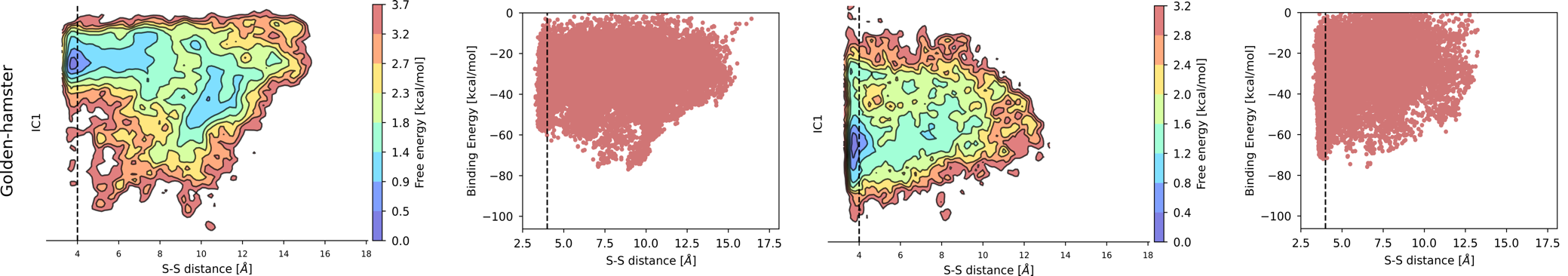


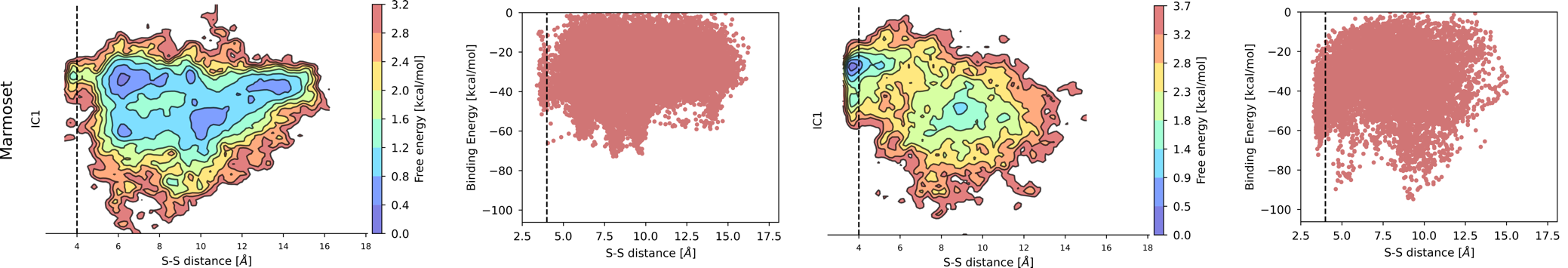


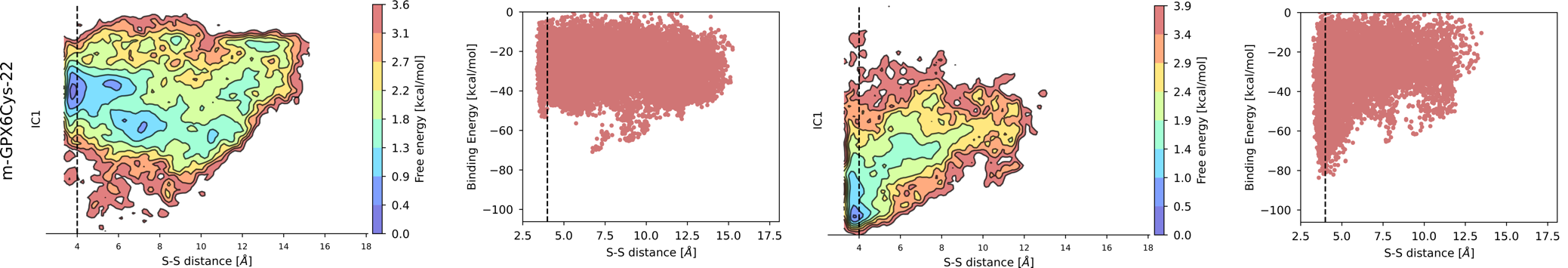


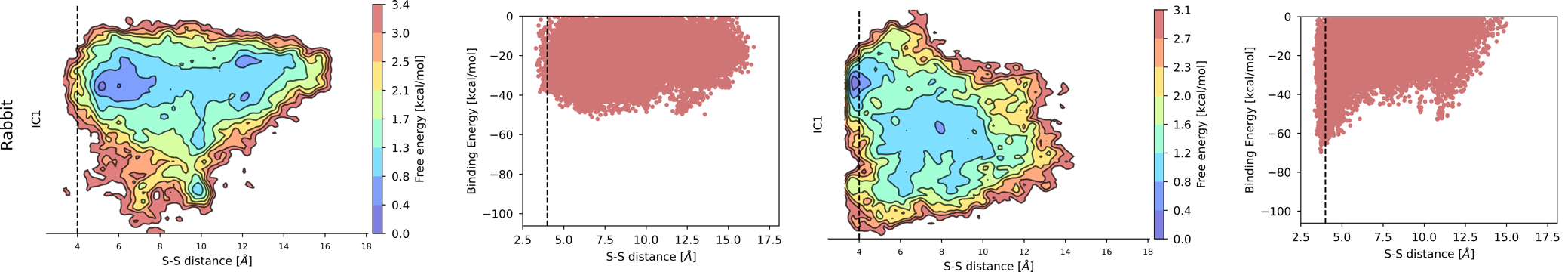

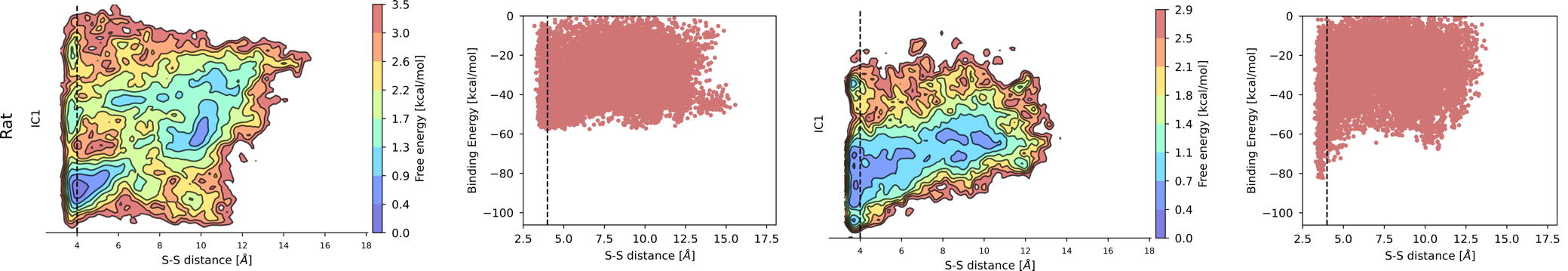


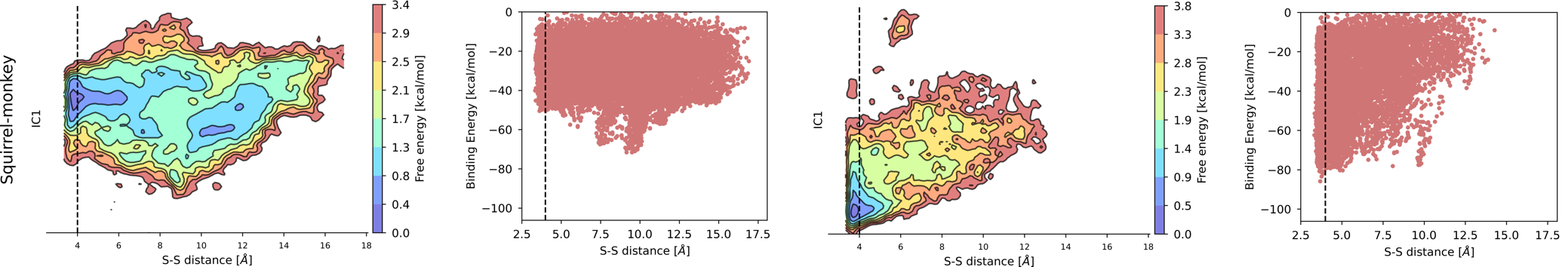


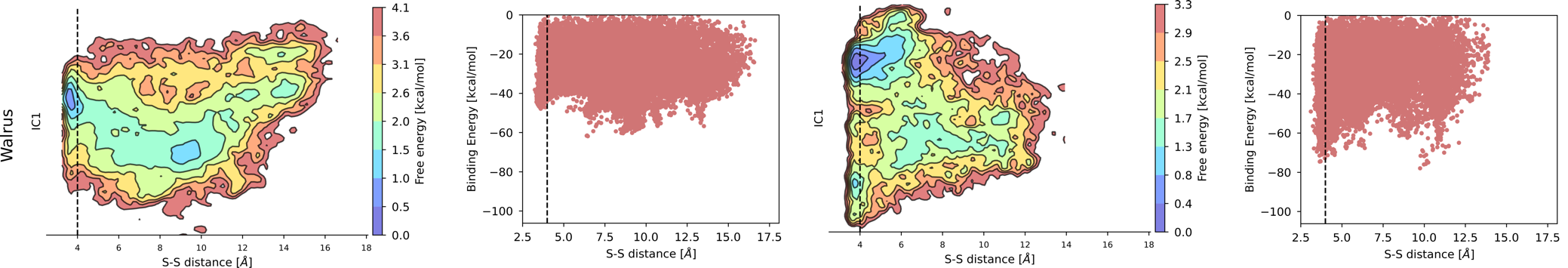


**Fig. S17**. Free energy profiles for the docking of glutathione (left) and glutathione disulfide (right) to ancestral and modern GPX6_Cys_ proteins. The x-axis represents the distance between the catalytic cysteine sulphur atom and the closest ligand’s sulphur atom, while the Y-axis shows the slowest TICA coordinate or the binding free energy. The vertical dashed line represents a distance of 4Å.

|  |  |  |  | **dN/dS in branches where GPX6 has** | | | |  |  |
| --- | --- | --- | --- | --- | --- | --- | --- | --- | --- |
| **Protein** | **Region** |  | **Sec** | | **Cys after**  **Sec was lost** | **Inherited Cys** | **All** |  | **P-value** |
|  |  |  |  | |  |  |  |  |  |
| **GPX1_Sec_** | Full length |  | 0.080 | | 0.045 | 0.087 | 0.074 |  | 0.115 |
|  | N-terminus |  | **0.043** | | **0.009** | **0.190** | **0.034** |  | **0.046** |
|  | GPX |  | 0.064 | | 0.040 | 0.069 | 0.060 |  | 0.534 |
|  | C-terminus |  | 0.085 | | 0.052 | 0.114 | 0.081 |  | 0.328 |
|  |  |  |  | |  |  |  |  |  |
| **GPX2_Sec_** | Full length |  | **0.069** | | **0.029** | **0.041** | **0.055** |  | **0.024** |
|  | N-terminus |  | 0.032 | | 0.001 | 0.001 | 0.032 |  | 0.999 |
|  | GPX |  | 0.075 | | 0.042 | 0.038 | 0.060 |  | 0.191 |
|  | C-terminus |  | 0.055 | | 0.017 | 0.048 | 0.043 |  | 0.100 |
|  |  |  |  | |  |  |  |  |  |
| **GPX3_Sec_** | Full length |  | 0.132 | | 0.131 | 0.077 | 0.125 |  | 0.222 |
|  | **N-terminus** |  | **0.241** | | **0.038** | **0.461** | **0.181** |  | **0.022** |
|  | GPX |  | 0.094 | | 0.108 | 0.056 | 0.091 |  | 0.439 |
|  | C-terminus |  | 0.105 | | 0.195 | 0.058 | 0.114 |  | 0.161 |
|  |  |  |  | |  |  |  |  |  |
| **GPX4_Sec_** | Full length |  | 0.071 | | 0.073 | 0.112 | 0.076 |  | 0.380 |
|  | N-terminus |  | 0.108 | | 0.018 | 0.123 | 0.082 |  | 0.264 |
|  | GPX |  | **0.062** | | **0.007** | **0.203** | **0.061** |  | **1x10−4** |
|  | C-terminus |  | 0.043 | | 0.003 | 0.033 | 0.030 |  | 0.126 |
|  |  |  |  | |  |  |  |  |  |
| **GPX5_Cys_** | Full length |  | 0.294 | | 0.258 | 0.429 | 0.305 |  | 0.061 |
|  | N-terminus |  | 0.678 | | 0.634 | 0.959 | 0.716 |  | 0.716 |
|  | GPX |  | 0.233 | | 0.145 | 0.219 | 0.212 |  | 0.227 |
|  | C-terminus |  | 0.237 | | 0.225 | 0.358 | 0.250 |  | 0.379 |
|  |  |  |  | |  |  |  |  |  |
| **GPX7_Cys_** | Full length |  | 0.141 | | 0.086 | 0.157 | 0.137 |  | 0.377 |
|  | N-terminus |  | 0.190 | | #999 | 0.005 | 0.122 |  | 0.070 |
|  | GPX |  | 0.083 | | 0.080 | 0.117 | 0.088 |  | 0.712 |
|  | C-terminus |  | 0.185 | | 0.080 | 0.224 | 0.178 |  | 0.242 |
|  |  |  |  | |  |  |  |  |  |
| **GPX8_Cys_** | Full length |  | 0.228 | | 0.156 | 0.156 | 0.203 |  | 0.199 |
|  | N-terminus |  | 0.169 | | 0.195 | 0.112 | 0.158 |  | 0.775 |
|  | GPX |  | 0.223 | | 0.155 | 0.198 | 0.207 |  | 0.616 |
|  | C-terminus |  | 0.217 | | 0.161 | 0.104 | 0.194 |  | 0.486 |

**Table S1**. dN/dS ratios for the GPX family and protein regions in lineages where GPX6 has Sec (Fig. 1, solid red branches), exchanged Sec for Cys (Fig. 1, dashed green branches) or inherited Cys (Fig. 1, solid green branches). In some lineages for which PAML estimates very few synonymous changes compared to the non-synonymous changes, unnaturally large dN/dS values can occur; these are marked with a #. The dN/dS ratio for all branches is the null hypothesis (one ratio for all branches) used in the likelihood ratio test contrasting the two previous ones. P-values are obtained based on a $\chi^{2}$ distribution with d.f=2. In bold, significant P-values.

| **Region** | **Foreground branches** | **P-value** | **Sites under selection** |
| --- | --- | --- | --- |
|  |  |  |  |
| **GPX domain** | Eumuroida, Rabbit, Primate, Cat, Walrus | **0.046** | 45 (0.783); 46 (0.571); **50 (0.946); 52 (0.936); 56 (0.950);** 57 (0.513); **62 (0.995);** 63 (0.535); 70 (0.552); **74 (0.992);** 75 (0.573); **77 (0.975); 83 (0.995);** 91 (0.728); **110 (0.937); 126 (0.947)**; 143 (0.799); 149 (0.606) |
|  |  |  |  |
|  |  |  |  |
| **Full protein** | Eumuroida, Rabbit | **0.006** | 16 (0.741); **45 (0.913);** 56 (0.895); **74 (0.992)**; **77 (0.942)**; **83 (0.992)**; 126 (0.822); 171 (0.784); 214 (0.573); 215 (0.668) |
|  | Eumuroida, Rabbit, Primate | **0.008** | **16 (0.988)**; 45 (0.858); **56 (0.935)**; 63 (0.501); 73 (0.503); **74 (0.991)**; **77 (0.971)**; **83 (0.988)**; **110 (0.925)**; **126 (0.937)**; 149 (0.554); 171 (0.757); 189 (0.520); 207 (0.502); 212 (0.758); 214 (0.698); 215 (0.682); 216 (0.822) |
|  | Eumuroida, Rabbit, Primate, Cat, | 0.054 |  |
|  | Eumuroida, Rabbit, Primate, Walurs | 0.050 |  |
|  | Eumuroida, Rabbit, Primate, Cat, Walrus | 0.127 |  |

**Table S2:** Foreground branches tested using the branch-site model, with the amino acid sites inferred as under selection using the Bayes Empirical Bayes inference listed when supported by a P-value < 0.05. P-values are obstained based on a $\chi^{2}$ distribution with d.f=1. Posterior probabilities of selection are shown in parehtneses, in bold face when P > 0.9. All foreground branches used here are lineages where Sec was exchanged for Cys (Fig 1, dashed green branches)

| (SM-M) | Eumuroida | 15 (GG,SA) | 166 (SK,NE) |  |  |  | |  |  |  |  | |  | |  |
| --- | --- | --- | --- | --- | --- | --- | --- | --- | --- | --- | --- | --- | --- | --- | --- |
| (SM-M) | Rabbit | 15 (GG,SS) | 34 (GG, NE) | 62 (HH,PY) | 91 (FF, LL) | 110 (TK,AR) | | 166 (SK,NR) | 212 (EE,AK) |  |  | |  | |  |
| (SM-M) | Cat | 26 (NK,DT) | **48 (NN,SS)** | 62 (HH,PV) | 90 (NP,SR) | 91 (FF,LY) | | 166 (SS,ND) | 190 (DD,HN) |  | | |  | |  |
| (SM-M) | Walrus | 91 (FF,LS) |  |  |  |  | |  |  |  | |  |  | |  |
| (SM-M) | Golden hamster | 48 (NN,ST) |  |  |  |  | |  |  |  | |  |  | |  |
| (SM-M) | Chinese hamster | 62 (HH,PY) |  |  |  |  | |  |  |  | |  |  | |  |
| (SM-M) | (Rat-mouse) | 48 (NN,SD) | 90 (QN,PS) |  |  |  | |  |  |  | |  |  | |  |
| Eumuroida | Rabbit | 15 (GG,AS) | 45 (LL,KR) | **56 (KK,QQ)** | 83 (AA,TR) | 92 (GG,NS) | | **143 (KK,NN)** | 165 (SS,TP) | 166(KK,ER) | | **167 (QQ,HH)** | | **171 (EE,DD)** |  |
| Eumuroida | Cat | **70 (TT,SS)** | **143 (KK,NN)** | 165 (SS,TP) | 166 (KS,ED) | 207(KK,QR) |  | |  |  | |  |  | |  |
| Eumuroida | Walrus | 165 (SS,TA) |  |  |  |  | |  |  |  | |  |  | |  |
| Eumuroida | Squirrel monkey | 48 (LL,KF) | **63 (VV,II)** |  |  |  | |  |  |  | |  |  | |  |
| Eumuroida | Marmoset | **70 (TT,SS)** |  |  |  |  | |  |  |  | |  |  | |  |
| Eumuroida | Rat | 127 (FY,YF) |  |  |  |  | |  |  |  | |  |  | |  |
| Eumuroida | Chinese hamster | 27 (MA,AE) |  |  |  |  | |  |  |  | |  |  | |  |
| Eumuroida | (Rat-mouse) | 27 (MA,AS) | 45 (LK,KN) | 165 (ST,PT) |  |  | |  |  |  | |  |  | |  |
| Rabbit | Cat | 62 (HH,YV) | 91 (FF,LY) | **143 (KK,NN)** | **165 (SS,PP)** | 166 (KS,RD) | |  |  |  | |  |  | |  |
| Rabbit | Walrus | 51 (YY,DH) | 91 (FF,LS) | 165 (SP,PA) |  |  | |  |  |  | |  |  | |  |
| Rabbit | Squirrel monkey | 45 (LL,FR) |  |  |  |  | |  |  |  | |  |  | |  |
| Rabbit | Marmoset | 73 (GG,AS) | **208 (SS,AA)** |  |  |  | |  |  |  | |  |  | |  |
| Rabbit | Mouse | 51 (YY,DF) | 200 (KQ,AH) |  |  |  | |  |  |  | |  |  | |  |
| Rabbit | Rat | 36 (TT,SA) | 192 (VV,IA) |  |  |  | |  |  |  | |  |  | |  |
| Rabbit | Golden hamster | 36 (TT,SA) |  |  |  |  | |  |  |  | |  |  | |  |
| Rabbit | Chinese hamster | **62 (HH,YY)** | 189 (PP,TS) |  |  |  | |  |  |  | |  |  | |  |
| Rabbit | (Rat-mouse) | 11 (PP,LS) | 45 (LK,RN) | 160 (ST,PP) | 200 (KK,AQ) |  | |  |  |  | |  |  | |  |
| Rabbit | (GH-CH) | 167 (QH,HY) | 171 (ED,DN) |  |  |  | |  |  |  | |  |  | |  |
| Cat | Walrus | 91 (FF,YS) | 165 (SS,PA) |  |  |  | |  |  |  | |  |  | |  |
| Cat | Squirrel monkey | 34 (KK,TN) | 50 (EE,GD) |  |  |  | |  |  |  | |  |  | |  |
| Cat | Marmoset | **70 (TT,SS)** |  |  |  |  | |  |  |  | |  |  | |  |
| Cat | Golden hamster | 48 (NN,TS) |  |  |  |  | |  |  |  | |  |  | |  |
| Cat | Chinese hamster | 62 (HH,YV) |  |  |  |  | |  |  |  | |  |  | |  |
| Cat | (Rat-mouse) | 48 (NN,SD) | 90 (PQ,RP) | 165 (ST,PP) |  |  | |  |  |  | |  |  | |  |
| Walrus | Mouse | 51 (YY,HF) | 54 (QQ,PN) |  |  |  | |  |  |  | |  |  | |  |
| Walrus | (Rat-mouse) | 165 (ST,AP) |  |  |  |  | |  |  |  | |  |  | |  |
| Walrus | (GH-CH) | **54 (QQ,PP)** |  |  |  |  | |  |  |  | |  |  | |  |
| Squirrel monkey | (Rat-mouse) | 45 (LK,FN) |  |  |  |  | |  |  |  | |  |  | |  |
| Marmoset | Rat | 94 (IT,TS) |  |  |  |  | |  |  |  | |  |  | |  |
| Marmoset | (Rat-mouse) | **94 (II,TT)** |  |  |  |  | |  |  |  | |  |  | |  |
| Mouse | (Rat-mouse) | 200 (QK,HQ) |  |  |  |  | |  |  |  | |  |  | |  |
| Mouse | (GH-CH) | 54 (QQ,NP) |  |  |  |  | |  |  |  | |  |  | |  |
| Rat | Golden hamster | **36 (TT,AA)** |  |  |  |  | |  |  |  | |  |  | |  |
| Rat | (Rat-mouse) | 94 (TI,ST) | 205 (IT,VI) |  |  |  | |  |  |  | |  |  | |  |
| Golden hamster | (Rat-mouse) | 48 (NN,TD) |  |  |  |  | |  |  |  | |  |  | |  |
| Chinese hamster | (Rat-mouse) | 27 (AA,ES) |  |  |  |  | |  |  |  | |  |  | |  |

**Table S3:** Convergent (sites change to the same amino acid) and pseudo-convergent (sites change to different amino acids) sites identified between GPX$6_{Cys}$ lineages by CONVERG2 (*38*). The two left-most columns are the branches between which convergent or pseudo-convergent sites are identified. The number gives the amino acid site where convergence is identified; the brackets represent the (ancestral amino acids, derived amino acids) in the order of the branches. Here, (SM-M) stands for the Squirrel monkey – marmoset internal branch, (GH-CH) stands for the Golden hamster – Chinese hamster internal branch and (rat-mouse) represents the rat-mouse internal branch. The branch names given in green indicate the branches upon which we have inferred the Sec-to-Cys exchange to have occurred. Convergent sites are in bold, all other sites are pseudo-convergent.

| Rabbit | | Mouse | | **177 (PP,SS)** |  |  |  | |  |  |  |  |  |
| --- | --- | --- | --- | --- | --- | --- | --- | --- | --- | --- | --- | --- | --- |
| Rabbit | | Rat | | **138 (AA,SS)** | **175 (QQ,KK)** |  |  | |  |  |  |  |  |
| Rabbit | | Golden hamster | | **10 (SS,NN)** | 41 (RK,ER) |  |  | |  |  |  |  |  |
| Cat | | (Mouse-Rat) | | 108 (EE,QN) |  |  |  | |  |  |  |  |  |
| Rat | | Golden hamster | | **4 (TT,AA)** |  |  |  | |  |  |  |  |  |
|  |  | |  | | | | |  |  |  |  |  |  |

**Table S4:** Convergent and pseudoconvergent sites in GPX1 between the GPX$6_{Cys}$ lineages, where the sequences were also available, as identified by CONVERG2 (*38*). The two left-most columns are the branches between which convergent or pseudo-convergent lineages are identified. The number gives the amino acid site where convergence is identified; the brackets represent the (ancestral amino acids, derived amino acids) in the order of the branches. Here, (SM-M) stands for the Squirrel monkey – marmoset internal branch, (GH-CH) stands for the Golden hamster – Chinese hamster internal branch and (rat-mouse) represents the rat-mouse internal branch. The branch names given in green indicate the branches upon which we have inferred the Sec-to-Cys exchange in GPX$6_{Cys}$ to have occurred. Strict convergent sites are given in bold, all other sites are pseudo-convergent.

| Rabbit | | Mouse | | 47 (EQ,QE) |  |  |  | |  |  |  |  |  |
| --- | --- | --- | --- | --- | --- | --- | --- | --- | --- | --- | --- | --- | --- |
|  |  | |  | | | | |  |  |  |  |  |  |
|  |  | |  | | | | |  |  |  |  |  |  |
|  |  | |  |  |  |  |  |  |  |  |  |  |  |

**Table S5:** Convergent and pseudoconvergent sites in GPX2 between the GPX$6_{Cys}$ lineages, where the sequences were also available, as identified by CONVERG2 (*38*). The two left-most columns are the branches between which convergent or pseudo-convergent lineages are identified. The number gives the amino acid site where convergence is identified; the brackets represent the (ancestral amino acids, derived amino acids) in the order of the branches. Here, (SM-M) stands for the Squirrel monkey – marmoset internal branch, (GH-CH) stands for the Golden hamster – Chinese hamster internal branch and (rat-mouse) represents the rat-mouse internal branch. The branch names given in green indicate the branches upon which we have inferred the Sec-to-Cys exchange in GPX$6_{Cys}$ to have occurred. Strict convergent sites are given in bold, all other sites are pseudo-convergent.

| Marmoset | | Eumuroida | | **5 (VV,MM)** | 172 (SA,LS) |  |  | |  |  |  |  |  |
| --- | --- | --- | --- | --- | --- | --- | --- | --- | --- | --- | --- | --- | --- |
| Marmoset | | Rabbit | | 33 (VI,LV) |  |  |  | |  |  |  |  |  |
| Marmoset | | Cat | | 5 (VV,MG) |  |  |  | |  |  |  |  |  |
| Marmoset | | (GH-CH) | | 33 (VI,LV) |  |  |  | |  |  |  |  |  |
| Eumuroida | | Rabbit | | 135 (VV,IM) |  |  |  | |  |  |  |  |  |
| Eumuroida | | Cat | | 5 (VV,MG) |  |  |  | |  |  |  |  |  |
| Rabbit | | Cat | | **126 (GG,NN)** | **152 (II,VV)** |  |  | |  |  |  |  |  |
| Rabbit | | Chinese hamster | | 152 (II,VK) |  |  |  | |  |  |  |  |  |
| Rabbit | | (GH-CH) | | **33 (II,VV)** | **107 (FF,VV)** |  |  | |  |  |  |  |  |
| Cat | | Mouse | | 154 (II,LV) |  |  |  | |  |  |  |  |  |
| Cat | | Chinese hamster | | 152 (II,VK) |  |  |  | |  |  |  |  |  |
| Cat | | (GH-CH) | | 154 (II,LV) |  |  |  | |  |  |  |  |  |
| Mouse | | (GH-CH) | | **154 (II,VV)** |  |  |  | |  |  |  |  |  |
|  |  | |  | | | | |  |  |  |  |  |  |

**Table S6:** Convergent and pseudoconvergent sites in GPX3 between the GPX$6_{Cys}$ lineages, where the sequences were also available, as identified by CONVERG2 (*38*). The two left-most columns are the branches between which convergent or pseudo-convergent lineages are identified. The number gives the amino acid site where convergence is identified; the brackets represent the (ancestral amino acids, derived amino acids) in the order of the branches. Here, (SM-M) stands for the Squirrel monkey – marmoset internal branch, (GH-CH) stands for the Golden hamster – Chinese hamster internal branch and (rat-mouse) represents the rat-mouse internal branch. The branch names given in green indicate the branches upon which we have inferred the Sec-to-Cys exchange in GPX$6_{Cys}$ to have occurred. Here, the marmoset branch is given as a branch where the Sec-to-Cys exchange in GPX$6_{Cys}$ has been estimated, given that the squirrel monkey sequence is unavailable for this protein. Strict convergent sites are given in bold, all other sites are pseudo-convergent.

No convergent sites found.

**Table S7.** Convergent and pseudoconvergent sites in GPX4 between the GPX$6_{Cys}$ lineages, where the sequences were also available, as identified by CONVERG2 (*38*).

| (SM-M) | | Rabbit | | 140 (RR,QL) |  |  |  | |  |  |  |  |  |
| --- | --- | --- | --- | --- | --- | --- | --- | --- | --- | --- | --- | --- | --- |
| (SM-M) | | Cat | | 5 (KK,RN) | **140 (RR,QQ)** |  |  | |  |  |  |  |  |
| (SM-M) | | Walrus | | 18 (AT,MS) | **29 (QQ,RR)** |  |  | |  |  |  |  |  |
| (SM-M) | | Squirrel monkey | | 151 (LV,VE) |  |  |  | |  |  |  |  |  |
| (SM-M) | | Marmoset | | 145 (SL,LI) |  |  |  | |  |  |  |  |  |
| (SM-M) | | Mouse | | 151 (LL,VM) |  |  |  | |  |  |  |  |  |
| (SM-M) | | Golden hamster | | 5 (KK,RT) |  |  |  | |  |  |  |  |  |
| (SM-M) | | Chinese hamster | | 151 (LL,VM) |  |  |  | |  |  |  |  |  |
| (SM-M) | | (GH-CH) | | 18 (AS,MA) | 148 (TS,AA) |  |  | |  |  |  |  |  |
| Eumuroida | | Rabbit | | 48 (AA,IS) |  |  |  | |  |  |  |  |  |
| Eumuroida | | Cat | | **130 (DD,NN)** |  |  |  | |  |  |  |  |  |
| Eumuroida | | Mouse | | 130 (DN,ND) |  |  |  | |  |  |  |  |  |
| Eumuroida | | Rat | | 52 (SL,LT) |  |  |  | |  |  |  |  |  |
| Eumuroida | | Golden hamster | | 17 (FL,LF) | 48 (AI,IM) | 103 (AV,VA) |  | |  |  |  |  |  |
| Eumuroida | | (Mouse-rat) | | 109 (SY,YF) |  |  |  | |  |  |  |  |  |
| Rabbit | | Cat | | 82 (EE,GK) | 140 (RR,LQ) |  |  | |  |  |  |  |  |
| Rabbit | | Squirrel monkey | | 0 (KQ,QK) |  |  |  | |  |  |  |  |  |
| Rabbit | | Rat | | **13 (DD,NN)** | 86 (KK,NE) |  |  | |  |  |  |  |  |
| Rabbit | | Golden hamster | | 0 (KK,QR) | 48 (AI,SM) |  |  | |  |  |  |  |  |
| Rabbit | | (Mouse-rat) | | **65 (GG,KK)** | **67 (YY,FF)** |  |  | |  |  |  |  |  |
| Cat | | Mouse | | 83 (KK,TN) | 130 (DN,ND) |  |  | |  |  |  |  |  |
| Cat | | Golden hamster | | 5 (KK,NT) |  |  |  | |  |  |  |  |  |
| Walrus | | Marmoset | | 21 (GK,EE) |  |  |  | |  |  |  |  |  |
| Walrus | | (Mouse-rat) | | **26 (QQ,PP)** |  |  |  | |  |  |  |  |  |
| Walrus | | (GH-CH) | | 18 (TS,SA) |  |  |  | |  |  |  |  |  |
| Squirrel monkey | | Mouse | | 151 (VL,EM) |  |  |  | |  |  |  |  |  |
| Squirrel monkey | | Golden hamster | | 0 (QK,KR) |  |  |  | |  |  |  |  |  |
| Squirrel monkey | | Chinese hamster | | 151 (VL,EM) |  |  |  | |  |  |  |  |  |
| Marmoset | | Chinese hamster | | **94 (SS,AA)** |  |  |  | |  |  |  |  |  |
| Mouse | | Chinese hamster | | **151 (LL,MM)** |  |  |  | |  |  |  |  |  |
| Mouse | | (Mouse-rat) | | 104 (TS,MT) | 155 (NK,SN) |  |  | |  |  |  |  |  |
| Golden hamster | | (GH-CH) | | 101 (IT,VI) |  |  |  | |  |  |  |  |  |
|  |  | |  | | | | |  |  |  |  |  |  |

**Table S8:** Convergent and pseudoconvergent sites in GPX5 between the GPX$6_{Cys}$ lineages, where the sequences were also available, as identified by CONVERG2 (*38*). The two left-most columns are the branches between which convergent or pseudo-convergent lineages are identified. The number gives the amino acid site where convergence is identified; the brackets represent the (ancestral amino acids, derived amino acids) in the order of the branches. Here, (SM-M) stands for the Squirrel monkey – marmoset internal branch, (GH-CH) stands for the Golden hamster – Chinese hamster internal branch and (rat-mouse) represents the rat-mouse internal branch. The branch names given in green indicate the branches upon which we have inferred the Sec-to-Cys exchange in GPX$6_{Cys}$ to have occurred. Strict convergent sites are given in bold, all other sites are pseudo-convergent.

| Marmoset | Mouse | 95 (AA,SD) |  |  |  |  |  |  |  |  |
| --- | --- | --- | --- | --- | --- | --- | --- | --- | --- | --- |
| Marmoset | (GH-CH) | 95 (AA,SD) |  |  |  |  |  |  |  |  |
| Eumuroida | Rabbit | **109 (SS,PP)** |  |  |  |  |  |  |  |  |
| Eumuroida | Chinese hamster | 116 (HR,RQ) |  |  |  |  |  |  |  |  |
| Rabbit | Mouse | 30 (HY,RH) |  |  |  |  |  |  |  |  |
| Mouse | (GH-CH) | **49 (SS,TT)** | **95 (AA,DD)** | 111 (EE,AQ) |  |  |  |  |  |  |

**Table S9:** Convergent and pseudoconvergent sites in GPX7 between the GPX$6_{Cys}$ lineages, as identified by CONVERG2 (*38*). The two left-most columns are the branches between which convergent or pseudo-convergent lineages are identified. The number gives the amino acid site where convergence is identified; the brackets represent the (ancestral amino acids, derived amino acids) in the order of the branches. Here, (SM-M) stands for the Squirrel monkey – marmoset internal branch, (GH-CH) stands for the Golden hamster – Chinese hamster internal branch and (rat-mouse) represents the rat-mouse internal branch. The branch names given in blue indicate the branches upon which we have inferred the Sec-to-Cys exchange in GPX$6_{Cys}$ to have occurred. Here, the marmoset branch is given as a branch where the Sec-to-Cys exchange in GPX$6_{Cys}$ has been estimated, given that the squirrel monkey sequence is unavailable for this protein Strict convergent sites are given in bold, all other sites are pseudo-convergent.

| Eumuroida | (GH-CH) | 12 (LF,FY) |  |  |  |  |  |  |  |  |
| --- | --- | --- | --- | --- | --- | --- | --- | --- | --- | --- |
| Rabbit | (GH-CH) | 121 (VI,IV) |  |  |  |  |  |  |  |  |
| Mouse | Chinese hamster | 59 (KK,QR) |  |  |  |  |  |  |  |  |
| Rat | (Mouse-rat) | 18 (QL,EQ) |  |  |  |  |  |  |  |  |
| Chinese hamster | (GH-CH) | 51 (MK,TM) |  |  |  |  |  |  |  |  |

**Table S10:** Convergent and pseudoconvergent sites in GPX8 between the GPX$6_{Cys}$ lineages, as identified by CONVERG2 (*38*). The two left-most columns are the branches between which convergent or pseudo-convergent lineages are identified. The number gives the amino acid site where convergence is identified; the brackets represent the (ancestral amino acids, derived amino acids) in the order of the branches. Here, (SM-M) stands for the Squirrel monkey – marmoset internal branch, (GH-CH) stands for the Golden hamster – Chinese hamster internal branch and (rat-mouse) represents the rat-mouse internal branch. The branch names given in blue indicate the branches upon which we have inferred the Sec-to-Cys exchange in GPX$6_{Cys}$ to have occurred. Strict convergent sites are given in bold, all other sites are pseudo-convergent.

**SI References**

1. F. Romagné, *et al.*, SelenoDB 2.0: annotation of selenoprotein genes in animals and their genetic diversity in humans. *Nucleic Acids Res.* **42**, D437–D443 (2014).

2. A. D. Yates, *et al.*, Ensembl 2020. *Nucleic Acids Res.* **48**, D682–D688 (2020).

3. J. W. Higdon, O. R. Bininda-Emonds, R. M. Beck, S. H. Ferguson, Phylogeny and divergence of the pinnipeds (Carnivora: Mammalia) assessed using a multigene dataset. *BMC Evol. Biol.* **7**, 216 (2007).

4. K. Katoh, J. Rozewicki, K. D. Yamada, MAFFT online service: multiple sequence alignment, interactive sequence choice and visualization. *Brief. Bioinform.* **20**, 1160–1166 (2019).

5. S. C. Potter, *et al.*, HMMER web server: 2018 update. *Nucleic Acids Res.* **46**, W200–W204 (2018).

6. R. Durbin, S. R. Eddy, A. Krogh, G. Mitchison, *Biological Sequence Analysis: Probabilistic Models of Proteins and Nucleic Acids*, 1st Ed. (Cambridge University Press, 1998) https:/doi.org/10.1017/CBO9780511790492 (January 11, 2023).

7. Z. Yang, PAML 4: Phylogenetic Analysis by Maximum Likelihood. *Mol. Biol. Evol.* **24**, 1586–1591 (2007).

8. H. J. Chatterjee, S. Y. Ho, I. Barnes, C. Groves, Estimating the phylogeny and divergence times of primates using a supermatrix approach. *BMC Evol. Biol.* **9**, 259 (2009).

9. B. M. Hallström, A. Janke, Resolution among major placental mammal interordinal relationships with genome data imply that speciation influenced their earliest radiations. *BMC Evol. Biol.* **8**, 162 (2008).

10. D. Huchon, *et al.*, Rodent Phylogeny and a Timescale for the Evolution of Glires: Evidence from an Extensive Taxon Sampling Using Three Nuclear Genes. *Mol. Biol. Evol.* **19**, 1053–1065 (2002).

11. K. Nyakatura, O. R. Bininda-Emonds, Updating the evolutionary history of Carnivora (Mammalia): a new species-level supertree complete with divergence time estimates. *BMC Biol.* **10**, 12 (2012).

12. S. J. Steppan, R. M. Adkins, J. Anderson, Phylogeny and Divergence-Date Estimates of Rapid Radiations in Muroid Rodents Based on Multiple Nuclear Genes. *Syst. Biol.* **53**, 533–553 (2004).

13. A. B. Diallo, V. Makarenkov, M. Blanchette, Ancestors 1.0: a web server for ancestral sequence reconstruction. *Bioinformatics* **26**, 130–131 (2010).

14. A. Moshe, T. Pupko, Ancestral sequence reconstruction: accounting for structural information by averaging over replacement matrices. *Bioinforma. Oxf. Engl.* **35**, 2562–2568 (2019).

15. J. Mistry, *et al.*, Pfam: The protein families database in 2021. *Nucleic Acids Res.* **49**, D412–D419 (2021).

16. S. Toppo, S. Vanin, V. Bosello, S. C. E. Tosatto, Evolutionary and structural insights into the multifaceted glutathione peroxidase (Gpx) superfamily. *Antioxid. Redox Signal.* **10**, 1501–1514 (2008).

17. Z. Yang, W. S. W. Wong, R. Nielsen, Bayes Empirical Bayes Inference of Amino Acid Sites Under Positive Selection. *Mol. Biol. Evol.* **22**, 1107–1118 (2005).

18. J. Zhang, S. Kumar, Detection of convergent and parallel evolution at the amino acid sequence level. *Mol. Biol. Evol.* **14**, 527–536 (1997).

19. S. Guindon, *et al.*, New Algorithms and Methods to Estimate Maximum-Likelihood Phylogenies: Assessing the Performance of PhyML 3.0. *Syst. Biol.* **59**, 307–321 (2010).

20. A. Rambaut, N. C. Grassly, Seq-Gen: an application for the Monte Carlo simulation of DNA sequence evolution along phylogenetic trees. *Comput. Appl. Biosci. CABIOS* **13**, 235–238 (1997).

21. Q. Cheng, E. S. J. Arnér, Selenocysteine Insertion at a Predefined UAG Codon in a Release Factor 1 (RF1)-depleted Escherichia coli Host Strain Bypasses Species Barriers in Recombinant Selenoprotein Translation. *J. Biol. Chem.* **292**, 5476–5487 (2017).

22. J. Jumper, *et al.*, Highly accurate protein structure prediction with AlphaFold. *Nature* **596**, 583–589 (2021).

23. K. W. Borrelli, A. Vitalis, R. Alcantara, V. Guallar, PELE:  Protein Energy Landscape Exploration. A Novel Monte Carlo Based Technique. *J. Chem. Theory Comput.* **1**, 1304–1311 (2005).

24. L. Molgedey, H. G. Schuster, Separation of a mixture of independent signals using time delayed correlations. *Phys. Rev. Lett.* **72**, 3634–3637 (1994).

25. M. K. Scherer, *et al.*, PyEMMA 2: A Software Package for Estimation, Validation, and Analysis of Markov Models. *J. Chem. Theory Comput.* **11**, 5525–5542 (2015).
