# Supplementary material for: Ancient Loss of Catalytic Selenocysteine Spurred Convergent Adaptation in a Mammalian Oxidoreductase
Source: Genome Biol Evol. 2024 Mar 6;16(3):evae041. doi: 10.1093/gbe/evae041 (PMC10958145; doi:10.1093/gbe/evae041)
Supplement: evae041_Supplementary_Data [file evae041_supplementary_data.zip › Supplementary_data.tar!/GPX_sequences/GPx6_input_prot.fa.rtf]

>Homo_sapiens_1
FQQFQASCLVLFFLVGFAQQTLKPQNRKVDCNKGVTGTIYEYGALTLNGEEYIQFKQFAG
KHVLFVNVAAY*GLAAQYPELNALQEELKNFGVIVLAFPCNQFGKQEPGTNSEILLGLKY
VCPGSGFVPSFQLFEKGDVNGEKEQKVFTFLKNSCPPTSDLLGSSSQLFWEPMKVHDIRW
NFEKFLVGPDGVPVMHWFHQAPVSTVKSDILEYLKQF
>Pan_troglodytes_1
FRQFQASCLVLLFLVGFAQQILKPQNRKVDCNKGVTGTIYEYGALTLNGEEYIQFKQFAG
KHVLFVNVATY*GLAAQYPELNALQEELKNFGVIVLAFPCNQFGKQEPGTNSEILLGLKY
VCPGSGFVPSFQLFEKGDVNGEKEQKVFTFLKNSCPPTSDLLGSSSQLFWEPMKVHDICW
NFEKFLVGPDGVPVMRWFHQAPVSTVKSDILEYLKQF
>Macaca_mulatta_1
IRQFQACCLVLLFLVGFAQQTLKPQNRKVDCNKRVTGTIYEYGALTLNSEEYIQFKQFAG
KHVLFVNVATY*GLAAQYPELNALQEELKNFGVVVLAFPCNQFGKQEPGTNSEILLGLKY
VRPGSGFVPNFQLFEKGDVNGEKEQKVFTFLKNSCPPTSDLLGSSSQLFWEPMKVHDIRW
NFEKFLVGPDGVPVMRWFHRAPVSTVKSDILEYLKQF
>Callithrix_jacchus_1
IRQFQASCLVPLFLVSFAQQTLKPEDRKVDCNKNVTGTIYEYGALTLSGEEYIQFKQFAG
KPVLFVNVASYCSLTAQYPELNALQEELKSLGVTVLGFPCNQFGKQEPGANSEILLGLKY
VRPGSGFVPNFQLFEKGDVNGEKEQKVFTFLKNACPPTSDLLGSSNQLFWEPMKVHDIRW
NFEKFLVGPHGVPVMRWFHRIPVSTVKADILAYLKPN
>Saimiri_boliviensis_1
IRHFQASCLVPLFLVSFAQQTLKSEDRKVDCNNNVTGTIYEYGAFTLSGEGYIQFKQFAG
KPILFVNVATYCGLTAQYPELNALQEELKSLGVIVLGFPCNQFGKQEPGANSEILLGLKY
VRPGSGFVPNFQLFEKGDVNGEKEQKVFTFLKNACPPTSDLLGSSNQLFWEPMKVHDIRW
NFEKFLVGPHGVPVMRWFHRIPVSTVKSDILAYLKP
>Tarsius_syrichta_1
IWQFWAPSLFPLFLVGFAQQTLKPQNMKVDCNKEVTGTIYEYGALTLNGEEYIQFKQYAG
KHVLFVNVATY*GLAAQYPELNALQKELKNFDVIVLGFPCNQFGKQEPGTNSEILPGLKY
VRPGGGFVPNFQLFEKGDVNGEKTKVFTFLNNSCPPTSDLLGSSSQLFWEPMKVHDIRWN
FEKFLVGPNGVPVKRWFHRAPVSTVKSDILDYLKQF
>Sus_scrofa_1
TPQFWASCLFSLCLVGFAQLIPKEQKMKMDCYKGVTGTIYEYGALTLNGEEYIPFKQYAG
KHVLFVNVATY*GLTAQYPELNALQEELKPFGVVVLGFPCNQFGKQEPAKNSEILLGLKY
VRPGGGFVPNFQLFEKGDVNGEKEQKVFTFLKNSCPPTSDLLGSSNQLFWEPMKVHDIRW
NFEKFLVGPDGVPVMRWYHRASVSTVKSDIMEYLKQF
>Bos_taurus_1
ILQLWASCLFPLFLVGLAQLTPKQQQMKVDCYKGVTGTIYEYGALTLNGEEYIQFKQYVG
KHVLFVNVATY*GLTAQYPELNALQEELKPFGVVVLGFPCNQFGKQEPAKNSEILMGLKY
VRPGGGFVPNFQLFEKGDVNGEKEQKVFTFLKNACPPTSDLLGSSSQLFWEPMKVHDIRW
NFEKFLVGPDGVPVMRWYHRASVSTVKSDMLEYLKQF
>Equus_caballus_1
IRQLWASSLFPLFLVGFAQLTPESQKMKMDCYKGVTGTIYEYGALTLNGEEYIQFKQYAG
KHVLFINVATY*GLTAQYPELNALQEELKPFGVVLLGFPCNQFGKQEPGKNSEILSGLKY
VRPGGGFVPNFQLFEKGDVNGEKEQKVFTFLKNSCPPTSDLLGSPKQLFWEPMKVHDIRW
NFEKFLVGPDGVPVMRWFHRASVSTVKSDILEYLKQF
>Loxodonta_africana_1
IRQVWASYLFSLLLVGFVQPSPNQEKMKTDCYKGVTGNIYEYGALTLNGEEYIQFKQYVG
KHVLFVNVATY*GLTAQYPELNALQEELKPFGLVVLGFPCNQFGKQEPGKNSEILSGLKY
VRPGGGFVPNFQLFEKGDVNGEKEQKIFTFLKNSCPPPSEILGSSSQLFWEPMKVHDIRW
NFEKFLVGPDGVPVMRWYHKASVSTVKSDILEYLKQF
>Odobenus_rosmarus_1
LQQLWASWLFPLFLAGLAQPTQKMKMDCYKGVTGTIYEYGALSLNEEEHIPFKRYAGKHV
LFVNVATYCGLTAQYPELNALQDELKPSGVVVLGFPCNQFGKQEPGKNSEILSGLKYVRP
GGGFVPNFQLFEKGDVNGEKEQKVFTFLKNSCPPTSDLLGSASQLFWEPMKVHDIRWNFE
KFLVGPDGVPVMRWFHKAPISTVKSDILEYLKQS
>Ictidomys_tridecemlineatus_1
NWQFQATCLFPLFLIGFCQEILKPQMVKMDCNKGVTGTIYEYGALTLSGEEYIQFKKYAG
KHVLFVNVATY*GLTAQYPELNALQEELKHLDVIVLGFPCNQFGKQEPGKNSEILSGLKY
VRPGGGFVPNFQLFEKGDVNGEKEQKVFTFLKNSCPPTSELLGSSKQLFWEPMKVHDIRW
NFEKFLVGPDGVPVMRWFHQIPVSTVKLDILEYLKQF
>Felis_catus_1
APPLWASCLFPLFLGGLAQPAPKPQTMRMDCYTGVTGTIYEYGALTLSDGDYIQFKQYAG
KVVLFVNVASYCGLTAQYPELNALQDELKRYGVVVLGFPCNQFGKQEPGKNSEILSGLKY
VRPGGGFVPSFQLFEKGDVNGENEQKVFTFLKNSCPPTSDLLGSPDQLFWEPMKVHDIRW
NFEKFLVGPNGVPVMRWFHKAPISTVRSDILEYLKQL
>Dipodomys_ordii_1
VQQFWAFYLFPLFLAGFAQETLKPQEMKVDCDKGVTGTIYEYGALTLNGEEYIKFQQYAG
KHVLFVNVATF*GLTAQYPELNALQEELKQSNVIVLGFPCNQFGKQEPGKNSEILSGLKY
VRPGGGFVPNFQLFEKGDVNGENEQKVYTFLKNSCPPTSELLGSSKQLFWEPMKIHDIRW
NFEKFLVGPDGVPVMRWFHKIPVSTVKSDIIQYLN
>Jaculus_jaculus_1
FQQFCASCLFPLFLAGLAQETLKPQKMKVDCNAGVTGTIYEYGALTLSSEEYIQFKQYAG
KHVLFVNVATY*GLTAQYPELNALQEELRPFGVVVLGFPCNQFGKQEPGKNSEILSGLKY
VRPGGGFVPNFQLFEKGDVNGDKEQKVFTFLKNACPPTSELVGSPKQLFWEPIKTHDIRW
NFEKFLVGPDGVPVMRWFHQTPVSTVKADILEYLQQS
>Otolemur_garnettii_1
IWQFWASCLFSLFLVGFTQQILKPEQMKVDCNKDVAGTIYEYGALTLDGEYIQFKQYAGK
HVLFVNVATY*GLAVQYPELNALQEDLRDFGVIILGFPCNQFGKEEPGKSEEILSGLKYV
RPGGGFVPNFQLFEKGDVNGENEQKVFTFLKNSCPPTSDVLGSSKHLFWEPMKVHDIRWN
FEKFLVGPNGVPVMRWYHRAPVSKVKSDIQEYLKQ
>Oryctolagus_cuniculus_1
IPQFWASCLFLLFLVSFAQETPEPQKMKMDCNKEVSGTIYEYGARTLNGEEDIQFQQYAG
KYVLFVNVATYCANTAQYPELNRLQEDLKNLSVIVLGFPCNQFGKQEPGRNSEILSGLKY
VRPGGNFVPNFQLFEKGDVNGENEQKVFTFLKNSCPPTSELLGSPRHLFWDPLKIHDIRW
NFEKFLVGTDGIPVMRWFHAAPVSTVKADILKYVEQF
>Cavia_porcellus_1
FLPLLLLSLAQDTPEAQRMKTDCNKTVPGTIYEYGALTLLGEEYIQFKQFAGKHVLFVNV
ATY*GLTAQYPELNALQDELRSFGVVLLGFPCNQFGRQEPGKNSEILSGLKYVRPGGGFV
PNFQLFEKGDVNGEKEQKVFTFLKNSCPPTSELLGSPRLLFWDPIRVHDIRWNFEKFLVG
PDGVPVMRWFHKAPVSVVKSDILEYLKQF
>Mus_musculus_1
AQKLWGSCLFSLFMAALAQETLNPQKSKVDCNKGVTGTVYEYGANTIDGGEFVNFQQYAG
KHILFVNVASFCGLTATYPELNTLQEELKPFNVTVLGFPCNQFGKQEPGKNSEILLGLKY
VRPGGGYVPNFQLFEKGDVNGDNEQKVFSFLKNSCPPTSELFGSPEHLFWDPMKVHDIRW
NFEKFLVGPDGVPVMRWFHHTPVRIVQSDIMEYLNQT
>Rattus_norvegicus_1
TQQFWGPCLFSLFMALAQETLDPQKSKVDCNKGVAGTVYEYGANTLDGGEYVQFQQYAGK
HILFVNVASFCGLTATYPELNTLQEELRPFNVSVLGFPCNQFGKQEPGKNSEILLGLKYV
RPGGGFVPNFQLFEKGDVNGDNEQKVFSFLKSSCPPTSELLGSPEHLFWDPMKVHDIRWN
FEKFLVGPDGAPVMRWFHQTPVRVVQSDIMEYLNQT
>Mesocricetus_auratus_1
AQAFWASCLFPLFLAALAQETLNLQKAKVDCNKGVAGTIYEYGAKTLTGGEYVPFQQYAG
KHILFVNVASFCGLTATYPELNTLQEELKQFNVIVLGFPCNQFGKQEPAKNSEILLGLKY
VRPGGGYVPNFQLFEKGDVNGENEQKIFSFLKNSCPPTSELLGSTEYLFWNPMRVHDIRW
NFEKFLVGPDGVPVMRWFHKTPVSTVQSDIMEYLNQS
>Cricetulus_griseus_1
AQPFWVSCLFPLFLAALAQETLNPQKEKVNCNKGVTGTIYDYGAKTLNGGEYVPFQQYAG
KYILFVNVASFCGLTATYPELNTLQEELKQFNVIVLGFPCNQFGKQEPAKNSEILLGLKH
VRPGGGYVPNFQLFEKGDVNGENEQKIFSFLKNSCPPTSELLGSTEYLFWNPMRVHDIRW
NFEKFLVGSDGVPVMRWFHKTPVSTVQSDIMEYLNQS
